# Genome Assembly Improvement and Mapping Convergently Evolved Skeletal Traits in Sticklebacks with Genotyping-by-Sequencing

**DOI:** 10.1534/g3.115.017905

**Published:** 2015-06-03

**Authors:** Andrew M. Glazer, Emily E. Killingbeck, Therese Mitros, Daniel S. Rokhsar, Craig T. Miller

**Affiliations:** Molecular and Cell Biology Department, University of California-Berkeley, Berkeley, California 94720

**Keywords:** genotyping-by-sequencing, linkage map, quantitative trait locus, convergent evolution, stickleback

## Abstract

Marine populations of the threespine stickleback (*Gasterosteus aculeatus*) have repeatedly colonized and rapidly adapted to freshwater habitats, providing a powerful system to map the genetic architecture of evolved traits. Here, we developed and applied a binned genotyping-by-sequencing (GBS) method to build dense genome-wide linkage maps of sticklebacks using two large marine by freshwater F2 crosses of more than 350 fish each. The resulting linkage maps significantly improve the genome assembly by anchoring 78 new scaffolds to chromosomes, reorienting 40 scaffolds, and rearranging scaffolds in 4 locations. In the revised genome assembly, 94.6% of the assembly was anchored to a chromosome. To assess linkage map quality, we mapped quantitative trait loci (QTL) controlling lateral plate number, which mapped as expected to a 200-kb genomic region containing *Ectodysplasin*, as well as a chromosome 7 QTL overlapping a previously identified modifier QTL. Finally, we mapped eight QTL controlling convergently evolved reductions in gill raker length in the two crosses, which revealed that this classic adaptive trait has a surprisingly modular and nonparallel genetic basis.

Understanding the genetic basis of adaptation remains a major unsolved goal in biology. For example, when the same phenotype evolves in independent lineages (convergent evolution), is the genetic basis predictable (Stern and Orgogozo 2009)? Do evolved loci typically affect phenotypes in a global or in a modular, anatomically specific manner (Wagner *et al.* 2007)? In systems where differently adapted natural populations are interfertile, quantitative trait locus (QTL) mapping provides an entry point to study the genetic architecture of evolved traits.

The threespine stickleback (*Gasterosteus aculeatus*) has undergone widespread adaptive radiation in which marine fish independently colonized and adapted to countless freshwater habitats (Bell and Foster 1994). Marine and freshwater populations typically differ in many skeletal phenotypes, including a reduction of the number of lateral plates, which are used for defense against predation (Reimchen 1992; Hagen 1973) and a reduction in the length of gill rakers, which comprise a set of bones used for prey retention during feeding (Schluter 2000). Lateral plate reduction in many freshwater populations is largely controlled by a large-effect QTL on chromosome 4 (Berner *et al.* 2014; Colosimo *et al.* 2004; Cresko *et al.* 2004; Liu *et al.* 2014; Wark *et al.* 2012), which has been shown to be a regulatory allele of *Ectodysplasin* (*Eda*) (Colosimo *et al.* 2005; O’Brown *et al.* 2015). Several smaller-effect modifier QTL also contribute to plate number reduction (Colosimo *et al.* 2004; Wark *et al.* 2012). Reductions in gill raker length and number are important trophic adaptations in freshwater sticklebacks and other postglacial fish, and have convergently evolved multiple times (Schluter 2000). Typically, fish that eat small plankton evolve more and longer gill rakers and fish that eat larger prey evolve fewer and shorter gill rakers (Arnegard *et al.* 2014; Kahilainen *et al.* 2011; Schluter and McPhail 1992; Theis *et al.* 2014). Our previous genetic studies found gill raker number to be a highly polygenic trait, controlled by more than 15 QTL (Glazer *et al.* 2014; Miller *et al.* 2014). Although stickleback gill raker length differences can arise due to phenotypic plasticity, there is a large heritable component (Day *et al.* 1994; Hatfield 1997). The genetic basis of evolved freshwater reductions in stickleback gill raker length is poorly understood, but two QTL were identified in a cross between European lake and stream populations (Berner *et al.* 2014).

The stickleback genome has been sequenced and scaffolds were anchored in the stickleback reference assembly (Jones *et al.* 2012) using a linkage map made from an F2 cross of 92 fish. The assembly consists of 113 anchored scaffolds on 21 chromosomes, as well as 1822 unanchored scaffolds (13.2% of the assembly). Subsequent work inverted the orientations of 13 anchored scaffolds and anchored 18 additional scaffolds (Roesti *et al.* 2013). Three large chromosomal inversions are typically present between marine and freshwater sticklebacks (Jones *et al.* 2012), but the extent of other differences between stickleback populations in genomic structure and genome-wide recombination patterns is largely unknown.

Here, we used genotyping-by-sequencing (GBS) to sample approximately 100,000 SNPs to low coverage (approximately 1.5× per sample) for more than 350 sticklebacks in each of two marine × freshwater F2 crosses. We binned these low-coverage SNPs into more than 1000 high-coverage (approximately 150×) markers. Using these markers, we constructed high-density genome-wide linkage maps, which we used to anchor, reorient, and rearrange scaffolds and to examine genome-wide recombination patterns. We also used these maps to map the genetic basis of two ecologically important phenotypes. First, as a positive control, we mapped QTL controlling lateral plate number. Second, we mapped gill raker length QTL to test two hypotheses: that convergent evolution in two independently derived freshwater populations involves similar genetic architectures, and that gill raker length is genetically controlled by modular QTL affecting the lengths of subsets of gill rakers.

## Materials and Methods

### Stickleback crosses

Two marine × freshwater F1 crosses were previously described (Glazer *et al.* 2014). A wild-caught male marine fish from the Little Campbell River (British Columbia, Canada; LITC) was crossed to a wild-caught female freshwater fish from Fishtrap Creek (Washington state; FTC) to generate the FTC cross. A male freshwater fish from Bear Paw Lake (Alaska; BEPA; lab-reared offspring of wild-caught parents) was crossed to a wild-caught marine female LITC fish to produce the BEPA cross. F1s were intercrossed to generate 360 and 363 F2 fish from the FTC and BEPA crosses, respectively. Fish with low genotype coverage (n = 2 from each cross) were removed from the analysis. See Supplemental Methods in Supporting Information, File S8 for additional information on raising the crosses.

### Animal statement

Wild anadromous marine fish were collected from the Little Campbell River in British Columbia under a fish collection permit from the British Columbia Ministry of Environment (permit #SU08-44549). Wild freshwater fish were collected from Fishtrap Creek in Washington under a fish scientific collection permit from the Washington Department of Fish and Wildlife (permit #08-284). All animal work was approved by the Institutional Animal Care and Use Committees of the University of California-Berkeley or Stanford University (protocol number R330 and 13834).

### Preparation of GBS libraries

DNA was isolated by phenol-chloroform extraction or with a DNeasy 96 Blood and Tissue Kit (Qiagen). Genomic DNA concentration was assessed with a NanoDrop 1000 spectrophotometer (Thermo Scientific) and by Quant-iT PicoGreen Assay (Invitrogen). Unless otherwise noted, GBS Illumina sequencing libraries were constructed as previously described (Elshire *et al.* 2011); 50 ng/sample of genomic DNA was used. Individuals were sequenced in seven libraries (Table 1). For libraries 1–3, 48 barcode plus common adapters were used (Elshire *et al.* 2011). Libraries 4–6 used 96 ApeKI Y-shaped adapters with internal barcodes (ICGMC 2015), and library 7 used these 96 Y-shaped adapters and 4 different PCR primers with different index barcodes (384 total samples) (adapted from Peterson *et al.* 2012) (Table S1, Figure S1). For library 7, all volumes in the digestion and ligation reactions were successfully halved relative to the Elshire *et al.* (2011) protocol to reduce reagent costs. ApeKI digestion, ligation, and sample clean-up were performed as described (Elshire *et al.* 2011). PCR amplification of sequencing libraries was performed in 50 ul reactions with 25 ul Taq 2× Master Mix (NEB), 50–450 ng of primer, and 2 ul of each library at 98° for 30 sec, 10–22 cycles at 98° for 10 sec, 65° for 30 sec, 72° for 30 sec, 5 min at 72°, and held at 4°. Primer concentration and cycle number were varied to amplify enough product for sequencing. PCR products were purified and size-selected with AMPure XP beads (Agencourt) with a bead:sample ratio of 0.7. Libraries were analyzed on an Agilent 2100 Bioanalyzer High-Sensitivity Chip for quality control and sequenced with 100 bp paired-end sequencing on an Illumina HiSeq 2000 sequencer.

**Table 1:** Summary of GBS libraries

Library	Barcodes	F2s	Adapters	Total Reads	Barcoded F2 Reads	Barcoded Reads/F2	Mapped Reads/F2	Mapped Read COV	SNP Coverage	Marker Coverage	Genotype Fail %
1*^a^*	48	12	Elshire	216	58.8	4.9	3.1	0.5	3.1	371.7	0.04
2*^a^*	48	48	Elshire	212.4	168.3	3.5	2.4	1.03	2.4	288.2	0.5
3	48	48	Elshire	418.6	397.7	8.3	5.6	1.18	0.7	85.1	5.4
4	96	96	Y-shaped	559.7	487.6	5.1	3.9	0.61	3.5	424.7	0.3
5	96	96	Y-shaped	428.2	363.1	3.8	3	0.59	2.8	228.5	1.1
6	96	96	Y-shaped	440.5	371.9	3.9	3	0.61	2.8	230.5	0.9
7*^b^*^,^*^c^*	384	332	Y-shaped	498	259.1	0.8	0.6	0.81	0.5	49.1	2.6
All	816	719	*	2773.3	2101.1	2.9	2.2	1.26	1.7	168.6	1.9

For each library, the number of barcodes and F2 fish included is listed. Total reads, barcoded F2 reads, barcoded reads/F2, and mapped reads/F2 are reported in units of millions of reads. Genotype fail % indicates percentage of final genotypes that were missing. COV, coefficient of variance (mean/standard deviation).

a For libraries 1 and 2, the R2 read failed, resulting in half the expected number of reads.

b ApeKI digestion and adapter ligation reactions were performed at half volume to conserve costs.

c Library 7 included nine F2 samples that were sequenced in libraries 1–6 but had very low sequencing coverage.

### Processing reads from GBS libraries

**Figure 1:**
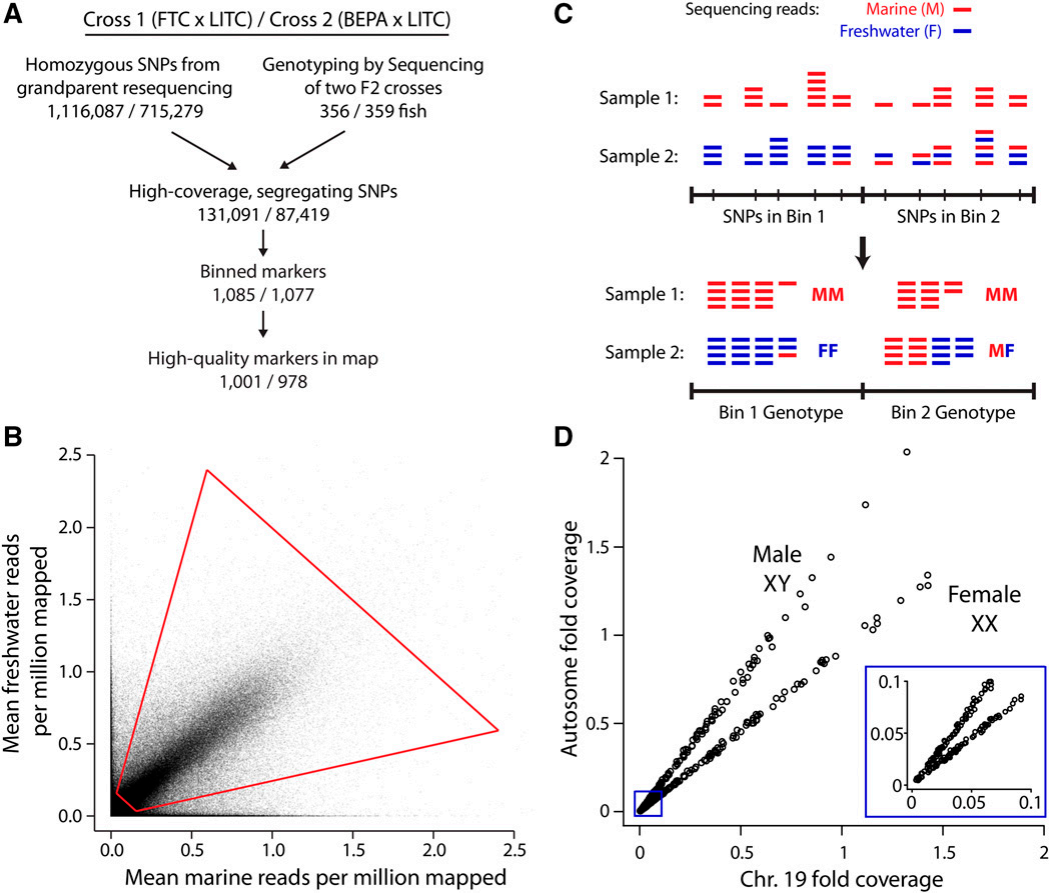
Genotyping-by-sequencing (GBS) approach. (A) Flowchart of GBS. For each cross, the two grandparents were resequenced to determine homozygous SNP differences, which were filtered for high coverage levels and expected allele ratios in F2s (see *Materials and Methods*). (B) Sieve for high-coverage, segregating SNPs. For each SNP, the mean number of mapped reads supporting the marine and freshwater alleles, normalized for the number of millions of reads mapped per sample, is displayed. Data are shown for the FTC × LITC cross. Sieve is shown with red quadrilateral: freshwater allele frequency between 0.2 and 0.8 and total coverage between 0.2 and 3. (C) Diagram of binning approach. Low-coverage sequencing generated read pileup at a large number of SNPs. For each F2, SNPs were binned by counting the total number of marine and freshwater reads within the bin and determining a genotype from the pooled counts. Sample 2 illustrates a case in which a recombination breakpoint is near the boundary between two bins and Bin 1 containing the breakpoint is considered to have the FF genotype. Alternatively, bins containing recombination breakpoints also frequently were called with uncertain MF/FF or MM/MF genotypes (Figure S2). (D) Calling sex from sex chromosome (chromosome 19) coverage. Females (XX) have approximately equal sex chromosome and autosome coverage levels, whereas males (XY) have approximately half the coverage level on the sex chromosome compared to the autosomes. Data are shown for the FTC cross. Inset shows zoom-in of low-coverage samples showing that female and male fish can still be distinguished.

The two grandparents of the FTC cross and the two grandparents of the BEPA cross were resequenced to approximately 60× and 6× coverage, respectively. In each cross, sites where one grandparent was homozygous for one allele and the other grandparent was homozygous for a second allele were identified (“homozygous SNP positions”; see Supplemental Methods in File S8). GBS reads from F2s were sorted by barcode with a custom Perl script. Reads were mapped to the stickleback reference genome with BWA using default settings (www.bio-bwa.sourceforge.net), allowing up to a 4% difference between reads and the reference genome. We devised a method to identify high-quality, segregating SNPs. For each homozygous SNP position, F2 GBS reads overlapping the SNP were considered, and the number of reads supporting marine and freshwater alleles for each homozygous SNP position was determined with SAMtools (www.samtools.sourceforge.net). Genomic positions identified as not having a homozygous difference in the grandparent resequencing were not examined in the F2s. For each homozygous SNP position, a weighted average of these values was calculated across all F2s, normalized by the total number of mapped reads for each F2. We multiplied the marine and freshwater weighted averages by 10^6^ to calculate reads per million mapped (RPMM). Properly segregating SNPs should have an approximately 1:1 ratio of marine:freshwater alleles, as was observed for most SNPs (Figure 1B). However, we observed some genomic regions that had a skewed allele ratio in the F2s, possibly due to meiotic drive and/or the lethality of particular genotypic classes. For example, a region of chromosome 2 centered at marker 0_32 in the FTC cross had a freshwater allele frequency of 0.61 (File S1). Therefore, a wider range of allele ratios was allowed for individual SNPs (a marine/freshwater RPMM ratio between 4:1 and 1:4). To include SNPs with true segregation bias, skewed markers adjacent to other similarly skewed markers were included, but skewed markers surrounded by nonskewed markers were removed (see Supplemental Methods in File S8). Additionally, SNPs were filtered for those with an average marine plus freshwater RPMM between 0.2 and 3.0 to have a set of SNPs with similar coverage levels. A separate set of sieving parameters was used to determine sex chromosome genotypes (see Supplemental Methods in File S8).

These filtered SNPs were further grouped into bins of at most 500 kb. Bin size was scaled to divide each scaffold into evenly sized bins. Scaffolds smaller than 100 kb were binned into one bin, scaffolds between 100 kb and 1 Mb were binned into two bins of equal size, scaffolds between 1 Mb and 1.5 Mb were divided into three bins, scaffolds between 1.5 and 2 Mb were divided into four bins, and so on. For each SNP within a bin, marine and freshwater read counts were summed and genotypes were called (see Supplemental Methods in File S8). Fish that had missing genotypes for more than 50% of markers were removed from the analysis (n = 2 in each cross). Nine additional samples with high rates of missing genotypes in libraries 1–6 were resequenced successfully in library 7. Markers that had missing data for at least 20% of fish were removed from the analysis (n = 59 and 39 in the FTC and BEPA crosses, respectively). Markers were also removed that had aberrant allelic ratios (n = 25 and 60 in the FTC and BEPA crosses, respectively; see Supplemental Methods in File S8). Genetic linkage maps were created with JoinMap 4.0 (Kyazma) using regression mapping with default settings. Further information on creating a consensus scaffold map, a second method for anchoring scaffolds, calling sex of the F2s, and fine mapping of recombinant breakpoints are presented in Supplemental Methods in File S8.

GBS and grandparental sequence reads are available in the Sequence Read Archive (accession number SRP057885). File S5, File S6, and File S7 and a script to convert from original to revised genome coordinates are available from the Dryad Digital Repository (http://dx.doi.org/10.5061/dryad.q018v).

### Phenotyping

Lateral plate number and gill raker length were measured from Alizarin-stained fish (see Supplemental Methods in File S8). The average of plate counts on the left and right sides was used for QTL mapping. To phenotype gill raker length, branchial skeletons were dissected out of fish and mounted flat on bridged coverslips as described (Miller *et al.* 2014). Measurements were obtained by acquiring digital images of left side row 1 ventral gill rakers on a Leica M165 microscope and tracing a line segment from the gill raker base to tip in imageJ (Schneider *et al.* 2012). Three gill raker lengths were measured on the first ceratobranchial: lateral (second gill raker from end near ventral/dorsal joint), middle (middle of ceratobranchial), and medial (second gill raker from end near midline).

### QTL mapping

For QTL mapping, plate number and gill raker length phenotypes were tested for an association with standard length and sex by linear regression in R (www.r-project.org) and corrected for size, sex, and/or log-transformed, when appropriate (see Supplemental Methods in File S8). QTL mapping was performed in R/qtl (Broman and Sen 2009; Broman *et al.* 2003). Initial QTL mapping was performed with *scanone* with Haley-Knott regression. Trait-specific genome-wide significance thresholds with α of 0.05 were calculated with 1000 permutations. In cases where multiple significant QTL affected a phenotype, multiple QTL mapping was performed with *stepwiseqtl*, QTL peak markers, and LOD plots calculated with *refineqtl*, and peak LOD scores and percent variance explained values calculated with *fitqtl*, adjusting for the effect of other QTL underlying the phenotype when appropriate.

## Results

### Two genome-wide linkage maps

To build genome-wide linkage maps in two marine × freshwater stickleback F2 crosses, we used a binned GBS approach (modified from Elshire *et al.* 2011; outlined in Figure 1A). Sticklebacks from two independently derived freshwater populations [Fishtrap Creek (FTC) and Bear Paw Lake (BEPA)] were crossed to fish from a single marine population (LITC). These two F2 crosses are hereafter called the FTC and BEPA crosses. F2 fish (n = 358 and 361 in the FTC and BEPA crosses, respectively) were sequenced with GBS, multiplexing up to 384 samples in a single Illumina lane (Table 1, Figure S1, Table S2). SNPs were phased using grandparent resequencing and filtered for those that had proper allele ratios and coverage levels, resulting in 131,091 and 87,419 high-quality segregating SNPs in the FTC and BEPA crosses (Figure 1, A and B). To generate high-quality genotypes, we binned together multiple low-coverage SNPs into high-coverage binned markers (referred to as markers in this study) using bins of equal size of, at most, 500 kb (Figure 1C). Linkage maps were made with 1001 and 978 markers in the FTC and BEPA crosses (Figure 1A, Figure S3, Figure S4, File S1, File S2, File S3). These maps had missing genotype rates of 1.9% (FTC) and 1.8% (BEPA). The sex of each F2 fish was determined from sequencing coverage levels of the sex chromosome (Figure 1D).

### Improvements to stickleback genome assembly

**Figure 2:**
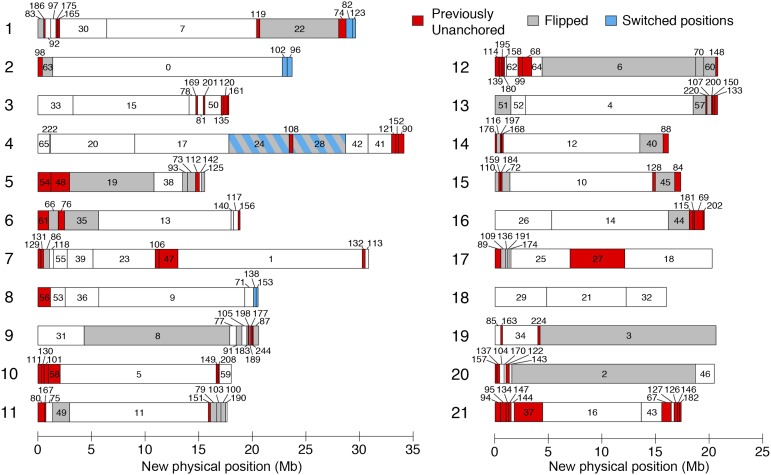
A revised map of stickleback scaffold order and orientation. Consensus scaffold map from the two crosses. Chromosomes are numbered on the left, and scaffolds are numbered, with previously unanchored scaffolds colored red, previously anchored scaffolds whose orientation has been flipped colored gray, and scaffolds that have switched positions colored blue. See File S4 for coordinates of scaffold locations. Figure style adapted from Roesti *et al.* 2013.

The stickleback reference genome assembly (Jones *et al.* 2012) contains 113 anchored and 1822 unanchored scaffolds, which comprise 86.8% and 13.2% of the genome assembly, respectively. In both linkage maps, all previously anchored scaffolds mapped to their originally assigned chromosome. In addition, in all cases where a scaffold mapped to a chromosome in both crosses, the scaffold mapped to the same chromosome. Combining the two linkage maps, we generated a consensus scaffold map containing 186 scaffolds, which differed from the genome assembly in 153 places. These differences consisted of 78 previously unanchored scaffolds that were newly anchored in the genome (comprising 36.1 Mb), 40 inversions of previously anchored scaffolds (113.3 Mb), and 4 rearrangements of previously anchored scaffolds (12.8 Mb) (Figure 2, File S4). In the consensus scaffold map, the 186 total scaffolds comprised 94.6% of the total assembly sequence and included the largest 124 scaffolds. Based on the linkage map positions of markers within each scaffold, an orientation was determined for 166 of the 186 scaffolds (436.2 Mb). The anchored scaffolds in the consensus scaffold map contained 9% more bases and Ensembl-predicted genes (Jones *et al.* 2012) than in the original assembly (Table 2). The revised genome assembly sequence and adjusted positions of Ensembl-predicted gene locations are available in File S5, File S6, and File S7.

**Table 2:** Summary of improved genome assembly

	Scaffolds		Length (Mb)		Genes	
Chr	Original	Revised	Original	Revised	Original	Revised
1	8	13	28.18	29.63	1262	1328
2	4	5	23.29	23.7	861	907
3	5	10	16.79	17.8	934	1004
4	8	12	32.63	34.14	1329	1410
5	6	9	12.25	15.56	733	861
6	5	8	17.08	18.85	721	760
7	7	12	27.93	30.84	1320	1481
8	6	7	19.36	20.53	885	924
9	9	11	20.24	20.58	1012	1016
10	2	8	15.66	18.03	816	931
11	7	10	16.7	17.64	1060	1108
12	6	13	18.4	20.76	1007	1138
13	6	9	20.08	20.74	971	1014
14	3	7	15.24	16.17	739	792
15	5	8	16.19	17.32	778	823
16	3	7	18.11	19.52	803	864
17	6	8	14.6	20.25	702	1064
18	4	3	16.28	15.99	764	739
19	3	5	20.24	20.61	1046	1086
20	5	8	19.73	20.45	934	990
21	5	13	11.71	17.35	464	614
Total	113	186	400.7	436.45	19,141	20,854

A comparison of the original genome assembly and the revised scaffold order presented in this study. The number of scaffolds, the physical size in megabases (Mb), and the number of Ensembl-predicted genes (Jones *et al.* 2012) are compared.

We developed a second, more sensitive method to map unanchored scaffolds by examining the correlation of read counts for every pair of markers (see Supplemental Methods in File S8). With this read correlation method, 538 scaffolds (96.9% of total assembly sequence), including 352 scaffolds not mapped by the first method, mapped to within approximately 5 cM of a marker in the consensus scaffold map (Table S3). Compared to the linkage map-based assembly, an additional 10.8 Mb and 490 Ensembl-predicted genes were linked to a chromosome.

**Figure 3:**
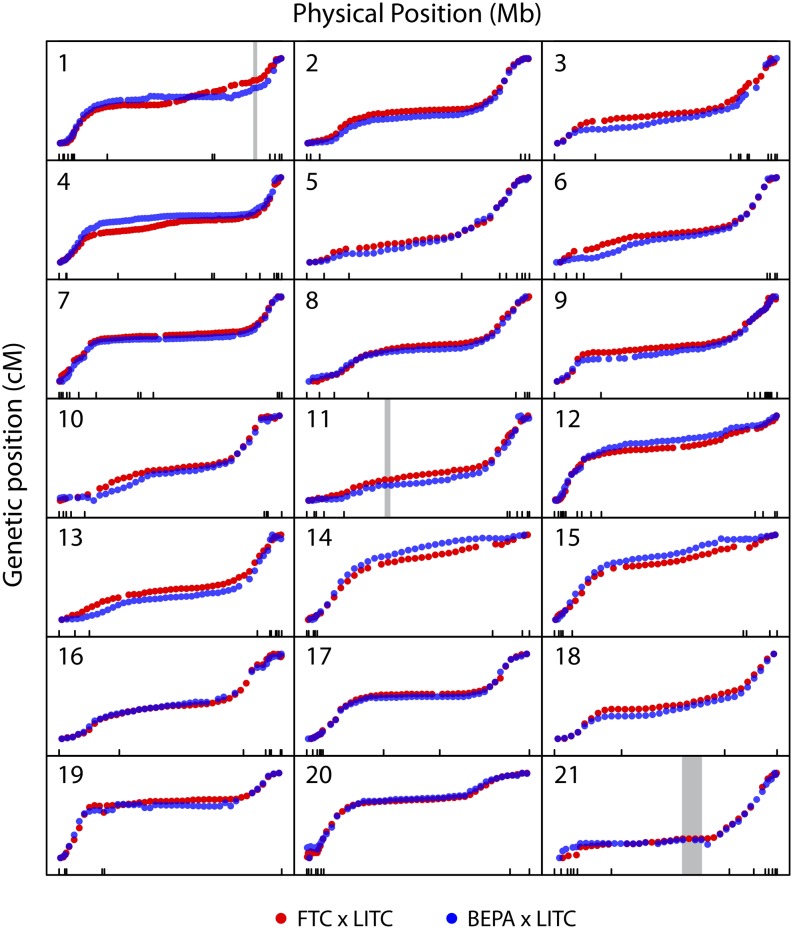
Similar genome-wide recombination patterns in both crosses. Plots of genetic *vs.* physical position for each chromosome. Plots are scaled to have constant width and height for each chromosome. Markers from the FTC and BEPA crosses are plotted in blue and red, respectively. Physical position is according to the revised scaffold map, and tick marks along the x-axis indicate scaffold boundaries. Most chromosomes have highly similar regions of high and low recombination rates between the two crosses. The positions of the three marine/freshwater inversions on chromosomes 1, 11, and 21 reported in Jones *et al.* (2012) are indicated with gray rectangles. For a closer zoom-in of these inversions see Figure S6, which shows that no recombination events were detected within the three inversions.

To determine whether large-scale genomic rearrangements or patterns of recombination rates differ between freshwater populations, we examined the genome-wide patterns of recombination. The two crosses did not indicate any large-scale differences in genomic structure and had strikingly similar patterns of recombination across the genome, with similar regions of high and low recombination (Figure 3). The BEPA cross had an elevated overall recombination rate relative to the FTC cross, with a total map size of 1570 cM and 1963 cM in the FTC and BEPA crosses, respectively. This difference was due to an elevated rate of recombination throughout the genome (Figure S5). Consistent with a previous study (Roesti *et al.* 2013), most chromosomes appeared to have suppressed recombination in the middle, with ends of chromosomes having higher rates of recombination (Figure 3). The pattern of recombination within each chromosome correlated partially with previously described chromosome morphologies (Urton *et al.* 2011). For example, as predicted, recombination rates were high on both ends of metacentric chromosome 7 and recombination occurred mostly on one end of telocentric chromosome 15. However, some chromosomes did not match predictions (*e.g.*, recombination occurred mostly on one end of metacentric chromosomes 14 and 21). As expected, in both crosses recombination was completely suppressed in three previously described (Jones *et al.* 2012) marine/freshwater inversions on chromosomes 1, 11, and 21 (Figure S6).

### QTL mapping of lateral plate reduction

**Figure 4:**
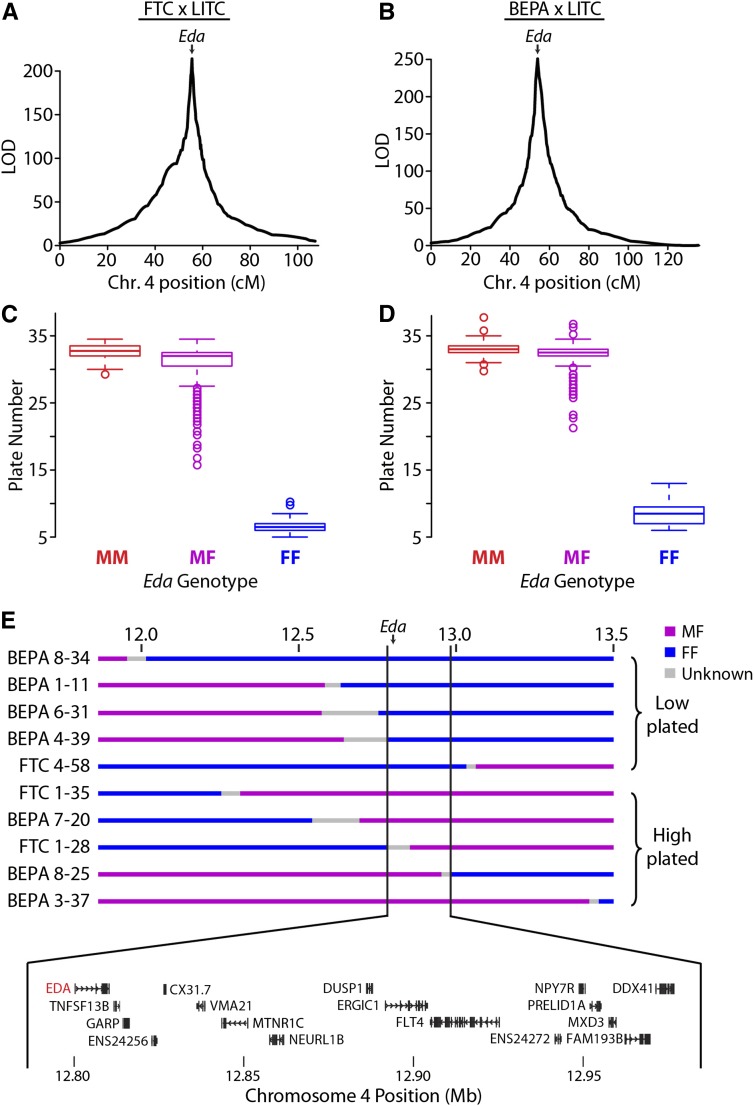
Lateral plate reduction is controlled by a near-Mendelian locus containing *Eda*. QTL mapping of plate number in the FTC (A) and BEPA (B) crosses. A main large-effect QTL was found in both crosses on chromosome 4 with a single peak at *Ectodysplasin (Eda)*. cM, centimorgan. Boxplots showing association between *Eda* genotype and lateral plate number in the FTC (C) and BEPA (D) crosses. M, marine; F, freshwater. The QTL is recessive, with all MF and MM fish having more than 15 plates and all FF fish having fewer than 15 plates. (E) Fine-mapping the chromosome 4 lateral plate QTL with MF/FF recombinants in both crosses. Genotype at a 199.8-kb interval perfectly correlates with whether plate number is low (<15 plates per side) or high (>15 plates). This interval contains the coding regions of 17 Ensembl-predicted genes, including *Eda* and recently identified intergenic regulatory mutations of *Eda* (O’Brown *et al.* 2015). ENS24256 and ENS24272 refer to ENSGACT00000024256 and ENSGACT00000024272, respectively.

Like most freshwater populations, freshwater FTC and BEPA fish have evolved reduced lateral plates (Cresko *et al.* 2004; Hagen and Gilbertson 1972). Plate reduction is typically controlled by a large-effect QTL on chromosome 4 in both Pacific Northwest and Alaskan freshwater populations, including BEPA (Colosimo *et al.* 2004; Cresko *et al.* 2004). This QTL has been shown to be a regulatory haplotype of the *Ectodysplasin* (*Eda*) gene (Colosimo *et al.* 2005; O’Brown *et al.* 2015). Lab-reared FTC and BEPA fish were both low-plated (Figure S7A). As a positive control to validate the GBS linkage maps, we mapped lateral plate number QTL in both crosses. As expected, in both crosses a near-Mendelian QTL on chromosome 4 (percent variance explained of 97.8% and 95.7% in the FTC and BEPA crosses, respectively) controlled lateral plate number (Figure 4, A and B; Table 3). The chromosome 4 QTL was largely recessive in each cross; all marine homozygotes and heterozygotes had more than 15 plates and all freshwater homozygotes had fewer than 15 plates (Figure 4, C and D). In contrast with Berner *et al.* (2014), we did not find a double QTL peak on chromosome 4 in either cross (Figure 4, A and B), consistent with a single underlying genetic locus.

**Table 3:** QTL identified in this study

						1.5 LOD Interval			Trait Mean ± SE		
Trait	Cross	Chr	n	LOD	PVE	Left	Peak	Right	MM	MF	FF
Plate #	FTC	4	356	214.1	97.8	17_7	17_8	17_10	32.7 ± 0.3	30.7 ± 0.2	6.7 ± 0.3
Plate #	BEPA	4	359	244.9	95.7	17_8	17_9	17_10	33 ± 0.3	32.1 ± 0.2	8.8 ± 0.2
Plate # (*Eda* hets)	FTC	7	184	5.7	13.3	39_5	23_7	1_23	32 ± 0.5	31.1 ± 0.3	28.4 ± 0.5
Lat. GR length	FTC	10	246	3.8	6.3	5_19	5_24	5_29	1025 ± 13	1032 ± 9	973 ± 14
Lat. GR length	FTC	20	246	4.6	9.3	2_6	2_3	46_3	1039 ± 13	1032 ± 9	967 ± 12
Mid. GR length	FTC	1	251	5.5	7.4	30_2	7_10	22_5	968 ± 13	955 ± 9	903 ± 12
Mid. GR length	FTC	4	251	4.8	7.6	20_8	17_8	42_4	987 ± 12	938 ± 9	902 ± 14
Mid. GR length	FTC	11	251	4.8	7.5	11_11	11_18	11_23	984 ± 12	943 ± 8	893 ± 14
Lat. GR length	BEPA	16	298	4.5	6.3	44_4	44_3	115_1	1152 ± 14	1130 ± 10	1078 ± 13
Lat. GR length	BEPA	20	298	4.6	6.5	2_31	2_9	2_7	1078 ± 13	1139 ± 10	1139 ± 15
Mid. GR length	BEPA	16	300	6.2	9.1	44_4	44_3	115_2	1054 ± 14	1031 ± 9	955 ± 14
Med. GR length	BEPA	16	301	4.3	6.0	44_4	44_3	115_2	700 ± 13	676 ± 9	620 ± 13
Med. GR length	BEPA	19	301	4.4	6.1	3_7	3_2	3_1	627 ± 13	661 ± 10	706 ± 12

**Figure 5:**
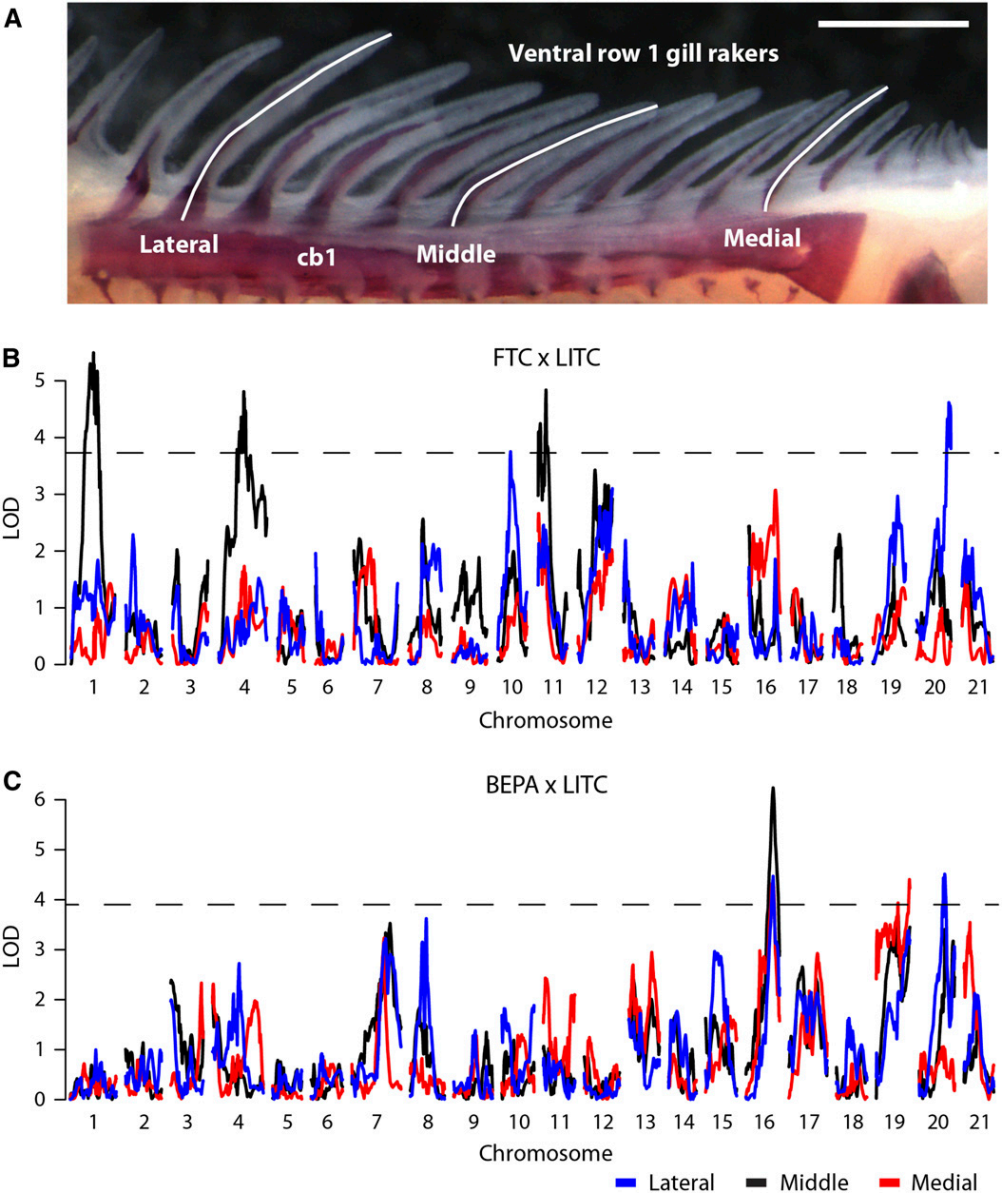
Genetic mapping of gill raker length reduction. (A) Lengths of three ventral row 1 gill rakers were measured at lateral, middle, and medial positions. cb1, ceratobranchial 1. Scale bar = 500 um. Manhattan plots of QTL mapping of gill raker length in the FTC cross (B) and the BEPA cross (C). Three gill raker lengths were mapped: lateral (blue), middle (black), and medial (red). LOD is shown as a function of genetic position. The significance thresholds (α = 0.05) are shown with a dotted line.

Lat., lateral; mid., middle; med., medial (see Figure 5A); LOD, log of the odds; PVE, percent variance explained; M, marine allele; F, freshwater allele. The intervals for the chromosome 4 plate number QTL were further defined through fine mapping of recombinant breakpoints (Figure 4E). The FTC chromosome 7 plate number QTL was identified by mapping plate number in *Eda* heterozygotes. PVE refers to residual variance in *Eda* heterozygotes, not total plate number variance. LOD significance thresholds (α = 0.05) were 3.85/3.92 for plate number and 3.73/3.90 for gill raker length in the FTC/BEPA crosses, and 4.41 for plate number in *Eda* heterozygotes in the FTC cross. See Figure 4, A and B, Figure 5, B and C, and Figure S7B for lodplots of the QTL. Gill raker lengths are in microns.

To test the resolution of the linkage maps, we used a Hidden Markov Model on the raw allele counts for each SNP to fine-map recombination breakpoints (Figure S6A). This method enabled us to fine-map recombinant breakpoints to a median resolution of 89 kb (Figure S6B). With the fine-mapped recombination breakpoints, we identified 10 heterozygous/homozygous freshwater recombinant animals in the two crosses that recombined within a 1-Mb interval surrounding *Eda*. These recombinant animals defined a 199.8-kb interval that perfectly correlates with plate number in both crosses (Figure 4, C–E). This interval contains 17 Ensembl-predicted genes, including *Eda*, as well as an intergenic SNP recently shown to affect a lateral plate enhancer (O’Brown *et al.* 2015). No additional QTL were detected upon conditioning on *Eda* genotype in a single model (data not shown). Because *Eda* heterozygotes had the most variance of any genotypic class (Figure 4, C and D), we mapped plate number in *Eda* heterozygotes (as in Colosimo *et al.* 2004) and detected one modifier QTL on chromosome 7 in the FTC cross but no significant modifier QTL in the BEPA cross (Figure S7B, Table 3).

### QTL mapping of gill raker length

We previously discovered a strikingly high degree of modularity of skeletal evolution in sticklebacks, consistently observed across a variety of axial and craniofacial skeletal traits (Miller *et al.* 2014). To test the hypothesis that gill raker length is also under modular genetic control, we examined gill rakers at three positions, located at lateral, middle, and medial points of the anterior-most ceratobranchial bone (Figure 5A). We observed a modular reduction of gill raker length in lab-reared FTC and BEPA fish, with strongest length reductions in the lateral and middle domains (Figure S8). QTL controlling gill raker length were detected on chromosomes 1, 4, 10, 11, and 20 in the FTC cross and chromosomes 16, 19, and 20 in the BEPA cross (Figure 5, B and C; Table 3). The peak marker on chromosome 4 in the FTC cross was 17_8, a bin containing *Eda*. While one QTL (chromosome 16 in the BEPA cross) had effects on lateral, middle, and medial gill raker lengths, most QTL were surprisingly modular, with significant effects on only one gill raker length (Table 3). Most (six of eight) of the gill raker length QTL were concordant with the direction of evolutionary change (freshwater allele yielding shorter gill rakers), consistent with gill raker length being under strong natural selection. QTL on chromosome 20 controlling lateral gill raker length were detected in both crosses, but had nonoverlapping 1.5 LOD intervals. Overall, no QTL with overlapping 1.5 LOD intervals were observed in both crosses. Thus, unlike lateral plate reduction, the convergent evolution of gill raker length reduction has occurred via distinct genetic bases in these two freshwater populations.

## Discussion

### High-quality linkage maps from binned GBS

The power of next-generation sequencing has revolutionized high-throughput genotyping, beginning in 2008 with the RAD-seq approach (Baird *et al.* 2008). RAD-seq was rapidly applied to a variety of model and nonmodel organisms (Rowe *et al.* 2011; Narum *et al.* 2013). An extension of RAD-seq, the simpler and cheaper GBS method was published in 2011 (Elshire *et al.* 2011). In fish, RAD-seq or GBS has been used to build genome-wide linkage maps in stickleback (Roesti *et al.* 2013; this study), salmon (Gonen *et al.* 2014; Limborg *et al.* 2014), and Mexican tetra (Carlson *et al.* 2015), in each case successfully building a map with approximately a marker per centimorgan. Differences in these linkage maps appear largely attributable to the details of the cross design (*e.g.*, number of F2 fish genotyped), sequencing depth (*e.g.*, number of lanes sequenced and whether single or paired end reads were sequenced), and/or genome assembly used to align reads (*e.g.*, size of genome). For example, our threespine stickleback maps presented here are larger in total genetic distance than those in the work by Roesti *et al.* (2013); however, we analyzed more than twice as many F2 fish, sequenced paired end reads *vs.* single end reads, and generated more total sequences than in this previous study. In contrast, our stickleback maps are smaller than the total genetic length of a recently published Mexican tetra map, likely in part due to Mexican tetra having twice as large a genome as stickleback. One methodological difference between our maps and these other fish linkage maps is that we used a binned approach, binning SNPs to generate genetic markers, similar to that successfully used in corn (Li *et al.* 2015; Chen *et al.* 2014).

This study utilized a binned GBS approach to generate high-quality genotypes. First, a large number of SNPs (approximately 100,000 per cross) were sequenced to a low level of sequencing coverage (approximately 1.5× per sample). Then, multiple SNPs were binned together to form approximately 1000 high-coverage (approximately 150×) markers. This approach contrasts with other reduced representation approaches (Baird *et al.* 2008; Elshire *et al.* 2011; Peterson *et al.* 2012; but see Andolfatto *et al.* 2011) that target a smaller number of SNPs (typically 1000–5000) at high coverage (>20×). The tradeoff of coverage *vs.* SNP number can easily be controlled through the choice of restriction enzymes as well as the degree of size selection of the library (Peterson *et al.* 2012). This study used ApeKI, which cuts a 5-bp restriction site that occurs frequently in the genome, and no library size selection to target a large number of SNPs. Individual SNPs can be biased toward one allele, have mapping or genotyping errors, and have variable coverage levels. Thus, binning a large number of low-coverage SNPs together resulted in a robust and reliable set of genotypes. The binning approach in this study also enabled the use of the same bins of markers for direct comparison between crosses. Several quality-control steps appeared to be crucial to generate high-quality linkage maps, including dropping SNPs and markers that deviated from expected allele ratios, dropping low-coverage samples and markers, and using a separate computational pipeline to generate sex chromosome genotypes.

It is unlikely that the order and orientation of scaffolds presented in this study are completely correct or universal to all sticklebacks. However, there are several reasons to believe that most of the revised scaffold orders and orientations in this study are correct and typical stickleback features, rather than individual polymorphisms for genomic rearrangements. First, the majority of the scaffold orders and orientations were supported by multiple markers from both crosses, which derived from freshwater fish from two independently derived populations, including one fish from the same population as the original reference genome (BEPA). Second, the 31 changes to the stickleback genome assembly identified in a cross of (geographically distant) European sticklebacks by Roesti *et al.* 2013 were all detected by this study. Third, two of the scaffold changes identified in this study (on chromosomes 19 and 20) are consistent with previous cytogenetic evidence (Urton *et al.* 2011). The expanded set of assembly changes identified in this study (78 newly anchored scaffolds, 40 reoriented scaffolds, and 4 scaffold rearrangements) should further aid efforts to understand the evolutionary dynamics of the stickleback genome and discover the genes underlying adaptive phenotypes in sticklebacks.

We observed little difference in the two maps whose SNPs were phased by sequencing the cross grandparents to high (60×, FTC) *vs.* low (6×, BEPA) coverage. We suspect that sequencing grandparents with GBS, as opposed to full genome sequencing, could further reduce costs. Several cost-saving factors, including halving reagent volumes during library creation and barcoding 384 samples together, did not result in a significant decrease in genotyping quality. In library 7, we used 96 barcoded adapters and 4 index primers to multiplex 384 samples in a single lane of Illumina sequencing. The high average marker coverage (approximately 50×) and high genotyping success rate with 384 barcoded samples (<3% genotyping fail rate, see Table 1) suggest that more samples could be multiplexed together. For instance, 48 barcoded adapters and 16 index primers could allow barcoding of 768 samples while requiring fewer unique primers.

### Genetic mapping of lateral plate reduction

QTL mapping of armor and trophic traits demonstrated the power of dense GBS-generated genome-wide linkage maps to detect QTL of large and small effect, as well as tested several hypotheses about the genetic basis of these adaptive skeletal changes. As expected from previous genetic studies of lateral plate number (Colosimo *et al.* 2004; Cresko *et al.* 2004), a large-effect QTL on chromosome 4 controlled plate number in both crosses. In contrast to a previous study that reported multiple QTL peaks on chromosome 4 for lateral plate number, perhaps suggesting multiple underlying chromosome 4 loci (Berner *et al.* 2014), in this study the chromosome 4 QTL in each cross had a clear single peak at *Eda*. A 17-gene minimal genomic interval included *Eda* and a recently identified intergenic lateral plate enhancer with a polymorphic SNP that affects enhancer activity (O’Brown *et al.* 2015). Previous mapping of plate modifier QTL in *Eda* heterozygotes identified three plate number modifier QTL in a similarly sized cross with the Paxton benthic (PAXB) population (Colosimo *et al.* 2004). With an identical mapping approach, we detected fewer modifier QTL (1 and 0 in the FTC and BEPA crosses, respectively). This difference in genetic architecture might be due to differences in the extent of plate reduction in the freshwater populations used in the studies [mean of 0.3 plates in PAXB (McPhail 1992) *vs.* 4.9 in FTC and 4.4 in BEPA]. Intriguingly, the chromosome 7 modifier QTL detected in the FTC cross overlaps one of the three previously detected plate modifier QTL (Colosimo *et al.* 2004). This chromosome 7 modifier QTL might be reused along with *Eda* in multiple freshwater populations to reduce plate number.

### Genetic mapping of gill raker length reduction

We also identified eight new QTL controlling the classic adaptive trait of gill raker length (Schluter 2000). Motivated by our previous finding of pervasive modularity in the evolution of serially homologous axial and craniofacial skeletal elements (Miller *et al.* 2014), we hypothesized that gill raker length might also be genetically controlled in a modular fashion. Gill raker lengths measured at different mediolateral locations had surprisingly different genetic architectures, indicating complex modularity of this trait. Therefore, gill raker lengths at different positions might not be directly comparable in ecological studies. This modularity might reflect differences in retaining different types of prey with gill rakers of different lengths along the mediolateral axis. Developmental timing might contribute to this genetic modularity, as gill rakers form during embryonic development in a wave from lateral to medial (Glazer *et al.* 2014). Intriguingly, the chromosome 4 gill raker length QTL in the FTC cross has a peak marker bin that contains *Eda*. The EDA pathway, in addition to its role in plate development, is intimately involved in gill raker development in zebrafish and sticklebacks (Glazer *et al.* 2014; Harris *et al.* 2008). However, in the BEPA cross, a chromosome 4 plate number QTL, but not a gill raker length QTL, was detected. Therefore, if *Eda* is contributing to the FTC gill raker length QTL, there is likely different linked regulatory variation of *Eda* in FTC compared to BEPA.

We previously identified an enrichment of skeletal QTL on chromosomes 4, 20, and 21 (Miller *et al.* 2014), and our findings here add gill raker length as yet another skeletal trait controlled by two of these three trait clusters, as gill raker length mapped to chromosome 20 in both crosses, and chromosome 4 in the FTC cross. Linked chromosome 4 and 20 alleles promoting reduction of gill raker length (this study) and gill raker number (Glazer *et al.* 2014; Miller *et al.* 2014) might promote co-evolution of these phenotypes in freshwater environments. In sticklebacks, a predictable, shared genetic basis has been found to underlie the convergent evolution of several evolved phenotypes (Chan *et al.* 2010; Colosimo *et al.* 2004, 2005; Glazer *et al.* 2014; Miller *et al.* 2007). In contrast to these studies, we detected no overlapping gill raker length QTL in the two crosses. In addition, none of the gill raker length QTL in this study overlap two previously reported QTL from a European lake × stream cross (Berner *et al.* 2014). Thus, unlike several other stickleback phenotypes, different loci appear to underlie the convergent evolution of gill raker length in different populations.

## Conclusions

This work used a binned GBS approach to build dense linkage maps of sticklebacks, which were used for genome assembly improvement and QTL mapping of two ecologically important traits. The revised genome assembly provides a more accurate understanding of the structure of the stickleback genome, which should aid efforts to map genes controlling stickleback phenotypes and understand genomic dynamics during stickleback evolution. The genetic mapping of distinct QTL controlling gill raker length in two crosses illustrates that, in contrast to several prominent cases in sticklebacks (Chan *et al.* 2010; Colosimo *et al.* 2005; Miller *et al.* 2007), a nonparallel genetic basis is sometimes used in cases of repeated phenotypic evolution.

## Supplementary Material

Supporting Information

Corrigendum
