# Supplementary material for: Genome Assembly Improvement and Mapping Convergently Evolved Skeletal Traits in Sticklebacks with Genotyping-by-Sequencing
Source: G3 (Bethesda). 2015 Jun 3;5(7):1463–72. doi: 10.1534/g3.115.017905 (PMC4502380; doi:10.1534/g3.115.017905)
Supplement: Supporting Information [file supp_5_7_1463__index.html]

Genome Assembly Improvement and Mapping Convergently Evolved Skeletal Traits in Sticklebacks with Genotyping-by-Sequencing — Supporting Information 

# Genome Assembly Improvement and Mapping Convergently Evolved Skeletal Traits in Sticklebacks with Genotyping-by-Sequencing

## Supporting Information for Glazer *et al.*, 2015

**Files in this Data Supplement:**

- Supporting Information - Figures S1-S8, Files S1-S8, and Tables S1-S3 (PDF, 610 KB)
- Figure S1 - Diagram of library making method. (PDF, 276 KB)
- Figure S2 - Genotype calling from marine and freshwater read counts. (PDF, 159 KB)
- Figure S3 - Linkage map from FTC x LITC cross. (PDF, 168 KB)
- Figure S4 - Linkage map from BEPA x LITC cross. (PDF, 169 KB)
- Figure S5 - High correlation of recombination rates. (PDF, 158 KB)
- Figure S6 - Fine-mapping recombinant breakpoints with a Hidden Markov Model. (PDF, 186 KB)
- Figure S7 - QTL mapping of lateral plate modifiers. (PDF, 203 KB)
- Figure S8 - Convergent evolution of freshwater gill raker length reduction. (PDF, 150 KB)
- File S5 - Fasta file containing revised genome assembly based on consensus scaffold order and orientation as described in File S4. Data available from the Dryad Digital Repository: http://dx.doi.org/10.5061/dryad.q018v
- File S6 - Repeat masked fasta file containing revised genome assembly based on consensus scaffold order and orientation as described in File S4. Repeat masked fasta file is based off the repeat masked version of the original genome assembly, which was masked with RepeatMasker. Data available from the Dryad Digital Repository: http://dx.doi.org/10.5061/dryad.q018v
- File S7 - Revised .gtf file of Ensembl gene predictions. Coordinates of gene predictions were converted to the revised assembly coordinates. All Ensembl-predicted genes were included, except ENSGACT00000019430, which spans two scaffolds (11 and 79) that are not adjacent in the revised genome assembly. Data available from the Dryad Digital Repository: http://dx.doi.org/10.5061/dryad.q018v
- File S8 - Supplemental Methods (PDF, 155 KB)
- Table S1 - Primers used in this study. (PDF, 147 KB)
- Table S2 - Cross summary statistics. (PDF, 138 KB)
- Table S3 - Anchored scaffolds. (PDF, 195 KB)
- File S1 - FTC cross summary information. (.xlsx, 1 MB)
- File S2 - BEPA cross summary information. (.xlsx, 1 MB)
- File S3 - Summary of bins in genome assembly. (.xlsx, 238 KB)
- File S4 - Revised consensus scaffold order (.xlsx, 169 KB).
