# Supplementary material for: Genome Assembly Improvement and Mapping Convergently Evolved Skeletal Traits in Sticklebacks with Genotyping-by-Sequencing
Source: G3 (Bethesda). 2015 Jun 3;5(7):1463–72. doi: 10.1534/g3.115.017905 (PMC4502380; doi:10.1534/g3.115.017905)
Supplement: Supporting Information [file supp_g3.115.017905_017905SI.pdf]

G3·Genes | Genomes | Genetics

Genome assembly improvement and mapping convergently evolved skeletal traits in sticklebacks with Genotyping-by-Sequencing

Andrew M. Glazer, Emily E. Killingbeck, Therese Mitros, Daniel S. Rokhsar, and Craig T. Miller[1]

Molecular and Cell Biology Department, University of California-Berkeley,
Berkeley, CA 94720, USA
[1]Corresponding author: Craig T. Miller. Molecular and Cell Biology Department, University of California-Berkeley, Berkeley, CA 94720, USA. Phone: (510)-642-7840. Email: ctmiller@berkeley.edu.

DOI: 10.1534/g3.115.017905

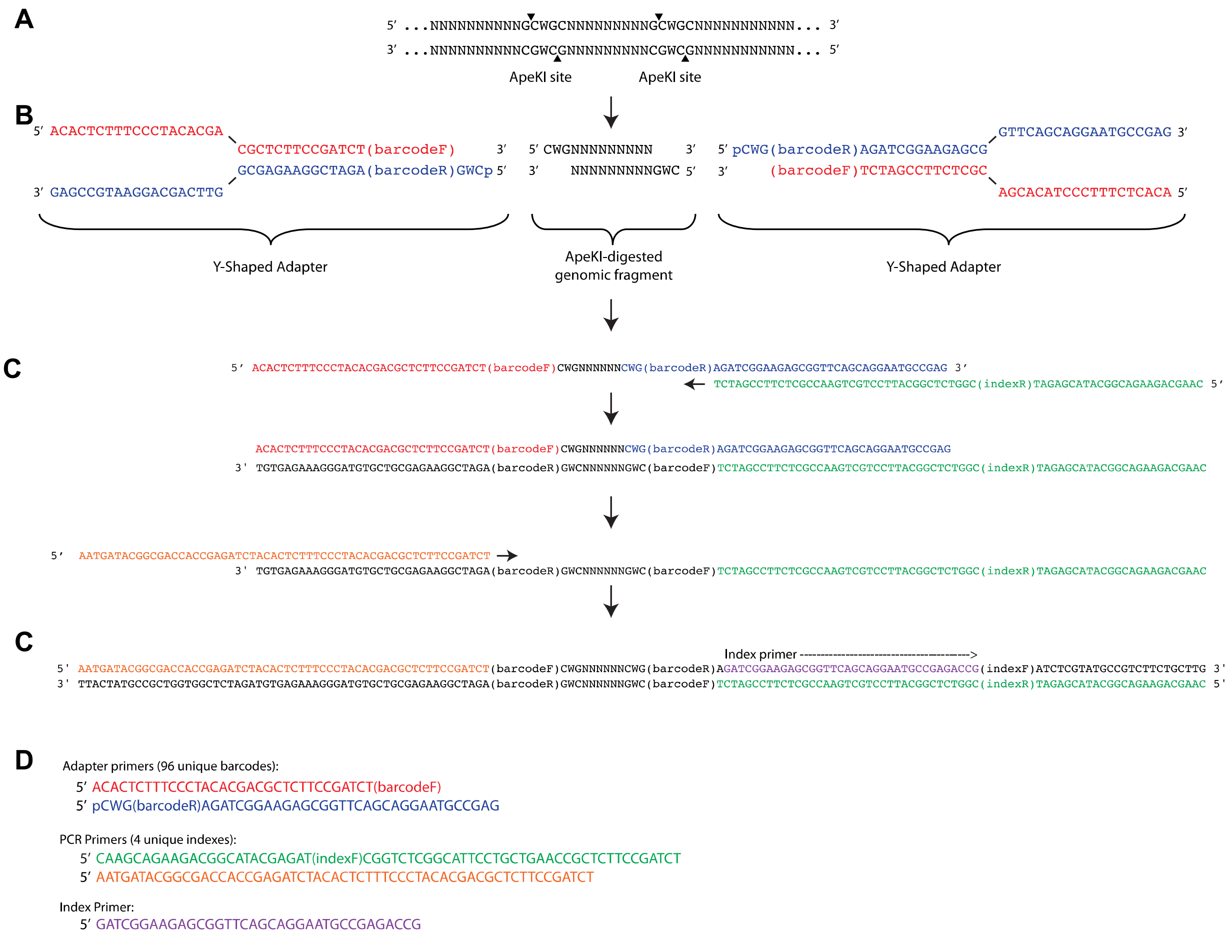

Figure S1 Diagram of library making method.
(A) Genomic DNA was digested with ApeKI, creating CWG overhangs. (B) Pairs of adapter primers were annealed together to create a "Y-shaped" primer, with partial base-pairing at one end. These adapters were ligated to both sides of the digested genomic DNA. 96 unique adapters were used, containing different internal barcode sequences ("barcodeF" and "barcodeR" together constitute the double-stranded barcode). (C) PCR primers with one of four index barcodes ("index") are used to add sequence that will anneal to the Illumina flowcell. A custom index primer is used during sequencing to determine the index of each fragment, and the first bases of the R1 and R2 reads will contain the barcode. (D) Primers used in this study. See Table S1 for primer sequences.

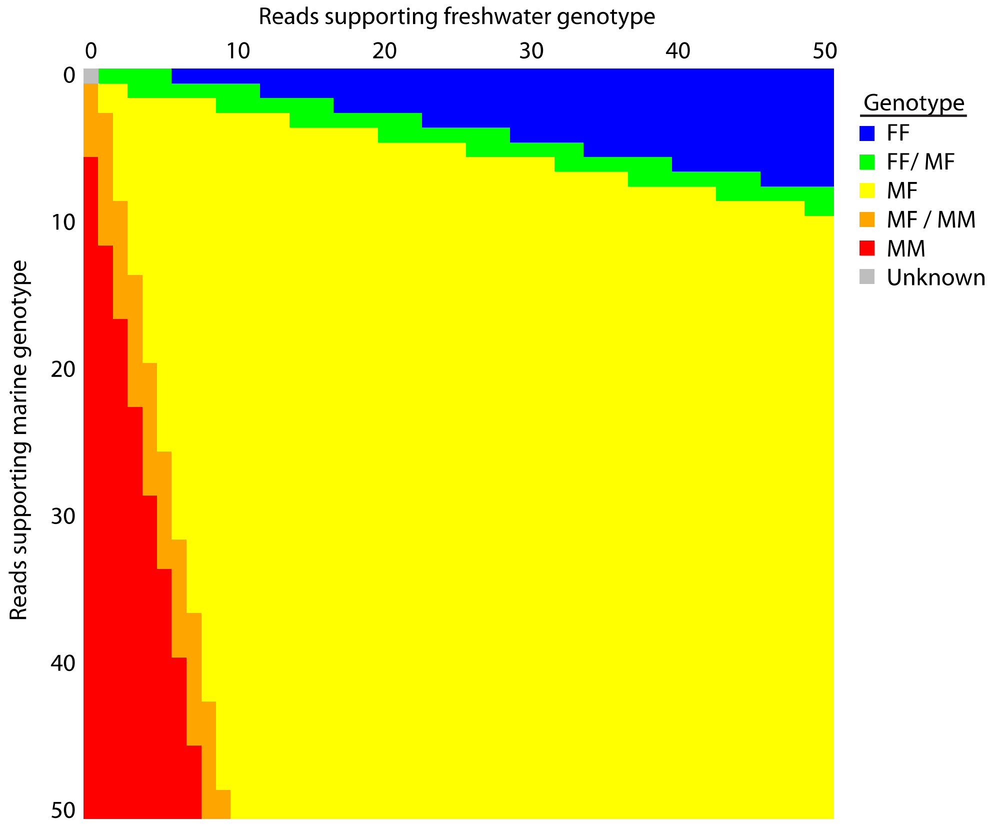

Figure S2 Genotype calling from marine and freshwater read counts
Reads were assumed to be independent and have a 1% error rate. M=marine allele, F=freshwater allele. The FF/MF and MF/MM genotypes indicate that there is a >95% probability that the genotype is not the third possible genotype, but that there is not a >95% certainty for any single genotype call. These genotypes were frequently called for bins that spanned recombination breakpoints.

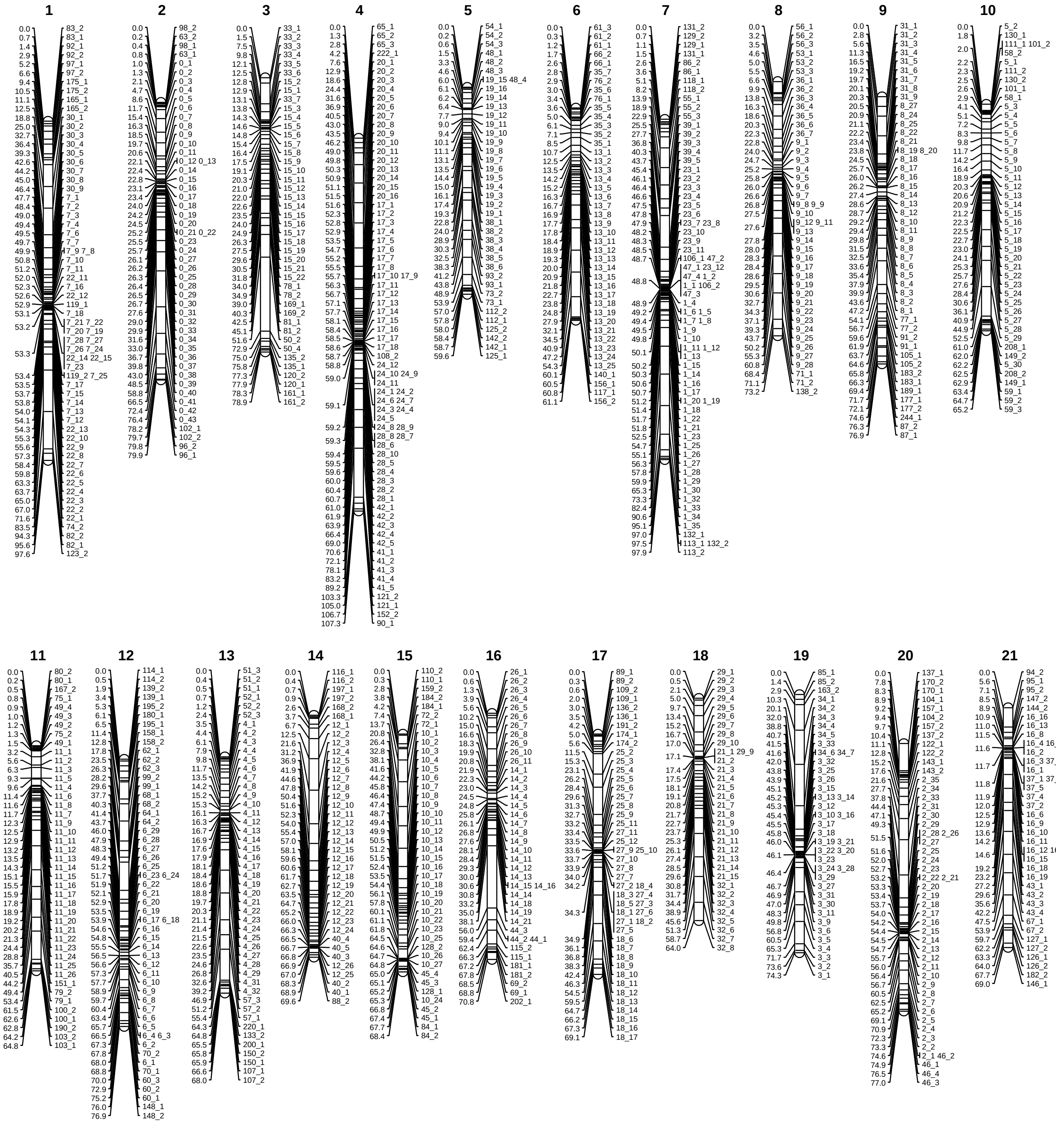

Figure S3 Linkage map from FTC x LITC cross

Diagram of linkage map generated from the FTC x LITC cross. For each chromosome, genetic position of each marker is shown on the left in centiMorgans, and marker name is shown on the right.

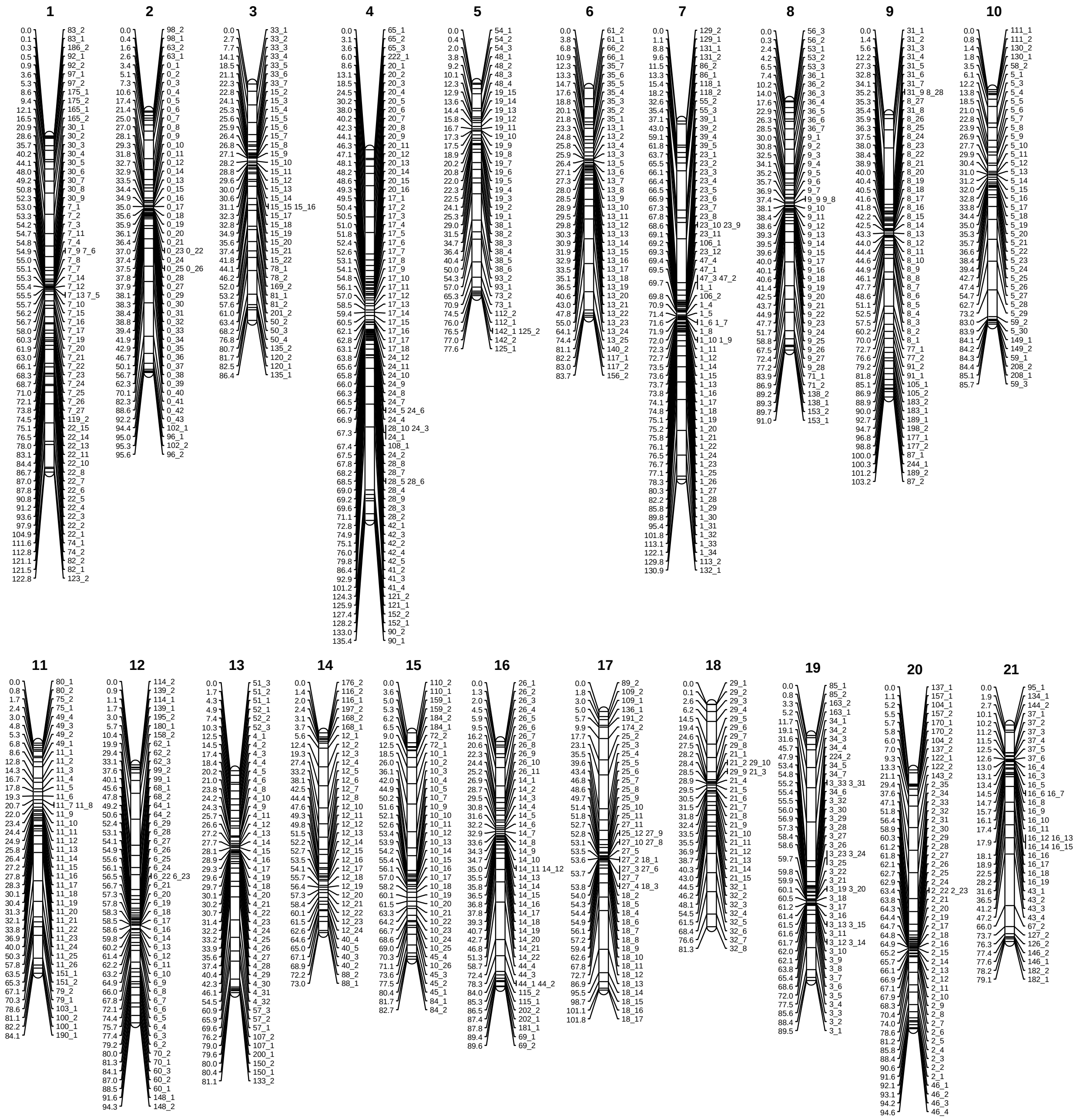

Figure S4 Linkage map from BEPA x LITC cross

Diagram of linkage map generated from the BEPA x LITC cross. For each chromosome, genetic position of each marker is shown on the left in centiMorgans, and marker name is shown on the right.

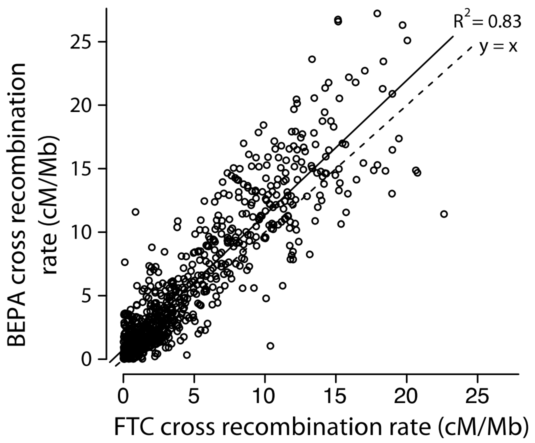

Figure S5 High correlation of recombination rates
Recombination rate in the FTC cross plotted against recombination rate in the BEPA cross. Each point is a marker. Recombination rates were calculated by taking the local slope of the loess-smoothed genetic vs. physical plots shown in Figure 3. Recombination rates are highly correlated (R^2 = 0.83). Solid line: best fit line from a linear regression; dotted line: y=x. Total genetic map size was smaller in the FTC cross (1569.9 cM) compared to the BEPA cross (1963.3 cM). Most points were displaced from the y=x line, indicating a higher recombination rate throughout the genome in the BEPA cross.

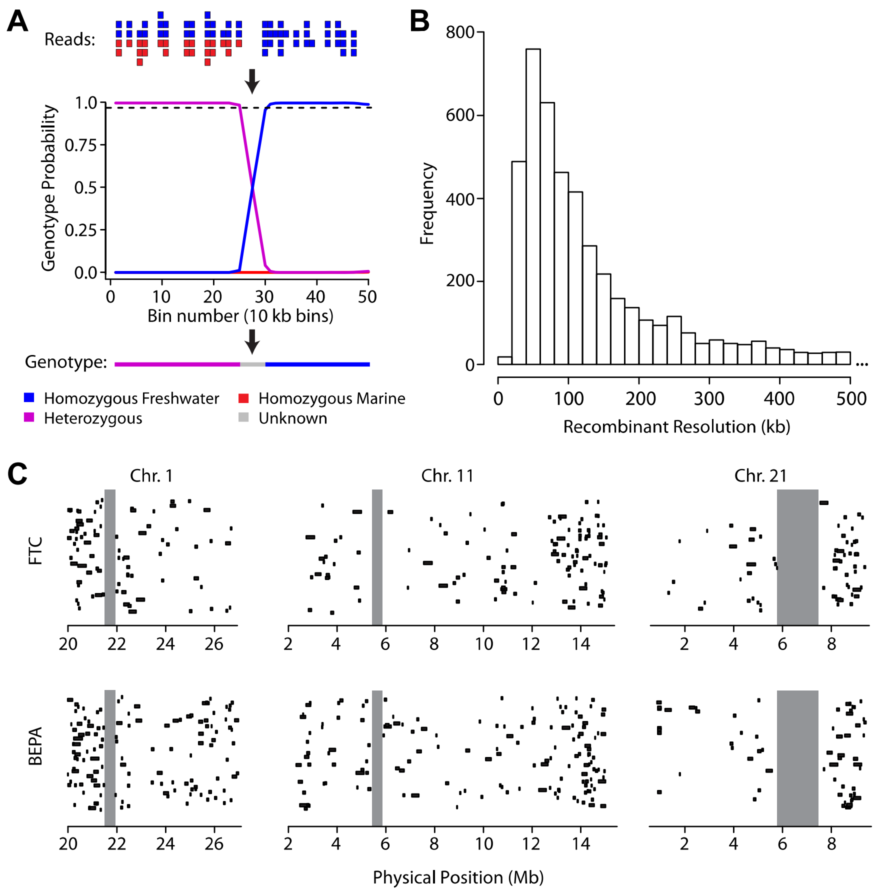

D

Start Probabilites:

M	0.25
H	0.50
F	0.25

Transition Probabilites:

Hidden state (true genotype)		Hidden state (true genotype) M	H	F
	M	0.997	0.002	0.001
	H	0.001	0.998	0.001
	F	0.001	0.002	0.997

Emission Probabilities:

Hidden state (true genotype)		Observed symbols (observed genotypes) M	B	F	X
	M	0.468	0.011	0.001	0.520
	H	0.082	0.317	0.085	0.516
	F	0.001	0.010	0.467	0.522

Figure S6 Fine-mapping recombinant breakpoints with a Hidden Markov Model

(A) Reads were binned into small 10 kilobase (kb) bins. For each bin, the probability of each genotype was determined, and boundaries of the recombination breakpoint were set at the 99% cutoff. (B) Histogram of recombination breakpoint resolution in the FTC x LITC cross. Median resolution was 89 kb. 539 additional recombinants (not shown) had a resolution of greater than 500 kb. (C) Diagram of high-resolution (<350 kb) recombination breakpoints near three marine/freshwater inversions. Each line represents the 99% confidence interval for a recombination breakpoint in a single fish and the grey rectangles represent the previously published marine/freshwater inversion boundaries (Jones et al. 2012). Data is presented for the entire scaffold containing each inversion (scaffold 22 on chromosome 1, scaffold 11 on chromosome 11, and scaffold 16 on chromosome 21). Physical position coordinates from the original assembly are used. No recombination events are observed within these three inversion intervals in each cross. (D) Trained Hidden Markov Model parameters used for fine-mapping recombination breakpoints. SNP genotypes were binned into 10 kb bins. For each fish, each bin was assigned a raw genotype of M (only marine reads), F (only freshwater reads), B (both marine and freshwater reads), or X (no data). These raw genotypes were used as the observed data in a Hidden Markov Model with hidden states M (marine), F (freshwater), or H (heterozygous). The model was trained with the Baum-Welch algorithm on 1 million data points.

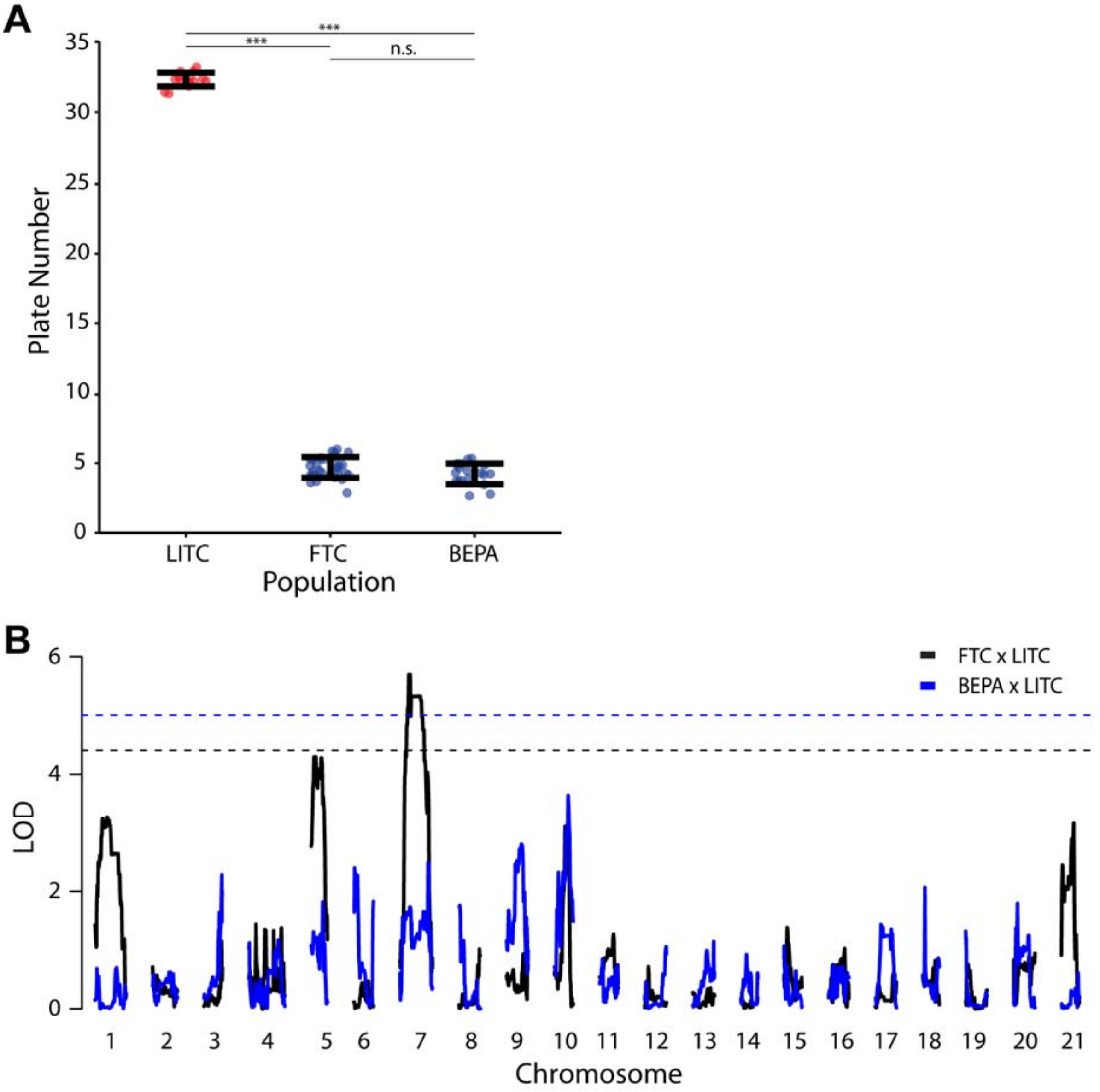

Figure S7 QTL mapping of lateral plate modifiers

(A) Lateral plate reduction in lab-reared FTC and BEPA freshwater fish (blue) compared to LITC marine fish (red). *** indicates $p<10^{-10}$, n.s. = not significant by Tukey's HSD test. Average of left and right side plates is shown. LITC, FTC, and BEPA fish had 32.3+/- 0.5, 4.9+/- 0.7, and 4.4+/- 0.7 plates and sample sizes of 15, 30, and 19, respectively. (B) Manhattan plot of QTL mapping of lateral plate modifiers in the FTC (black) and BEPA (blue) crosses. Mapping was performed in fish heterozygous for *Eda* (n=184/194 in the FTC/BEPA crosses). One QTL was detected in the FTC cross but no significant QTL were detected in the BEPA cross. α = 0.05 significance levels based on 1,000 permutations are shown as dotted lines. LOD is shown as a function of adjusted physical position.

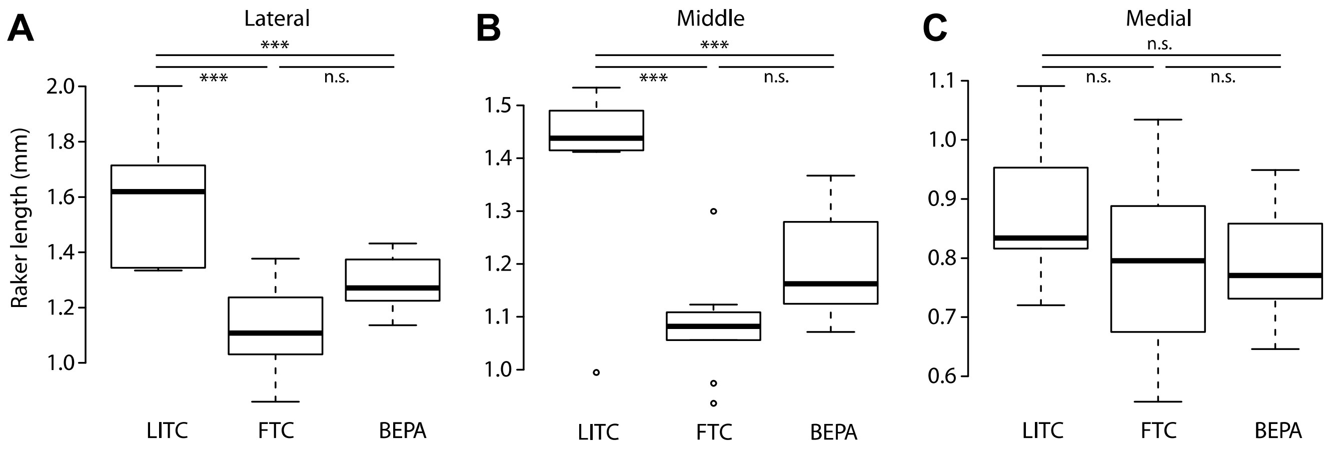

Figure S8 Convergent evolution of freshwater gill raker length reduction

Gill raker lengths were measured in three domains: lateral (A), middle (B), and medial (C) in marine (LITC) and freshwater (FTC and BEPA) lab-reared fish. See Figure 5A for a diagram of gill raker length measurements. Gill raker lengths were back transformed to values expected for a 50 mm standard length fish. *** indicates $p<0.001$, n.s. = not significant by Tukey's HSD test. n=10 per population.

Files S1-S4

Available for download at www.g3journal.org/lookup/suppl/doi:10.1534/g3.115.017905/-/DC1

File S1   FTC cross summary information (.xlsx)

The genetic map, genotypes, sex, raw phenotypes, and size-adjusted phenotypes used for mapping from the FTC cross.

File S2   BEPA cross summary information (.xlsx)

The genetic map, genotypes, sex, raw phenotypes, and size-adjusted phenotypes used for mapping from the BEPA cross.

File S3   Summary of bins in genome assembly (.csv)

A summary of all bins in the genome assembly, and their adjusted positions and orientations in the revised genome assembly. Orientations are defined relative to original genome assembly. The orientation of some scaffolds was not detected in this study. Bins in these scaffolds are labeled as having “unknown” orientation. Chromosome “M” is the mitochondrial genome sequence, which was not analyzed in this study but is replicated in the revised genome assembly.

File S4   Revised consensus scaffold order (.csv)

Revised scaffold order for each chromosome (consensus of FTC and BEPA). Revised coordinates (based on this study) and original assembly coordinates are presented. Orientations are defined relative to original genome assembly. The orientation of some scaffolds was not detected in this study. These scaffolds are labeled as having “unknown” orientation; their orientation was not altered relative to their orientation in the original genome assembly. Chromosome “M” is the mitochondrial genome sequence, which was not analyzed in this study but is replicated in the revised genome assembly.

Files S5-S7

Data available from the Dryad Digital Repository: http://dx.doi.org/10.5061/dryad.q018v

File S5 Revised fasta file of genome assembly (.fa)

Fasta file containing revised genome assembly based on consensus scaffold order and orientation as described in File S4.

File S6 Revised masked fasta file of genome assembly (.fa)

Repeat masked fasta file containing revised genome assembly based on consensus scaffold order and orientation as described in File S4. Repeat masked fasta file is based off the repeat masked version of the original genome assembly, which was masked with RepeatMasker.

File S7 Revised locations of Ensembl-predicted genes (.gtf)

Revised .gtf file of Ensembl gene predictions. Coordinates of gene predictions were converted to the revised assembly coordinates. All Ensembl-predicted genes were included, except ENSGACT00000019430, which spans two scaffolds (11 and 79) that are not adjacent in the revised genome assembly.

File S8

SUPPLEMENTAL METHODS

Cross raising

Lab-reared fish were raised at 18°C in 110 liter (29 gallon) aquaria in a common brackish salinity (3 ppt, 10% ocean water) and fed a common diet of live *Artemia* nauplii and frozen *Daphnia* as fry and juveniles, and bloodworms and *Mysis* shrimp as adults. Adult F2s were raised to a mean size (standard length) of 38.0 mm and 42.1 mm in the FTC and BEPA crosses, respectively. Fish from F1 crosses were intercrossed to generate F2 families ranging in size from 26-108 fish. Adult F2 fish (n= 360 / 363) were sequenced with GBS from 5 and 9 F2 families in the FTC and BEPA crosses, respectively.

Sequencing of cross grandparents and identification of homozygous SNPs

The two grandparents of the FTC cross and the two grandparents of the BEPA cross were resequenced with a Nextera DNA Sample Preparation kit (Illumina) to approximately 60X and 6X coverage, respectively, with 100 base paired-end sequencing on an Illumina HiSeq 2000 sequencer. Reads were mapped to the reference genome with BWA (www.bio-bwa.sourceforge.net). For each grandparent, SNPs relative to the reference genome were called with SAMtools (www.samtools.sourceforge.net). The SNPs in the resulting VCF files were then filtered with a custom Python script (www.python.org) to identify sites where one grandparent was homozygous for the alternate allele (a homozygous alternate SNP was present in the VCF file) and the other grandparent was homozygous for the reference allele (no SNP was present in the VCF file but there was sufficient sequencing coverage to support that the sample was homozygous reference). This analysis identified 1,116,087 and 715,279 sites which were homozygous different between the FTC and BEPA grandparents, respectively ("homozygous SNP positions").

Genotyping from read counts

A likelihood method was adapted from (Hohenlohe et al. 2010), but with only reads supporting the phased marine or freshwater allele considered. Three possible diploid genotypes were considered: G_i=G_{MM}, G_{MF}, or G_{FF} (M=marine allele, F=freshwater allele). Reads were considered to be independent, and have an equal chance of emanating from the two alleles. An error rate of 1% was assumed for each read, which increased the conservativeness of the genotyping calls. Bayes' theorem was used to calculate the probability of each genotype (G_i) given the observed pattern of reads. For example, for the MM genotype: $p(G_{MM}|reads)=p(reads|G_{MM})/[p(reads|G_{MM})+p(reads|G_{MF})+p(reads|G_{FF})]$. If one genotype G_i was most likely with $p(G_i|reads) > 0.95$, the genotype was assigned as G_i. Otherwise, the genotype was assigned as "MF/FF" or "MM/MF" if $p(G_{MM})<0.05$ or $p(G_{FF})<0.05$ (Figure S2). These genotypes were frequently called for bins spanning recombinant breakpoints. If no reads were present, a missing genotype ("NA") was assigned.

Removing markers with aberrant allele ratios

Each marker was tested for deviation from the predicted 1:2:1 allele ratio by a chi-square test. Multiple adjacent markers with allelic distortion likely indicate a true allelic distortion of that genomic region, whereas a single marker with allelic distortion likely indicates genotyping error. Therefore, markers showing significant allelic distortion ($p<0.05$) without any adjacent makers with allelic distortion were removed.

Sex determination

The sex of each F2 fish was determined by calculating the ratio of chromosome 19 (sex chromosome) coverage levels to non-chromosome 19 coverage levels. For females (XX) and males (XY), the theoretical ratios are 1 and 0.5, respectively, with a cutoff of 0.75. Indeed, in each cross the ratio cleanly formed two groups. However, there was slightly more sex chromosome coverage than expected in each cross. Therefore cutoff ratios in each cross were determined manually based on the empirical ratio distribution (0.85 in the FTC cross and 1.15 in the BEPA cross). Fish above and below the cutoff ratio were assigned as female and male, respectively.

Creating a consensus scaffold map

For each cross, the mean genetic positions of each scaffold in the linkage map were compared to create a scaffold map. In cases where multiple markers from the same scaffold were in the linkage map, scaffold orientation was determined by whether correlation between the physical and genetic positions of these markers was significantly positive or negative in a linear regression. A consensus scaffold map was created by merging the largely identical scaffold maps from the two crosses. In cases where the maps disagreed in marker order, the consensus map used the order from the map with the fewest discordant genotypes.

Recombinant breakpoint fine-mapping

Since the markers used for linkage mapping were based on binning multiple SNPs together, a complementary approach was used to fine-map recombinant breakpoints with a Hidden Markov Model (approach adapted from Andolfatto et al. 2011). SNP genotypes were binned into 10 kb bins, near the average density of SNPs used in the study. For each fish, each bin was assigned a raw genotype of M (only marine reads), F (only freshwater reads), B (both marine and freshwater reads), or X (no data). These raw genotypes were used as the observed data in a Hidden Markov Model with hidden states M (marine), F (freshwater), or H (heterozygous). The model was trained with the Baum-Welch algorithm on 1 million data points. For each fish and bin, the model was used to calculate the probability of each hidden state with the forward-backward algorithm. Details on the trained parameters of the model are presented in Figure S6D. Locations within a scaffold where the probability switched from over 99% probability of state A to over 99% probability of state B were considered to be the boundaries of a recombination event.

Calling sex chromosome genotypes

The stickleback sex chromosome (chromosome 19) consists of a small pseudo-autosomal region and a large region that behaves like a sex chromosome (Peichel et al. 2004; Roesti et al. 2013).

The cutoff for the boundary between these regions was chosen at 2.41 Mb, based on levels of sequencing coverage in male and female F2s. Genotypes for the pseudo-autosomal region were determined with the same method as the autosomal chromosomes, whereas a separate pipeline was used for the rest of the sex chromosome. First, reads that mapped to the X chromosome but not the Y chromosome were identified by sieving for SNPs that had an approximately 2:1 ratio of females to male coverage (SNPs that had female:male average RPMM ratios between 1.5 and 3.5). Female F2s should have a 3:1 ratio of X chromosomes originating from their grandmother:grandfather, whereas male F2s should have a 1:1 ratio. Therefore, a 3:1 ratio of alleles was filtered for in female F2s (average RPMM between 2 and 6) and a 1:1 ratio was filtered for in male F2s (average RPMM between 0.2 and 4). Finally, since all male F2s have a Y chromosome that originated from their grandfather, male F2 genotypes that were genotyped as homozygous for their grandmother's allele were converted to heterozygous.

Using read correlations to anchor scaffolds

In addition to the linkage maps made with JoinMap, a second more sensitive, but less precise, method was used to further determine the genomic location of scaffolds. This method enabled the mapping of even more scaffolds, but did not reveal the scaffolds' precise genomic location and orientation. Every pair of markers was considered ("marker 1" and "marker 2"). Fish genotyped as homozygous marine or homozygous freshwater for marker 1 were examined. For these fish, raw read counts for marker 2 were summed and were scored as concordant or discordant with the marker 1 genotype. If the total concordance percentage was above 95%, marker 2 was considered linked to marker 1 with ~<5 cM distance. In 300 out of 302 cases (99.3%) where a scaffold mapped to a chromosome by this method in both crosses, the chromosome was the same. The other two cases were likely due to reads mapping to repetitive genomic segments (Table S3).

Bone staining

To stain bones, fish were fixed for 1-2 days in 10% neutral buffered formalin or 5-7 days in 4% paraformaldehyde in 1xPBS, washed with water overnight, stained overnight with 0.008% Alizarin Red S in 1% potassium hydroxide, destained in water overnight, and cleared in a 0.25% potassium hydroxide, 50% glycerol solution.

Phenotype processing

For QTL mapping, plate number and gill raker length phenotypes were tested for an association with standard length and sex by linear regression in R (www.r-project.org) and corrected for size, sex, and/or log transformed, when appropriate. When association with standard length was significant ($p<0.05$), residuals were taken from a linear regression, then back-transformed to values expected for a 40 mm (marine x freshwater F2s) or 50 mm (lab-reared incross) standard length fish. When correlations with standard length and sex were both significant in a linear model, both were corrected for. When log transformation improved the normality of the residuals (i.e. by resulting in an Anderson-Darling test changing from $p<0.05$ to $p>0.05$), log transformation was performed.

REFERENCES

Andolfatto, P., D. Davison, D. Erezyilmaz, T.T. Hu, J. Mast *et al.*, 2011 Multiplexed shotgun genotyping for rapid and efficient genetic mapping. Genome Res. 21: 610-617.

Hohenlohe, P.A., S. Bassham, P.D. Etter, N. Stiffler, E.A. Johnson *et al.*, 2010 Population genomics of parallel adaptation in threespine stickleback using sequenced RAD tags. PLoS Genet. 6: e1000862.

Jones, F.C., M.G. Grabherr, Y.F. Chan, P. Russell, E. Mauceli *et al.*, 2012 The genomic basis of adaptive evolution in threespine sticklebacks. Nature 484: 55-61.

Peichel, C.L., J.A. Ross, C.K. Matson, M. Dickson, J. Grimwood *et al.*, 2004 The master sex-determination locus in threespine sticklebacks is on a nascent Y chromosome. Current Biology 14: 1416-1424.

Roesti, M., D. Moser, and D. Berner, 2013 Recombination in the threespine stickleback genome--patterns and consequences. Mol. Ecol. 22: 3014-3027.

Table S1 Primers used in this study. Primers were used for PCR, index sequencing ("Index"), or making adapters.

Name	Primer	Type	Barcode
PCR1	CAAGCAGAAGACGGCATACGAGATCGGTCTCGGCATTCCTGCTGAACCGCTCTTCCGATCT	PCR (no indexing)	NA
PCR1_index1	CAAGCAGAAGACGGCATACGAGATcgtgatCGGTCTCGGCATTCCTGCTGAACCGCTCTTCCGATCT	PCR (indexing)	NA
PCR1_index2	CAAGCAGAAGACGGCATACGAGATacatcgCGGTCTCGGCATTCCTGCTGAACCGCTCTTCCGATCT	PCR (indexing)	NA
PCR1_index3	CAAGCAGAAGACGGCATACGAGATgcctaaCGGTCTCGGCATTCCTGCTGAACCGCTCTTCCGATCT	PCR (indexing)	NA
PCR1_index4	CAAGCAGAAGACGGCATACGAGATtggtcaCGGTCTCGGCATTCCTGCTGAACCGCTCTTCCGATCT	PCR (indexing)	NA
PCR2	AATGATACGGCGACCACCGAGATCTACACTCTTTCCCTACACGACGCTCTTCCGATCT	PCR (indexing/no indexing)	NA
Seq_index	GATCGGAAGAGCGGTTCAGCAGGAATGCCGAGACCG	Index	NA
ApeK1Y_A1F	ACACTCTTTCCCTACACGACGCTCTTCCGATCTctcc	Adapter	ctcc
ApeK1Y_A2F	ACACTCTTTCCCTACACGACGCTCTTCCGATCTtgca	Adapter	tgca
ApeK1Y_A3F	ACACTCTTTCCCTACACGACGCTCTTCCGATCTacta	Adapter	acta
ApeK1Y_A4F	ACACTCTTTCCCTACACGACGCTCTTCCGATCTcaga	Adapter	caga
ApeK1Y_A5F	ACACTCTTTCCCTACACGACGCTCTTCCGATCTaact	Adapter	aact
ApeK1Y_A6F	ACACTCTTTCCCTACACGACGCTCTTCCGATCTgcgt	Adapter	gcgt
ApeK1Y_A7F	ACACTCTTTCCCTACACGACGCTCTTCCGATCTcgat	Adapter	cgat
ApeK1Y_A8F	ACACTCTTTCCCTACACGACGCTCTTCCGATCTgtaa	Adapter	gtaa
ApeK1Y_A9F	ACACTCTTTCCCTACACGACGCTCTTCCGATCTtgcga	Adapter	tgcga
ApeK1Y_A10F	ACACTCTTTCCCTACACGACGCTCTTCCGATCTcgctt	Adapter	cgctt
ApeK1Y_A11F	ACACTCTTTCCCTACACGACGCTCTTCCGATCTtcacc	Adapter	tcacc
ApeK1Y_A12F	ACACTCTTTCCCTACACGACGCTCTTCCGATCTctagc	Adapter	ctagc
ApeK1Y_B1F	ACACTCTTTCCCTACACGACGCTCTTCCGATCTacaaa	Adapter	acaaa
ApeK1Y_B2F	ACACTCTTTCCCTACACGACGCTCTTCCGATCTttctc	Adapter	ttctc
ApeK1Y_B3F	ACACTCTTTCCCTACACGACGCTCTTCCGATCTagccc	Adapter	agccc
ApeK1Y_B4F	ACACTCTTTCCCTACACGACGCTCTTCCGATCTgtatt	Adapter	gtatt
ApeK1Y_B5F	ACACTCTTTCCCTACACGACGCTCTTCCGATCTctgta	Adapter	ctgta
ApeK1Y_B6F	ACACTCTTTCCCTACACGACGCTCTTCCGATCTaccgt	Adapter	accgt
ApeK1Y_B7F	ACACTCTTTCCCTACACGACGCTCTTCCGATCTgctta	Adapter	gctta
ApeK1Y_B8F	ACACTCTTTCCCTACACGACGCTCTTCCGATCTggtgt	Adapter	ggtgt
ApeK1Y_B9F	ACACTCTTTCCCTACACGACGCTCTTCCGATCTggttgt	Adapter	ggttgt
ApeK1Y_B10F	ACACTCTTTCCCTACACGACGCTCTTCCGATCTccagct	Adapter	ccagct
ApeK1Y_B11F	ACACTCTTTCCCTACACGACGCTCTTCCGATCTttcaga	Adapter	ttcaga
ApeK1Y_B12F	ACACTCTTTCCCTACACGACGCTCTTCCGATCTtaggaa	Adapter	taggaa
ApeK1Y_C1F	ACACTCTTTCCCTACACGACGCTCTTCCGATCTgctcta	Adapter	gctcta
ApeK1Y_C2F	ACACTCTTTCCCTACACGACGCTCTTCCGATCTccacaa	Adapter	ccacaa
ApeK1Y_C3F	ACACTCTTTCCCTACACGACGCTCTTCCGATCTcttcca	Adapter	cttcca
ApeK1Y_C4F	ACACTCTTTCCCTACACGACGCTCTTCCGATCTgagata	Adapter	gagata
ApeK1Y_C5F	ACACTCTTTCCCTACACGACGCTCTTCCGATCTatgcct	Adapter	atgcct
ApeK1Y_C6F	ACACTCTTTCCCTACACGACGCTCTTCCGATCTagtgga	Adapter	agtgga
ApeK1Y_C7F	ACACTCTTTCCCTACACGACGCTCTTCCGATCTtattttt	Adapter	tattttt
ApeK1Y_C8F	ACACTCTTTCCCTACACGACGCTCTTCCGATCTcttgctt	Adapter	cttgctt
ApeK1Y_C9F	ACACTCTTTCCCTACACGACGCTCTTCCGATCTatgaaac	Adapter	atgaaac
ApeK1Y_C10F	ACACTCTTTCCCTACACGACGCTCTTCCGATCTaaaagtt	Adapter	aaaagtt
ApeK1Y_C11F	ACACTCTTTCCCTACACGACGCTCTTCCGATCTgaattca	Adapter	gaattca
ApeK1Y_C12F	ACACTCTTTCCCTACACGACGCTCTTCCGATCTgaacttc	Adapter	gaacttc
ApeK1Y_D1F	ACACTCTTTCCCTACACGACGCTCTTCCGATCTggaccta	Adapter	ggaccta
ApeK1Y_D2F	ACACTCTTTCCCTACACGACGCTCTTCCGATCTgtcgatt	Adapter	gtcgatt
ApeK1Y_D3F	ACACTCTTTCCCTACACGACGCTCTTCCGATCTaacgcct	Adapter	aacgcct
ApeK1Y_D4F	ACACTCTTTCCCTACACGACGCTCTTCCGATCTaatatgc	Adapter	aatatgc
ApeK1Y_D5F	ACACTCTTTCCCTACACGACGCTCTTCCGATCTacgactac	Adapter	acgactac

ApeK1Y_D6F	ACACTCTTTCCCTACACGACGCTCTTCCGATCTtagcatgc	Adapter	tagcatgc
ApeK1Y_D7F	ACACTCTTTCCCTACACGACGCTCTTCCGATCTtaggccat	Adapter	taggccat
ApeK1Y_D8F	ACACTCTTTCCCTACACGACGCTCTTCCGATCTtgcaagga	Adapter	tgcaagga
ApeK1Y_D9F	ACACTCTTTCCCTACACGACGCTCTTCCGATCTtggtacgt	Adapter	tggtacgt
ApeK1Y_D10F	ACACTCTTTCCCTACACGACGCTCTTCCGATCTtctcagtc	Adapter	tctcagtc
ApeK1Y_D11F	ACACTCTTTCCCTACACGACGCTCTTCCGATCTccggatat	Adapter	ccggatat
ApeK1Y_D12F	ACACTCTTTCCCTACACGACGCTCTTCCGATCTcgccttat	Adapter	cgccttat
ApeK1Y_E1F	ACACTCTTTCCCTACACGACGCTCTTCCGATCTaggc	Adapter	aggc
ApeK1Y_E2F	ACACTCTTTCCCTACACGACGCTCTTCCGATCTgatc	Adapter	gatc
ApeK1Y_E3F	ACACTCTTTCCCTACACGACGCTCTTCCGATCTtcac	Adapter	tcac
ApeK1Y_E4F	ACACTCTTTCCCTACACGACGCTCTTCCGATCTaggat	Adapter	aggat
ApeK1Y_E5F	ACACTCTTTCCCTACACGACGCTCTTCCGATCTattga	Adapter	attga
ApeK1Y_E6F	ACACTCTTTCCCTACACGACGCTCTTCCGATCTcatct	Adapter	catct
ApeK1Y_E7F	ACACTCTTTCCCTACACGACGCTCTTCCGATCTcctac	Adapter	cctac
ApeK1Y_E8F	ACACTCTTTCCCTACACGACGCTCTTCCGATCTgagga	Adapter	gagga
ApeK1Y_E9F	ACACTCTTTCCCTACACGACGCTCTTCCGATCTggaac	Adapter	ggaac
ApeK1Y_E10F	ACACTCTTTCCCTACACGACGCTCTTCCGATCTgtcaa	Adapter	gtcaa
ApeK1Y_E11F	ACACTCTTTCCCTACACGACGCTCTTCCGATCTtaata	Adapter	taata
ApeK1Y_E12F	ACACTCTTTCCCTACACGACGCTCTTCCGATCTtacat	Adapter	tacat
ApeK1Y_F1F	ACACTCTTTCCCTACACGACGCTCTTCCGATCTtcgtt	Adapter	tcgtt
ApeK1Y_F2F	ACACTCTTTCCCTACACGACGCTCTTCCGATCTacctaa	Adapter	acctaa
ApeK1Y_F3F	ACACTCTTTCCCTACACGACGCTCTTCCGATCTatatgt	Adapter	atatgt
ApeK1Y_F4F	ACACTCTTTCCCTACACGACGCTCTTCCGATCTatcgta	Adapter	atcgta
ApeK1Y_F5F	ACACTCTTTCCCTACACGACGCTCTTCCGATCTcatcgt	Adapter	catcgt
ApeK1Y_F6F	ACACTCTTTCCCTACACGACGCTCTTCCGATCTcgcggt	Adapter	cgcggt
ApeK1Y_F7F	ACACTCTTTCCCTACACGACGCTCTTCCGATCTctatta	Adapter	ctatta
ApeK1Y_F8F	ACACTCTTTCCCTACACGACGCTCTTCCGATCTgccagt	Adapter	gccagt
ApeK1Y_F9F	ACACTCTTTCCCTACACGACGCTCTTCCGATCTggaaga	Adapter	ggaaga
ApeK1Y_F10F	ACACTCTTTCCCTACACGACGCTCTTCCGATCTgtactt	Adapter	gtactt
ApeK1Y_F11F	ACACTCTTTCCCTACACGACGCTCTTCCGATCTgttgaa	Adapter	gttgaa
ApeK1Y_F12F	ACACTCTTTCCCTACACGACGCTCTTCCGATCTtaacga	Adapter	taacga
ApeK1Y_G1F	ACACTCTTTCCCTACACGACGCTCTTCCGATCTtggcta	Adapter	tggcta
ApeK1Y_G2F	ACACTCTTTCCCTACACGACGCTCTTCCGATCTacgtgtt	Adapter	acgtgtt
ApeK1Y_G3F	ACACTCTTTCCCTACACGACGCTCTTCCGATCTattaatt	Adapter	attaatt
ApeK1Y_G4F	ACACTCTTTCCCTACACGACGCTCTTCCGATCTattggat	Adapter	attggat
ApeK1Y_G5F	ACACTCTTTCCCTACACGACGCTCTTCCGATCTcataagt	Adapter	cataagt
ApeK1Y_G6F	ACACTCTTTCCCTACACGACGCTCTTCCGATCTcgctgat	Adapter	cgctgat
ApeK1Y_G7F	ACACTCTTTCCCTACACGACGCTCTTCCGATCTcggtaga	Adapter	cggtaga
ApeK1Y_G8F	ACACTCTTTCCCTACACGACGCTCTTCCGATCTctacgga	Adapter	ctacgga
ApeK1Y_G9F	ACACTCTTTCCCTACACGACGCTCTTCCGATCTgcggaat	Adapter	gcggaat
ApeK1Y_G10F	ACACTCTTTCCCTACACGACGCTCTTCCGATCTtagcgga	Adapter	tagcgga
ApeK1Y_G11F	ACACTCTTTCCCTACACGACGCTCTTCCGATCTtcgaaga	Adapter	tcgaaga
ApeK1Y_G12F	ACACTCTTTCCCTACACGACGCTCTTCCGATCTtctgtga	Adapter	tctgtga
ApeK1Y_H1F	ACACTCTTTCCCTACACGACGCTCTTCCGATCTtgctgga	Adapter	tgctgga
ApeK1Y_H2F	ACACTCTTTCCCTACACGACGCTCTTCCGATCTaaccgaga	Adapter	aaccgaga
ApeK1Y_H3F	ACACTCTTTCCCTACACGACGCTCTTCCGATCTacagggaa	Adapter	acagggaa
ApeK1Y_H4F	ACACTCTTTCCCTACACGACGCTCTTCCGATCTacgtggta	Adapter	acgtggta
ApeK1Y_H5F	ACACTCTTTCCCTACACGACGCTCTTCCGATCTccatgggt	Adapter	ccatgggt
ApeK1Y_H6F	ACACTCTTTCCCTACACGACGCTCTTCCGATCTcgcggaga	Adapter	cgcggaga
ApeK1Y_H7F	ACACTCTTTCCCTACACGACGCTCTTCCGATCTcgtgtggt	Adapter	cgtgtggt
ApeK1Y_H8F	ACACTCTTTCCCTACACGACGCTCTTCCGATCTgctgtgga	Adapter	gctgtgga
ApeK1Y_H9F	ACACTCTTTCCCTACACGACGCTCTTCCGATCTggattggt	Adapter	ggattggt
ApeK1Y_H10F	ACACTCTTTCCCTACACGACGCTCTTCCGATCTgtgagggt	Adapter	gtgagggt
ApeK1Y_H11F	ACACTCTTTCCCTACACGACGCTCTTCCGATCTtatcggga	Adapter	tatcggga
ApeK1Y_H12F	ACACTCTTTCCCTACACGACGCTCTTCCGATCTttcctgga	Adapter	ttcctgga
ApeK1Y_A1R	pCWGggagAGATCGGAAGAGCGGTTCAGCAGGAATGCCGAG	Adapter	NA
ApeK1Y_A2R	pCWGtgcaAGATCGGAAGAGCGGTTCAGCAGGAATGCCGAG	Adapter	NA
ApeK1Y_A3R	pCWGtagtAGATCGGAAGAGCGGTTCAGCAGGAATGCCGAG	Adapter	NA

ApeK1Y_A4R	pCWGtctgAGATCGGAAGAGCGGTTCAGCAGGAATGCCGAG	Adapter	NA
ApeK1Y_A5R	pCWGagttAGATCGGAAGAGCGGTTCAGCAGGAATGCCGAG	Adapter	NA
ApeK1Y_A6R	pCWGacgcAGATCGGAAGAGCGGTTCAGCAGGAATGCCGAG	Adapter	NA
ApeK1Y_A7R	pCWGatcgAGATCGGAAGAGCGGTTCAGCAGGAATGCCGAG	Adapter	NA
ApeK1Y_A8R	pCWGttacAGATCGGAAGAGCGGTTCAGCAGGAATGCCGAG	Adapter	NA
ApeK1Y_A9R	pCWGtcgcaAGATCGGAAGAGCGGTTCAGCAGGAATGCCGAG	Adapter	NA
ApeK1Y_A10R	pCWGaagcgAGATCGGAAGAGCGGTTCAGCAGGAATGCCGAG	Adapter	NA
ApeK1Y_A11R	pCWGggtgaAGATCGGAAGAGCGGTTCAGCAGGAATGCCGAG	Adapter	NA
ApeK1Y_A12R	pCWGgctagAGATCGGAAGAGCGGTTCAGCAGGAATGCCGAG	Adapter	NA
ApeK1Y_B1R	pCWGtttgtAGATCGGAAGAGCGGTTCAGCAGGAATGCCGAG	Adapter	NA
ApeK1Y_B2R	pCWGgagaaAGATCGGAAGAGCGGTTCAGCAGGAATGCCGAG	Adapter	NA
ApeK1Y_B3R	pCWGgggctAGATCGGAAGAGCGGTTCAGCAGGAATGCCGAG	Adapter	NA
ApeK1Y_B4R	pCWGaatacAGATCGGAAGAGCGGTTCAGCAGGAATGCCGAG	Adapter	NA
ApeK1Y_B5R	pCWGtacagAGATCGGAAGAGCGGTTCAGCAGGAATGCCGAG	Adapter	NA
ApeK1Y_B6R	pCWGacggtAGATCGGAAGAGCGGTTCAGCAGGAATGCCGAG	Adapter	NA
ApeK1Y_B7R	pCWGtaagcAGATCGGAAGAGCGGTTCAGCAGGAATGCCGAG	Adapter	NA
ApeK1Y_B8R	pCWGacaccAGATCGGAAGAGCGGTTCAGCAGGAATGCCGAG	Adapter	NA
ApeK1Y_B9R	pCWGacaaccAGATCGGAAGAGCGGTTCAGCAGGAATGCCGAG	Adapter	NA
ApeK1Y_B10R	pCWGagctggAGATCGGAAGAGCGGTTCAGCAGGAATGCCGAG	Adapter	NA
ApeK1Y_B11R	pCWGtctgaaAGATCGGAAGAGCGGTTCAGCAGGAATGCCGAG	Adapter	NA
ApeK1Y_B12R	pCWGttcctaAGATCGGAAGAGCGGTTCAGCAGGAATGCCGAG	Adapter	NA
ApeK1Y_C1R	pCWGtagagcAGATCGGAAGAGCGGTTCAGCAGGAATGCCGAG	Adapter	NA
ApeK1Y_C2R	pCWGttgtggAGATCGGAAGAGCGGTTCAGCAGGAATGCCGAG	Adapter	NA
ApeK1Y_C3R	pCWGtggaagAGATCGGAAGAGCGGTTCAGCAGGAATGCCGAG	Adapter	NA
ApeK1Y_C4R	pCWGtatctcAGATCGGAAGAGCGGTTCAGCAGGAATGCCGAG	Adapter	NA
ApeK1Y_C5R	pCWGaggcatAGATCGGAAGAGCGGTTCAGCAGGAATGCCGAG	Adapter	NA
ApeK1Y_C6R	pCWGtccactAGATCGGAAGAGCGGTTCAGCAGGAATGCCGAG	Adapter	NA
ApeK1Y_C7R	pCWGaaaaataAGATCGGAAGAGCGGTTCAGCAGGAATGCCGAG	Adapter	NA
ApeK1Y_C8R	pCWGaagcaagAGATCGGAAGAGCGGTTCAGCAGGAATGCCGAG	Adapter	NA
ApeK1Y_C9R	pCWGgtttcatAGATCGGAAGAGCGGTTCAGCAGGAATGCCGAG	Adapter	NA
ApeK1Y_C10R	pCWGaacttttAGATCGGAAGAGCGGTTCAGCAGGAATGCCGAG	Adapter	NA
ApeK1Y_C11R	pCWGtgaattcAGATCGGAAGAGCGGTTCAGCAGGAATGCCGAG	Adapter	NA
ApeK1Y_C12R	pCWGgaagttcAGATCGGAAGAGCGGTTCAGCAGGAATGCCGAG	Adapter	NA
ApeK1Y_D1R	pCWGtaggtccAGATCGGAAGAGCGGTTCAGCAGGAATGCCGAG	Adapter	NA
ApeK1Y_D2R	pCWGaatcgacAGATCGGAAGAGCGGTTCAGCAGGAATGCCGAG	Adapter	NA
ApeK1Y_D3R	pCWGaggcgttAGATCGGAAGAGCGGTTCAGCAGGAATGCCGAG	Adapter	NA
ApeK1Y_D4R	pCWGcatattAGATCGGAAGAGCGGTTCAGCAGGAATGCCGAG	Adapter	NA
ApeK1Y_D5R	pCWGgtagtcgtAGATCGGAAGAGCGGTTCAGCAGGAATGCCGAG	Adapter	NA
ApeK1Y_D6R	pCWGgcatgctaAGATCGGAAGAGCGGTTCAGCAGGAATGCCGAG	Adapter	NA
ApeK1Y_D7R	pCWGatggcctaAGATCGGAAGAGCGGTTCAGCAGGAATGCCGAG	Adapter	NA
ApeK1Y_D8R	pCWGtccttgcaAGATCGGAAGAGCGGTTCAGCAGGAATGCCGAG	Adapter	NA
ApeK1Y_D9R	pCWGacgtaccaAGATCGGAAGAGCGGTTCAGCAGGAATGCCGAG	Adapter	NA
ApeK1Y_D10R	pCWGgactgagaAGATCGGAAGAGCGGTTCAGCAGGAATGCCGAG	Adapter	NA
ApeK1Y_D11R	pCWGatatccggAGATCGGAAGAGCGGTTCAGCAGGAATGCCGAG	Adapter	NA
ApeK1Y_D12R	pCWGataaggcgAGATCGGAAGAGCGGTTCAGCAGGAATGCCGAG	Adapter	NA
ApeK1Y_E1R	pCWGgcctAGATCGGAAGAGCGGTTCAGCAGGAATGCCGAG	Adapter	NA
ApeK1Y_E2R	pCWGgatcAGATCGGAAGAGCGGTTCAGCAGGAATGCCGAG	Adapter	NA
ApeK1Y_E3R	pCWGgtgaAGATCGGAAGAGCGGTTCAGCAGGAATGCCGAG	Adapter	NA
ApeK1Y_E4R	pCWGatcctAGATCGGAAGAGCGGTTCAGCAGGAATGCCGAG	Adapter	NA
ApeK1Y_E5R	pCWGtcaatAGATCGGAAGAGCGGTTCAGCAGGAATGCCGAG	Adapter	NA
ApeK1Y_E6R	pCWGagatgAGATCGGAAGAGCGGTTCAGCAGGAATGCCGAG	Adapter	NA
ApeK1Y_E7R	pCWGgtaggAGATCGGAAGAGCGGTTCAGCAGGAATGCCGAG	Adapter	NA
ApeK1Y_E8R	pCWGtcctcAGATCGGAAGAGCGGTTCAGCAGGAATGCCGAG	Adapter	NA
ApeK1Y_E9R	pCWGgttccAGATCGGAAGAGCGGTTCAGCAGGAATGCCGAG	Adapter	NA
ApeK1Y_E10R	pCWGttgacAGATCGGAAGAGCGGTTCAGCAGGAATGCCGAG	Adapter	NA
ApeK1Y_E11R	pCWGtattaAGATCGGAAGAGCGGTTCAGCAGGAATGCCGAG	Adapter	NA
ApeK1Y_E12R	pCWGatgtaAGATCGGAAGAGCGGTTCAGCAGGAATGCCGAG	Adapter	NA
ApeK1Y_F1R	pCWGaacgaAGATCGGAAGAGCGGTTCAGCAGGAATGCCGAG	Adapter	NA

ApeK1Y_F2R	pCWGttaggtAGATCGGAAGAGCGGTTCAGCAGGAATGCCGAG	Adapter	NA
ApeK1Y_F3R	pCWGacatatAGATCGGAAGAGCGGTTCAGCAGGAATGCCGAG	Adapter	NA
ApeK1Y_F4R	pCWGtacgatAGATCGGAAGAGCGGTTCAGCAGGAATGCCGAG	Adapter	NA
ApeK1Y_F5R	pCWGacgatgAGATCGGAAGAGCGGTTCAGCAGGAATGCCGAG	Adapter	NA
ApeK1Y_F6R	pCWGaccgcgAGATCGGAAGAGCGGTTCAGCAGGAATGCCGAG	Adapter	NA
ApeK1Y_F7R	pCWGtaatagAGATCGGAAGAGCGGTTCAGCAGGAATGCCGAG	Adapter	NA
ApeK1Y_F8R	pCWGactggcAGATCGGAAGAGCGGTTCAGCAGGAATGCCGAG	Adapter	NA
ApeK1Y_F9R	pCWGtcttccAGATCGGAAGAGCGGTTCAGCAGGAATGCCGAG	Adapter	NA
ApeK1Y_F10R	pCWGaagtacAGATCGGAAGAGCGGTTCAGCAGGAATGCCGAG	Adapter	NA
ApeK1Y_F11R	pCWGttcaacAGATCGGAAGAGCGGTTCAGCAGGAATGCCGAG	Adapter	NA
ApeK1Y_F12R	pCWGtcgttaAGATCGGAAGAGCGGTTCAGCAGGAATGCCGAG	Adapter	NA
ApeK1Y_G1R	pCWGtagccaAGATCGGAAGAGCGGTTCAGCAGGAATGCCGAG	Adapter	NA
ApeK1Y_G2R	pCWGaacacgtAGATCGGAAGAGCGGTTCAGCAGGAATGCCGAG	Adapter	NA
ApeK1Y_G3R	pCWGaattaatAGATCGGAAGAGCGGTTCAGCAGGAATGCCGAG	Adapter	NA
ApeK1Y_G4R	pCWGatccaatAGATCGGAAGAGCGGTTCAGCAGGAATGCCGAG	Adapter	NA
ApeK1Y_G5R	pCWGacttatgAGATCGGAAGAGCGGTTCAGCAGGAATGCCGAG	Adapter	NA
ApeK1Y_G6R	pCWGatcagcgAGATCGGAAGAGCGGTTCAGCAGGAATGCCGAG	Adapter	NA
ApeK1Y_G7R	pCWGtctaccgAGATCGGAAGAGCGGTTCAGCAGGAATGCCGAG	Adapter	NA
ApeK1Y_G8R	pCWGtccgtagAGATCGGAAGAGCGGTTCAGCAGGAATGCCGAG	Adapter	NA
ApeK1Y_G9R	pCWGattccgcAGATCGGAAGAGCGGTTCAGCAGGAATGCCGAG	Adapter	NA
ApeK1Y_G10R	pCWGtccgctaAGATCGGAAGAGCGGTTCAGCAGGAATGCCGAG	Adapter	NA
ApeK1Y_G11R	pCWGtcttcgaAGATCGGAAGAGCGGTTCAGCAGGAATGCCGAG	Adapter	NA
ApeK1Y_G12R	pCWGtcacagaAGATCGGAAGAGCGGTTCAGCAGGAATGCCGAG	Adapter	NA
ApeK1Y_H1R	pCWGtccagcaAGATCGGAAGAGCGGTTCAGCAGGAATGCCGAG	Adapter	NA
ApeK1Y_H2R	pCWGtctcggttAGATCGGAAGAGCGGTTCAGCAGGAATGCCGAG	Adapter	NA
ApeK1Y_H3R	pCWGttccctgtAGATCGGAAGAGCGGTTCAGCAGGAATGCCGAG	Adapter	NA
ApeK1Y_H4R	pCWGtaccacgtAGATCGGAAGAGCGGTTCAGCAGGAATGCCGAG	Adapter	NA
ApeK1Y_H5R	pCWGacccatggAGATCGGAAGAGCGGTTCAGCAGGAATGCCGAG	Adapter	NA
ApeK1Y_H6R	pCWGtctccgcgAGATCGGAAGAGCGGTTCAGCAGGAATGCCGAG	Adapter	NA
ApeK1Y_H7R	pCWGaccacacgAGATCGGAAGAGCGGTTCAGCAGGAATGCCGAG	Adapter	NA
ApeK1Y_H8R	pCWGtccacagcAGATCGGAAGAGCGGTTCAGCAGGAATGCCGAG	Adapter	NA
ApeK1Y_H9R	pCWGaccaatccAGATCGGAAGAGCGGTTCAGCAGGAATGCCGAG	Adapter	NA
ApeK1Y_H10R	pCWGaccctcacAGATCGGAAGAGCGGTTCAGCAGGAATGCCGAG	Adapter	NA
ApeK1Y_H11R	pCWGtcccgataAGATCGGAAGAGCGGTTCAGCAGGAATGCCGAG	Adapter	NA
ApeK1Y_H12R	pCWGtccaggaaAGATCGGAAGAGCGGTTCAGCAGGAATGCCGAG	Adapter	NA

Table S2 Cross summary statistics. Numbers of initial samples, dropped samples due to low coverage, and final samples are presented. Numbers of initial markers, dropped markers due to low coverage and skewed allele ratios, and final markers are presented. GP=grandparent. Genotype fail % = percentage of missing genotypes.

			Samples			Markers						
**Cross**	**GP SNPs**	**F2 SNPs used**	**Initial**	**Low coverage**	**Final**	**Initial**	**Low coverage**	**Skewed allele ratio**	**Final**	**SNP Coverage**	**Marker Coverage**	**Genotype Fail %**
FTC	1,116,087	131,091	358	2	356	1,085	59	25	1,001	1.63X	197X	1.9
BEPA	715,279	87,419	361	2	359	1,077	39	60	978	1.73X	140X	1.8

Table S3 Anchored scaffolds

Details of 538 scaffolds that are linked to a chromosome. Some of these scaffolds were mapped in the core FTC and/or BEPA maps—the centiMorgan (cM) range and orientation (if known) of these scaffolds is shown under "Map cM". A further approach (see Methods) used read correlations to anchor additional scaffolds. For scaffolds anchored with this approach, the cM position of the highest correlating marker in the core map is presented under "RC cM". "Markers" indicates the number of binned markers in the scaffold.

	Original location			Revised location			FTC Cross			BEPA Cross			
Scaffold	Chr	Position	Length	Chr	Orientation	Markers	SNPs	Map cM	RC cM	SNPs	Map cM	RC cM	Note
0	2	966608-22375856	21,409,249	2	Forward	43	5,763	1.0-76.4	27.6	3,803	3.4-92.2	37.9	
1	7	10411930-27613546	17,201,617	7	Forward	35	9,402	48.8-95.1	50.1	6,263	69.7-122.1	74.1	
2	20	834142-17950836	17,116,695	20	Reverse	35	7,599	21.6-74.6	73.3	4,465	29.4-91.6	64.9	
3	19	3824254-20240660	16,416,407	19	Reverse	33	5,191	41.5-74.3	43.9	2,541	55.2-89.5	61.7	
4	13	2825158-18481171	15,656,014	13	Forward	32	3,748	3.5-39.2	21.1	2,810	12.5-54.5	29.7	
5	10	1-14541209	14,541,209	10	Forward	30	2,971	0.0-62.2	25.3	3,081	6.1-83.9	32	
6	12	2209412-16547614	14,338,203	12	Reverse	29	5,301	46.0-68.0	66.5	3,554	52.4-79.2	53.1	
7	1	5935398-19921301	13,985,904	1	Forward	28	10,293	47.7-54.1	51.2	2,876	53.0-73.8	73.8	
8	9	4309200-17894190	13,584,991	9	Reverse	28	3,359	20.5-47.2	20.5	3,242	35.2-70.0	41.8	
9	8	4501841-18121348	13,619,508	8	Forward	28	5,214	22.8-60.8	29.5	3,390	30.0-77.2	37.4	
10	15	1070869-14379835	13,308,967	15	Forward	27	1,814	20.8-65.3	53.5	1,975	18.5-71.1	56.1	
11	11	2205540-15151913	12,946,374	11	Forward	26	5,117	3.2-40.5	15.1	4,125	8.6-57.8	25.8	
12	14	346722-13082935	12,736,214	14	Forward	26	1,560	6.7-67.0	64.7	1,860	5.6-62.6	49.8	
13	6	4057780-16417534	12,359,755	6	Forward	25	2,362	10.7-54.3	18.9	1,804	23.3-74.4	31.9	
14	16	5282403-16168747	10,886,345	16	Forward	22	2,851	21.9-38.1	30	1,679	26.9-51.3	51.3	
15	3	3269601-14097026	10,827,426	3	Forward	22	1,875	12.8-31.8	17.5	1,628	22.8-41.8	28.8	
16	21	298071-9494732	9,196,662	21	Forward	19	6,476	10.9-23.2	11.6	4,166	13.0-28.2	17.9	
17	4	9036585-17819056	8,782,472	4	Forward	18	6,266	51.6-58.6	58.1	3,651	49.5-63.1	62.8	
18	17	6449158-14603141	8,153,984	17	Forward	17	1,542	34.2-69.1	69.1	1,218	53.6-101.8	53.6	
19	5	1-7877689	7,877,689	5	Reverse	16	2,242	6.0-19.3	11.1	1,534	12.9-25.3	14.4	
20	4	1174335-9035584	7,861,250	4	Forward	16	2,790	7.6-51.5	51.6	1,760	8.6-49.3	49.3	
21	18	4823595-12228020	7,404,426	18	Forward	15	1,065	17.1-29.6	28.5	928	28.2-43.0	28.5	
22	1	19922302-27290228	7,367,927	1	Reverse	15	2,431	52.0-71.6	58.4	2,239	75.1-104.9	91.2	
23	7	4609159-10410929	5,801,771	7	Forward	12	3,917	45.4-48.8	48.5	1,652	63.7-69.3	69.1	
24	4	22731829-28355687	5,623,859	4	Reverse	12	3,876	58.8-59.2	59.1	2,145	63.8-67.5	66.7	
25	17	936214-6448157	5,511,944	17	Forward	12	938	11.5-33.6	32.7	1,007	17.7-52.8	39.6	
26	16	1-5281402	5,281,402	16	Forward	11	743	0.0-20.8	1.3	920	0.0-25.2	9.5	
27	Un	1-5105261	5,105,261	17	Reverse	11	2,806	33.4-34.3	34.2	329	52.7-53.8	53.6	
28	4	17820057-22730828	4,910,772	4	Reverse	10	3,585	59.2-60.7	59.3	2,177	67.3-71.1	68.5	
29	18	1-4822594	4,822,594	18	Forward	10	738	0.0-17.1	17.1	816	0.0-28.5	28.5	
30	1	1545350-5934397	4,389,048	1	Forward	9	654	18.8-46.4	45	928	20.9-52.3	49.2	
31	9	1-4308199	4,308,199	9	Forward	9	558	0.0-20.3	20.3	644	0.0-35.4	35.2	
32	18	12229021-15990693	3,761,673	18	Forward	8	711	30.8-64.0	45.6	775	44.5-81.3	81.3	
33	3	1-3268600	3,268,600	3	Forward	7	488	0.0-13.1	0	543	0.0-22.3	22.3	
34	19	530650-3823253	3,292,604	19	Forward	7	561	10.3-41.6	41.6	598	11.7-55.4	19.1	
35	6	885756-4056779	3,171,024	6	Reverse	7	359	2.8-8.5	3	551	12.3-21.8	17.6	
36	8	1371557-4500840	3,129,284	8	Forward	7	599	6.6-22.3	18.6	730	7.4-28.5	22.9	
37	Un	5106262-7754674	2,648,413	21	Forward	6	1,721	11.7-12.0	11.8	1,187	10.1-12.6	11.5	
38	5	7878690-10536132	2,657,443	5	Forward	6	376	22.8-38.3	28.9	558	29.0-50.0	34.7	

39	7	2199899-4608158	2,408,260	7	Forward	5	360	25.5-43.7	27.7	513	37.1-61.8	37.1
40	14	13083936-15246461	2,162,526	14	Reverse	5	303	66.5-68.9	66.5	359	64.6-68.9	65
41	4	30452784-32632948	2,180,165	4	Forward	5	407	70.6-89.2	70.6	559	86.4-101.2	92.9
42	4	28356688-30451783	2,095,096	4	Forward	5	282	61.0-69.0	61	262	72.8-79.8	79.8
43	21	9495733-11398568	1,902,836	21	Forward	4	380	27.2-42.2	35.6	465	31.6-47.2	36.5
44	16	16169748-18115788	1,946,041	16	Reverse	4	698	56.0-59.4	56	785	58.7-78.3	78.3
45	15	14380836-16189872	1,809,037	15	Reverse	4	280	65.0-67.4	65.1	256	70.3-80.4	77.5
46	20	17951837-19732071	1,780,235	20	Forward	4	188	74.6-77.0	76.5	262	92.1-94.6	92.1
47	Un	7755675-9503854	1,748,180	7	Reverse	4	886	48.7-48.8	48.8	578	69.4-69.7	69.2
48	Un	9504855-11243840	1,738,986	5	Forward	4	222	1.5-6.0	1.5	354	3.8-12.3	9.2
49	11	621153-2204539	1,583,387	11	Reverse	4	212	0.9-1.5	0.9	204	3.0-6.8	4.8
50	3	15275813-16798506	1,522,694	3	Forward	4	173	51.6-72.9	51.6	268	63.4-76.8	63.4
51	13	1-1474264	1,474,264	13	Reverse	3	215	0.0-0.5	0.4	249	0.0-4.3	4.3
52	13	1475265-2824157	1,348,893	13	Forward	3	197	0.7-2.4	0.7	214	4.9-10.3	10.3
53	8	1-1370556	1,370,556	8	Forward	3	170	4.6-5.5	5	216	2.4-6.5	2.4
54	Un	11244841-12457396	1,212,556	5	Forward	3	127	0.0-0.6	0.6	160	0.0-2.0	2
55	7	928259-2198898	1,270,640	7	Forward	3	146	13.9-22.9	13.9	221	32.6-35.4	35.4
56	Un	12458397-13627317	1,168,921	8	Forward	3	140	0.0-3.5	3.5	141	0.0-0.3	0
57	13	18482172-19650590	1,168,419	13	Reverse	3	198	46.9-55.4	51.2	255	60.9-69.6	65.9
58	Un	13628318-14738452	1,110,135	10	Reverse	3	58	2.0-2.9	2	46	3.5	NA
59	10	14542210-15657440	1,115,231	10	Forward	3	145	63.4-65.2	65.2	171	83.0-85.7	85.7
60	12	17290519-18401067	1,110,549	12	Reverse	3	156	70.0-75.2	75.2	161	84.1-88.5	87
61	Un	14739453-15741363	1,001,911	6	Reverse	3	86	0.0-1.2	1.2	91	0.0-3.8	0
62	12	181397-1246382	1,064,986	12	Forward	3	192	17.8-26.3	17.8	173	19.9-33.1	33.1
63	2	1-965607	965,607	2	Reverse	2	208	0.2-0.8	0.2	126	1.6-2.6	2.6
64	12	1247383-2208411	961,029	12	Forward	2	181	41.4-43.7	43.7	159	49.2-50.6	50.6
65	4	1-1068876	1,068,876	4	Forward	3	130	0.0-2.8	2.8	129	0.0-3.6	3.6
66	6	1-884755	884,755	6	Reverse	2	137	1.7-2.6	1.7	187	6.8-10.9	6.8
67	Un	15742364-16635733	893,370	21	Forward	2	193	47.5-53.9	53.9	286	66	66
68	Un	16636734-17505905	869,172	12	Forward	2	156	37.7-40.3	37.7	150	45.6-47.8	45.6
69	Un	17506906-18320481	813,576	16	Reverse	2	59	68.5-68.8	68.5	48	89.4-89.6	89.4
70	12	16548615-17289518	740,904	12	Reverse	2	104	67.8-68.8	67.8	51	80.0-81.3	80
71	8	18122349-18945509	823,161	8	Forward	2	162	68.4-71.1	68.4	150	83.9-86.9	83.9
72	15	342542-1069868	727,327	15	Reverse	2	119	7.4-13.7	7.4	173	9.0-12.5	12.5
73	5	10998358-11741737	743,380	5	Reverse	2	137	48.9-53.9	48.9	228	65.3-70.9	70.9
74	Un	18321482-19003903	682,422	1	Forward	2	134	83.5	83.5	160	111.6-112.8	111.6
75	11	1-620152	620,152	11	Forward	2	71	0.8-1.3	1.3	88	1.7-2.4	1.7
76	Un	19004904-19601078	596,175	6	Reverse	2	36	2.9-3.4	2.9	12	NA	NA
77	9	17895191-18499799	604,609	9	Forward	2	118	54.1-56.7	56.7	151	72.7-76.6	76.6
78	3	14098027-14736947	638,921	3	Forward	2	100	34.0-34.9	34.9	124	44.1-46.2	44.1
79	11	15152914-15736674	583,761	11	Reverse	2	120	49.4-53.4	49.4	166	67.1-70.3	67.1
80	Un	19602079-20193314	591,236	11	Forward	2	62	0.0-0.2	0.2	44	0.0-0.8	0
81	3	14737948-15274812	536,865	3	Forward	2	119	42.5-45.1	42.5	98	53.2-57.6	NA
82	1	27586784-28185914	599,131	1	Reverse	2	78	94.3-95.6	94.3	75	121.1-121.5	121.5
83	1	1-540082	540,082	1	Reverse	2	39	0.0-0.7	0	63	0.0-0.1	0.3
84	Un	20194315-20757747	563,433	15	Forward	2	32	67.7-68.4	67.7	51	81.7-82.7	81.7
85	19	1-529649	529,649	19	Forward	2	60	0.0-1.4	0	95	0.0-0.8	0

86	7	1-539725	539,725	7	Reverse	2	69	2.6-3.6	3.6	76	11.5-13.3	13.3
87	9	19745084-20249479	504,396	9	Reverse	2	51	76.3-76.9	76.3	70	100.0-103.2	100
88	Un	20758748-21267600	508,853	14	Reverse	2	51	69.6	68.3	52	72.2-73.0	72.2
89	Un	21268601-21812334	543,734	17	Forward	2	26	0.0-0.3	0	56	0	0
90	Un	21813335-22322408	509,074	4	Reverse	2	14	107.3	107.3	19	133.0-135.4	133
91	9	18500800-19031248	530,449	9	Reverse	2	76	59.6-61.9	59.6	85	79.2-81.8	79.2
92	1	541083-1048102	507,020	1	Forward	2	76	1.4-2.9	2.9	111	0.5-0.9	0.3
93	5	10537133-10997357	460,225	5	Reverse	2	63	41.2-43.8	43.8	105	54.3-57.0	54.3
94	Un	22323409-22840795	517,387	21	Unknown	2	9	0	0	5	NA	10.1
95	Un	22841796-23342223	500,428	21	Forward	2	52	5.6-7.1	7.1	56	0	0
96	2	22376857-22828185	451,329	2	Reverse	2	36	79.8-79.9	79.8	24	95.0-95.6	95.6
97	1	1049103-1544349	495,247	1	Forward	2	72	5.2-6.6	6.6	94	3.6-5.3	5.3
98	Un	23343224-23754800	411,577	2	Reverse	2	43	0.0-0.4	0	55	0.0-0.4	0
99	Un	23755801-24176448	420,648	12	Reverse	2	66	28.2-29.6	29.6	106	37.6-40.1	37.6
100	11	16133022-16562300	429,279	11	Reverse	2	58	61.5-62.6	62.6	56	81.1-82.2	82.2
101	Un	24177449-24562071	384,623	10	Reverse	2	22	2.0-2.6	2.6	23	NA	1.8
102	2	22829186-23295652	466,467	2	Forward	2	69	78.2-79.7	79.7	77	94.4-95.3	95.3
103	11	15737675-16132021	394,347	11	Reverse	2	65	64.2-64.8	64.8	17	78.6	78.6
104	20	1-415733	415,733	20	Forward	2	99	8.9-9.4	8.9	61	5.2-6.0	6
105	9	19032249-19485083	452,835	9	Forward	2	48	63.7-64.6	63.7	76	85.1-86.9	86.9
106	Un	24563072-24936439	373,368	7	Forward	2	77	48.7-48.8	48.7	10	69.2-69.8	69.2
107	13	19651591-20070501	418,911	13	Reverse	2	37	66.6-68.0	68	59	76.2-79.0	79
108	Un	24937440-25286697	349,258	4	Reverse	2	16	58.7	59.1	36	67.4	67.4
109	17	1-409613	409,613	17	Reverse	2	110	0.6-2.0	2	93	1.8-3.0	3
110	15	1-341541	341,541	15	Reverse	2	33	0.0-0.3	0	42	0.0-3.6	0
111	Un	25287698-25631573	343,876	10	Forward	2	40	2.0-2.3	2	61	0.0-0.8	0.8
112	Un	25632574-25990228	357,655	5	Reverse	2	52	57.0-57.8	57	77	74.5-76.0	74.5
113	7	27614547-27937443	322,897	7	Forward	2	31	97.5-97.9	97.5	42	129.8	129.8
114	Un	25991229-26360520	369,292	12	Forward	2	34	0.0-0.5	0.5	47	0.0-1.1	1.1
115	Un	26361521-26705539	344,019	16	Reverse	2	47	62.4-66.3	62.4	78	84.0-85.3	85.3
116	14	1-345721	345,721	14	Reverse	2	19	0.0-0.4	0	47	1.4-2.0	1.4
117	6	16660651-17083675	423,025	6	Forward	2	14	60.8	60.5	31	82.2-83.0	83
118	7	540726-927258	386,533	7	Forward	2	47	5.1-8.2	5.1	56	15.4-18.2	18.2
119	Un	26706540-26996700	290,161	1	Forward	2	69	52.9-53.4	53.4	13	74.5	73.8
120	Un	26997701-27300803	303,103	3	Reverse	2	38	77.3-77.9	77.9	44	81.7-82.5	81.7
121	Un	27301804-27693698	391,895	4	Reverse	2	57	103.3-105.0	103.3	38	124.3-125.9	124.3
122	Un	27694699-27999486	304,788	20	Forward	2	67	11.1-12.8	11.1	65	9.3-13.3	9.3
123	1	27291229-27585783	294,555	1	Unknown	2	5	97.6	97.6	6	122.8	122.8
124	18	15991694-16282716	291,023	18	Unknown	2	5	NA	64	1	NA	81.3
125	5	11954056-12251397	297,342	5	Reverse	2	15	58.0-59.6	58.7	38	76.5-77.6	76.5
126	Un	28000487-28291363	290,877	21	Forward	2	43	63.3-64.0	63.3	43	76.3	76.3
127	21	11399569-11666335	266,767	21	Forward	2	40	59.7-62.2	62.2	49	73.7	73.7
128	Un	28292364-28543617	251,254	15	Reverse	2	30	64.6-65.2	64.7	5	NA	71.1
129	Un	28544618-28819305	274,688	7	Reverse	2	38	0.7-1.1	1.1	9	0.0-1.1	0
130	Un	28820306-29056818	236,513	10	Reverse	2	56	1.8-2.5	2.6	51	1.4-1.8	1.8
131	Un	29057819-29310115	252,297	7	Forward	2	26	0.0-1.5	1.5	25	8.8-9.6	9.6
132	Un	29311116-29570536	259,421	7	Forward	2	28	97.0-97.5	97	43	130.9	130.9

133	Un	29571537-29837943	266,407	13	Unknown	2	15	64.8	64.3	5	81.1	81.1	
134	Un	29838944-30096081	257,138	21	Unknown	2	6	NA	NA	6	1.9	1.9	
135	Un	30097082-30343148	246,067	3	Reverse	2	21	75.0-75.8	75	35	80.7-86.4	86.4	
136	17	410614-641510	230,897	17	Reverse	2	28	3.0-3.5	3.5	39	5	NA	
137	Un	30344149-30593893	249,745	20	Forward	2	37	0.0-10.4	10.4	23	0.0-7.0	7	
138	8	19133478-19368704	235,227	8	Reverse	2	20	73.2	73.2	49	89.2-89.3	89.2	
139	Un	30594894-30817804	222,911	12	Reverse	2	19	1.9-3.4	3.4	29	0.9-1.7	1.7	
140	6	16418535-16659650	241,116	6	Unknown	2	25	60.1	60.5	43	81.1	81.1	
141	Un	30818805-31038364	219,560	12	Unknown	2	1	NA	1.9	2	NA	0.9	
142	5	11742738-11953055	210,318	5	Forward	2	24	58.4-58.7	58.7	24	76.5-77.0	76.5	
143	20	593103-833141	240,039	20	Forward	2	33	15.2-17.6	15.2	37	21.1	21.1	
144	21	1-297070	297,070	21	Unknown	2	4	8.9	8.9	6	2.7	2.7	
145	Un	31039365-31334864	295,500	21	Unknown	2	1	NA	5.6	1	NA	1.9	
146	Un	31335865-31560395	224,531	21	Reverse	2	13	69	67.7	21	77.4-77.6	77.4	
147	Un	31561396-31777177	215,782	21	Unknown	2	5	8.5	8.5	2	NA	10.1	
148	Un	31778178-31975086	196,909	12	Forward	2	21	76.0-76.9	76.9	28	91.6-94.3	91.6	
149	Un	31976087-32163437	187,351	10	Forward	2	159	62.0-62.9	62.2	152	84.1-84.2	84.3	
150	Un	32164438-32354918	190,481	13	Reverse	2	17	65.8-65.9	65.8	32	80.0-80.4	80	
151	Un	32355919-32547295	191,377	11	Forward	2	25	44.2	44.2	61	63.5-65.3	65.3	
152	Un	32548296-32814531	266,236	4	Reverse	2	38	106.7	106.7	38	127.4-128.2	127.4	
153	8	18946510-19132477	185,968	8	Reverse	2	1	NA	73.2	57	89.7-91.0	89.7	
155	Un	33000773-33191444	190,672	21	Unknown	2	5	NA	11.8	0	NA	NA	
156	Un	33192445-33362665	170,221	6	Forward	2	19	60.5-61.0	60.5	13	83.7	83.7	
157	Un	33363666-33534841	171,176	20	Forward	2	21	9.2-9.7	9.2	19	1.1-5.5	0	
158	12	1-180396	180,396	12	Forward	2	36	11.4-12.8	12.8	54	10.4	NA	
159	Un	33535842-33702692	166,851	15	Forward	2	24	2.8	2.8	16	5.0-5.3	5	
160	Un	33703693-33861776	158,084	*16/10*	*Unknown*	*2*	*7*	*NA*	*59.4*	*6*	*NA*	*0.8*	a
161	Un	33862777-34020958	158,182	3	Forward	2	15	78.3-78.9	78.9	6	NA	86.4	
162	Un	34021959-34197025	175,067	21	Unknown	2	5	NA	7.1	0	NA	NA	
163	Un	34198026-34359936	161,911	19	Reverse	2	37	2.9	2.9	48	3.3-5.2	3.3	
164	Un	34360937-34619011	258,075	21	Unknown	2	1	NA	5.6	0	NA	NA	
165	Un	34620012-34788385	168,374	1	Forward	2	34	11.1-12.5	11.1	25	12.1-16.5	16.5	
167	Un	34987570-35142483	154,914	11	Unknown	2	9	0.4	0.4	1	NA	NA	
168	Un	35143484-35297961	154,478	14	Reverse	2	36	2.6-3.7	2.6	21	3.1-3.7	3.1	
169	Un	35298962-35453231	154,270	3	Forward	2	20	39.0-40.3	40.3	36	52	52	
170	20	416734-592102	175,369	20	Forward	2	21	7.8-8.3	7.8	36	5.7-5.8	5.8	
171	Un	35454232-35640608	186,377	9	Unknown	2	7	NA	59.6	3	NA	79.2	
173	Un	35817148-36026950	209,803	9	Unknown	2	7	NA	69.4	9	NA	94.7	
174	17	766878-935213	168,336	17	Forward	2	20	5.0-5.6	5.6	25	9.9	9.9	
175	Un	36027951-36199373	171,423	1	Forward	2	25	9.4-10.5	10.5	44	8.6-9.4	8.6	
176	Un	36200374-36342913	142,540	14	Unknown	2	5	NA	0	7	0	0	
177	Un	36343914-36492910	148,997	9	Forward	2	29	71.7-72.1	72.1	30	96.8-98.8	98.8	
180	Un	36787135-36924936	137,802	12	Unknown	2	12	6.1	6.1	15	5.7	5.7	
181	Un	36925937-37061398	135,462	16	Forward	2	23	67.2-67.8	67.8	31	87.8	86.5	
182	Un	37062399-37196909	134,511	21	Reverse	2	8	67.7	67.7	13	78.2-79.1	79.1	
183	9	19486084-19607294	121,211	9	Reverse	2	37	65.8-66.3	65.8	26	88.9-90.0	88.9	
184	Un	37197910-37345605	147,696	15	Reverse	2	51	3.8-4.2	4.2	40	6.2-6.5	6.5	

185	Un	37346606-37498022	151,417	7	Unknown	2	8	NA	13.9	0	NA	NA
186	Un	37499023-37637380	138,358	1	Unknown	2	3	NA	0	10	0.3	0.3
188	Un	37754495-37875713	121,219	21	Unknown	2	1	NA	NA	4	NA	10.1
189	9	19627166-19744083	116,918	9	Forward	2	25	69.4	74.6	15	92.7-101.2	100
190	11	16563301-16706052	142,752	11	Unknown	2	6	62.8	62.6	11	84.1	78.6
191	17	642511-765877	123,367	17	Unknown	2	14	4.2	4.2	18	5.7	5.7
193	Un	38013290-38114339	101,050	7	Unknown	2	3	NA	8.2	1	NA	NA
194	Un	38115340-38232624	117,285	21	Unknown	2	1	NA	0	0	NA	NA
195	Un	38233625-38380945	147,321	12	Reverse	2	19	5.3-6.5	6.5	24	3	3
196	Un	38381946-38480572	98,627	14	Unknown	1	12	NA	0	15	NA	2
197	Un	38481573-38597001	115,429	14	Forward	2	10	0.7-0.9	0.9	19	2.4	2.4
198	Un	38598002-38706708	108,707	9	Unknown	2	3	NA	NA	7	94.7	94.7
199	Un	38707709-38805661	97,953	4	Unknown	1	20	NA	89.2	9	NA	NA
200	Un	38806662-38907936	101,275	13	Unknown	2	6	65.5	65.5	8	79.6	79
201	Un	38908937-39051539	142,603	3	Unknown	2	2	NA	51.6	5	61	61
202	Un	39052540-39162288	109,749	16	Reverse	2	13	70.8	67.8	12	86.5-87.4	87.4
203	Un	39163289-39271365	108,077	21	Unknown	2	0	NA	NA	2	NA	10.1
204	Un	39272366-39363831	91,466	1	Unknown	1	17	NA	5.2	14	NA	0.3
205	Un	39364832-39491281	126,450	9	Unknown	2	6	NA	66.3	4	NA	NA
206	Un	39492282-39591840	99,559	4	Unknown	1	20	NA	103.3	21	NA	NA
208	Un	39711137-39820485	109,349	10	Reverse	2	63	61.0-62.5	61	52	84.4-85.1	85.1
210	Un	39910623-39994605	83,983	1	Unknown	1	6	NA	95.6	7	NA	121.1
211	Un	39995606-40077297	81,692	8	Unknown	1	18	NA	71.1	14	NA	NA
213	Un	40165280-40250677	85,398	21	Unknown	1	9	NA	64	11	NA	77.4
214	Un	40251678-40339421	87,744	21	Unknown	1	6	NA	64	10	NA	77.4
215	Un	40340422-40434995	94,574	21	Unknown	1	1	NA	8.9	0	NA	NA
216	Un	40435996-40530711	94,716	6	Unknown	1	12	NA	60.1	18	NA	74.4
217	Un	40531712-40613024	81,313	21	Unknown	1	2	NA	11.5	0	NA	NA
218	Un	40614025-40703418	89,394	8	Unknown	1	13	NA	71.1	19	NA	89.2
219	Un	40704419-40783668	79,250	3	Unknown	1	5	NA	75	13	NA	NA
220	Un	40784669-40904766	120,098	13	Unknown	2	10	64.3	64.3	1	NA	79
221	Un	40905767-40979726	73,960	4	Unknown	1	3	NA	58.1	6	NA	67.5
222	4	1069877-1173334	103,458	4	Unknown	2	14	4.2	4.2	20	6	6
224	Un	41062695-41270847	208,153	19	Unknown	2	0	NA	NA	3	47.9	47.9
225	Un	41271848-41362991	91,144	13	Unknown	1	4	NA	65.8	13	NA	80
227	Un	41490409-41590171	99,763	21	Unknown	1	1	NA	8.5	2	NA	NA
229	Un	41659832-41725083	65,252	20	Unknown	1	11	NA	11.1	8	NA	9.3
231	Un	41794577-41866531	71,955	4	Unknown	1	2	NA	107.3	5	NA	127.4
232	Un	41867532-42039735	172,204	9	Unknown	2	2	NA	66.3	0	NA	NA
234	Un	42115228-42185738	70,511	21	Unknown	1	2	NA	11.8	0	NA	NA
235	Un	42186739-42250670	63,932	2	Unknown	1	10	NA	78.2	19	NA	94.4
237	Un	42315881-42383608	67,728	12	Unknown	1	3	NA	0	5	NA	1.7
238	Un	42384609-42446850	62,242	9	Unknown	1	7	NA	71.7	17	NA	94.7
239	Un	42447851-42509289	61,439	12	Unknown	1	10	NA	12.8	12	NA	NA
240	Un	42510290-42576502	66,213	16	Unknown	1	5	NA	68.5	10	NA	89.4
242	Un	42638668-42734078	95,411	21	Unknown	1	0	NA	NA	1	NA	1.9
243	Un	42735079-42812442	77,364	13	Unknown	1	9	NA	0	9	NA	NA

244	Un	42813443-42915923	102,481	9	Unknown	2	7	74.6	74.6	15	100.3	100	
246	Un	42979616-43037963	58,348	10	Unknown	1	86	NA	62.5	82	NA	84.1	
247	Un	43038964-43093304	54,341	15	Unknown	1	12	NA	0.3	3	NA	NA	
248	Un	43094305-43149449	55,145	16	Unknown	1	0	NA	NA	4	NA	46.8	
249	Un	43150450-43208946	58,497	15	Unknown	1	2	NA	0.3	7	NA	0	
251	Un	43283583-43336528	52,946	9	Unknown	1	1	NA	69.4	0	NA	NA	
253	Un	43389470-43454658	65,189	11	Unknown	1	2	NA	11.4	2	NA	NA	
256	Un	43622280-43680027	57,748	5	Unknown	1	0	NA	NA	1	NA	12.9	
257	21	11667336-11717487	50,152	21	Unknown	1	2	NA	67.7	0	NA	NA	
259	Un	43742337-43835441	93,105	1	Unknown	1	5	NA	83.5	12	NA	NA	
260	Un	43836442-43892821	56,380	21	Unknown	1	1	NA	11.5	0	NA	NA	
261	Un	43893822-43948169	54,348	14	Unknown	1	2	NA	6.7	0	NA	NA	
265	Un	44137454-44209165	71,712	21	Unknown	1	0	NA	NA	2	NA	1.9	
266	Un	44210166-44266666	56,501	21	Unknown	1	1	NA	7.1	0	NA	NA	
267	Un	44267667-44325426	57,760	9	Unknown	1	3	NA	64.6	6	NA	NA	
268	Un	44326427-44386154	59,728	14	Unknown	1	1	NA	0.7	0	NA	NA	
269	Un	44387155-44431490	44,336	18	Unknown	1	4	NA	64	2	NA	NA	
270	Un	44432491-44475220	42,730	4	Unknown	1	6	NA	107.3	2	NA	NA	
273	Un	44606402-44648496	42,095	7	Unknown	1	9	NA	95.1	15	NA	130.9	
276	Un	44736933-44778502	41,570	12	Unknown	1	13	NA	11.4	7	NA	NA	
277	Un	44779503-44820394	40,892	6	Unknown	1	5	NA	60.1	12	NA	81.1	
279	Un	44891839-44932111	40,273	17	Unknown	1	5	NA	0.3	15	NA	0	
280	Un	44933112-44993620	60,509	4	Unknown	1	9	NA	105	11	NA	NA	
281	Un	44994621-45063811	69,191	4	Unknown	1	10	NA	106.7	7	NA	125.9	
282	Un	45064812-45104696	39,885	21	Unknown	1	0	NA	NA	1	NA	10.1	
283	Un	45105697-45161722	56,026	21	Unknown	1	0	NA	NA	2	NA	1.9	
285	Un	45203822-45244729	40,908	14	Unknown	1	3	NA	0.9	1	NA	2	
286	Un	45245730-45285318	39,589	12	Unknown	1	0	NA	NA	3	NA	52.4	
288	Un	45349565-45388744	39,180	21	Unknown	1	1	NA	0	0	NA	NA	
291	Un	45511903-45550564	38,662	21	Unknown	1	2	NA	7.1	1	NA	NA	
293	Un	45590871-45642025	51,155	14	Unknown	1	3	NA	0.7	0	NA	NA	
294	Un	45643026-45696553	53,528	21	Unknown	1	1	NA	8.5	0	NA	NA	
296	Un	45736233-45794993	58,761	9	Unknown	1	18	NA	64.6	5	NA	NA	
299	Un	45870018-45905559	35,542	10	Unknown	1	2	NA	44.9	3	NA	54.7	
300	Un	45906560-45960251	53,692	1	Unknown	1	5	NA	83.5	6	NA	NA	
303	Un	46068541-46103086	34,546	8	Unknown	1	1	NA	16.3	0	NA	NA	
304	Un	46104087-46138840	34,754	20	Unknown	1	1	NA	53.2	0	NA	NA	
306	Un	46175505-46231026	55,522	9	Unknown	1	2	NA	63.7	2	NA	86.9	
307	Un	46232027-46266711	34,685	12	Unknown	1	5	NA	6.1	5	NA	1.7	
309	Un	46327163-46361761	34,599	21	Unknown	1	7	NA	NA	6	NA	13.4	
311	Un	46400029-46460590	60,562	21	Unknown	1	2	NA	11.8	0	NA	NA	
313	Un	46514490-46567505	53,016	*20/12*	*Unknown*	*1*	*1*	*NA*	*52.7*	*1*	*NA*	*33.1*	b
314	Un	46568506-46603289	34,784	15	Unknown	1	2	NA	2.8	7	NA	3.6	
316	Un	46638551-46670753	32,203	7	Unknown	1	1	NA	NA	1	NA	0	
318	Un	46725951-46789913	63,963	21	Unknown	1	1	NA	0	0	NA	NA	
321	Un	46876408-46971532	95,125	20	Unknown	1	1	NA	11.1	0	NA	NA	
323	Un	47006411-47059861	53,451	13	Unknown	1	2	NA	64.3	3	NA	81.1	

324	Un	47060862-47091664	30,803	7	Unknown	1	2	NA	97.9	4	NA	130.9
326	Un	47124039-47177501	53,463	14	Unknown	1	0	NA	NA	2	NA	2
327	Un	47178502-47208678	30,177	20	Unknown	1	5	NA	NA	6	NA	60.3
328	Un	47209679-47269348	59,670	8	Unknown	1	10	NA	24	0	NA	NA
332	Un	47362639-47393753	31,115	9	Unknown	1	1	NA	20.9	6	NA	38.9
334	Un	47425274-47482086	56,813	21	Unknown	1	0	NA	NA	1	NA	10.1
338	Un	47611169-47662141	50,973	13	Unknown	1	2	NA	64.8	12	NA	79.6
339	Un	47663142-47722178	59,037	14	Unknown	1	4	NA	6.7	2	NA	NA
340	Un	47723179-47751104	27,926	9	Unknown	1	1	NA	66.3	0	NA	NA
342	Un	47804064-47866166	62,103	21	Unknown	1	1	NA	0	0	NA	NA
343	Un	47867167-47895531	28,365	21	Unknown	1	8	NA	63.3	3	NA	77.4
346	Un	47959095-47985867	26,773	15	Unknown	1	1	NA	51.5	1	NA	52.1
349	Un	48070254-48096867	26,614	13	Unknown	1	9	NA	68	13	NA	76.2
350	Un	48097868-48156594	58,727	13	Unknown	1	3	NA	NA	3	NA	40.4
351	Un	48157595-48184126	26,532	21	Unknown	1	0	NA	NA	1	NA	2.7
352	Un	48185127-48236169	51,043	21	Unknown	1	1	NA	0	0	NA	NA
355	Un	48293502-48318618	25,117	21	Unknown	1	12	NA	64	5	NA	77.4
357	Un	48366565-48391571	25,007	8	Unknown	1	0	NA	NA	4	NA	86.9
358	Un	48392572-48417944	25,373	11	Unknown	1	4	NA	1	2	NA	NA
359	Un	48418945-48443627	24,683	1	Unknown	1	1	NA	67	5	NA	NA
363	Un	48545426-48569683	24,258	14	Unknown	1	1	NA	0.7	0	NA	NA
365	Un	48595857-48620102	24,246	12	Unknown	1	1	NA	NA	3	NA	1.1
368	Un	48671136-48714869	43,734	21	Unknown	1	2	NA	8.9	0	NA	NA
369	Un	48715870-48740052	24,183	9	Unknown	1	5	NA	65.8	4	NA	92.7
370	Un	48741053-48764711	23,659	21	Unknown	1	1	NA	11.5	0	NA	NA
380	Un	48990316-49013826	23,511	12	Unknown	1	0	NA	NA	1	NA	0
381	Un	49014827-49037708	22,882	13	Unknown	1	7	NA	55.4	4	NA	69.6
382	Un	49038709-49063862	25,154	13	Unknown	1	9	NA	46.9	5	NA	60.9
385	Un	49112679-49135318	22,640	14	Unknown	1	2	NA	6.7	0	NA	NA
387	Un	49159782-49207533	47,752	1	Unknown	1	1	NA	63.3	0	NA	NA
391	Un	49278818-49300531	21,714	16	Unknown	1	4	NA	0	0	NA	NA
393	Un	49324284-49345924	21,641	1	Unknown	1	7	NA	6.6	6	NA	5.3
395	Un	49373502-49395098	21,597	13	Unknown	1	2	NA	64.3	0	NA	NA
396	Un	49396099-49418099	22,001	12	Unknown	1	0	NA	NA	2	NA	45.6
397	Un	49419100-49440577	21,478	21	Unknown	1	4	NA	64	3	NA	77.6
398	Un	49441578-49466932	25,355	9	Unknown	1	1	NA	65.8	0	NA	NA
402	Un	49537108-49558127	21,020	13	Unknown	1	0	NA	NA	1	NA	81.1
404	Un	49581057-49601996	20,940	8	Unknown	1	0	NA	NA	7	NA	83.9
405	Un	49602997-49623832	20,836	19	Unknown	1	3	NA	0	7	NA	0
413	Un	49781774-49802315	20,542	7	Unknown	1	3	NA	5.1	2	NA	0
417	Un	49868525-49888818	20,294	7	Unknown	1	3	NA	8.2	0	NA	NA
419	Un	49911083-49931290	20,208	8	Unknown	1	1	NA	20.3	3	NA	NA
422	Un	49976658-49997270	20,613	6	Unknown	1	3	NA	60.5	11	NA	82.2
423	Un	49998271-50018017	19,747	21	Unknown	1	4	NA	8.5	0	NA	NA
424	Un	50019018-50038855	19,838	9	Unknown	1	5	NA	64.6	2	NA	85.1
425	Un	50039856-50061674	21,819	21	Unknown	1	2	NA	67.7	1	NA	77.4
427	Un	50083395-50103044	19,650	14	Unknown	1	0	NA	NA	1	NA	3.7

433	Un	50208623-50230651	22,029	6	Unknown	1	1	NA	61	2	NA	82.2
436	Un	50271895-50290710	18,816	21	Unknown	1	3	NA	0	1	NA	NA
439	Un	50331895-50354498	22,604	4	Unknown	1	1	NA	106.7	1	NA	128.2
440	Un	50355499-50373966	18,468	13	Unknown	1	2	NA	68	2	NA	NA
442	Un	50394398-50412766	18,369	21	Unknown	1	1	NA	NA	3	NA	77.4
443	Un	50413767-50431847	18,081	8	Unknown	1	2	NA	68.4	2	NA	77.2
446	Un	50470946-50488940	17,995	7	Unknown	1	1	NA	2.6	1	NA	11.5
447	9	19608295-19626165	17,871	9	Unknown	1	2	NA	74.6	3	NA	98.8
453	Un	50620164-50637765	17,602	7	Unknown	1	7	NA	1.1	1	NA	NA
455	Un	50657180-50674591	17,412	12	Unknown	1	3	NA	46	3	NA	50.6
456	Un	50675592-50692994	17,403	21	Unknown	1	1	NA	NA	1	NA	1.9
468	Un	50899409-50916489	17,081	8	Unknown	1	1	NA	55.3	1	NA	77.2
470	Un	50935545-50952590	17,046	13	Unknown	1	0	NA	NA	1	NA	40.4
472	Un	50972612-50989710	17,099	10	Unknown	1	1	NA	63.4	3	NA	83
473	Un	50990711-51007659	16,949	4	Unknown	1	4	NA	NA	1	NA	50.4
475	Un	51026996-51043724	16,729	7	Unknown	1	3	NA	2.6	4	NA	9.6
478	Un	51115257-51132144	16,888	14	Unknown	1	0	NA	NA	1	NA	2
482	Un	51188528-51204986	16,459	16	Unknown	1	7	NA	38.1	1	NA	46.8
486	Un	51258562-51274863	16,302	21	Unknown	1	1	NA	7.1	1	NA	10.1
497	Un	51451746-51467660	15,915	8	Unknown	1	4	NA	28.3	2	NA	NA
501	Un	51519186-51536345	17,160	20	Unknown	1	1	NA	NA	3	NA	9.3
509	Un	51655187-51671091	15,905	21	Unknown	1	1	NA	0	0	NA	NA
511	Un	51688524-51705859	17,336	20	Unknown	1	1	NA	7.8	1	NA	13.3
516	Un	51775211-51790509	15,299	7	Unknown	1	0	NA	NA	1	NA	63.7
517	Un	51791510-51806797	15,288	15	Unknown	1	7	NA	3.8	10	NA	6.2
520	Un	51840909-51856407	15,499	13	Unknown	1	1	NA	65.5	6	NA	NA
521	Un	51857408-51872569	15,162	4	Unknown	1	0	NA	NA	1	NA	127.4
522	Un	51873570-51889777	16,208	9	Unknown	1	4	NA	74.6	5	NA	100
524	Un	51906862-51921932	15,071	6	Unknown	1	2	NA	60.5	3	NA	82.2
529	Un	51988383-52003350	14,968	20	Unknown	1	3	NA	11.1	2	NA	7
542	Un	52201296-52216041	14,746	17	Unknown	1	1	NA	0	1	NA	0
545	Un	52250580-52265215	14,636	4	Unknown	1	2	NA	1.3	2	NA	NA
550	Un	52328471-52342789	14,319	21	Unknown	1	3	NA	64	6	NA	77.6
559	Un	52499012-52513129	14,118	21	Unknown	1	0	NA	NA	1	NA	13.4
560	Un	52514130-52528076	13,947	4	Unknown	1	4	NA	1.3	0	NA	NA
562	Un	52543993-52561524	17,532	8	Unknown	1	5	NA	73.2	1	NA	NA
563	Un	52562525-52576412	13,888	11	Unknown	1	4	NA	NA	5	NA	17.8
564	Un	52577413-52591281	13,869	19	Unknown	1	9	NA	46.1	4	NA	60.5
578	Un	52789509-52803384	13,876	12	Unknown	1	0	NA	NA	3	NA	47.8
579	Un	52804385-52821172	16,788	13	Unknown	1	4	NA	18.8	1	NA	30.1
587	Un	52923191-52936646	13,456	3	Unknown	1	4	NA	72.9	2	NA	NA
593	Un	53009594-53022925	13,332	1	Unknown	1	4	NA	97.6	4	NA	122.8
599	Un	53094667-53107818	13,152	11	Unknown	1	2	NA	13.5	1	NA	25.8
600	Un	53108819-53121857	13,039	21	Unknown	1	0	NA	NA	5	NA	77.4
601	Un	53122858-53135889	13,032	15	Unknown	1	6	NA	51.5	6	NA	55.4
604	Un	53165806-53178953	13,148	9	Unknown	1	1	NA	69.4	2	NA	94.7
605	Un	53179954-53193336	13,383	4	Unknown	1	3	NA	105	3	NA	127.4

613	Un	53293363-53306362	13,000	21	Unknown	1	1	NA	0	0	NA	NA
615	Un	53321160-53333933	12,774	2	Unknown	1	2	NA	8.6	1	NA	21.4
618	Un	53362880-53377982	15,103	9	Unknown	1	1	NA	61.9	0	NA	NA
620	Un	53395873-53408554	12,682	2	Unknown	1	2	NA	18.5	0	NA	NA
621	Un	53409555-53422235	12,681	12	Unknown	1	1	NA	17.8	1	NA	19.9
625	Un	53464341-53476958	12,618	21	Unknown	1	3	NA	10.9	0	NA	NA
632	Un	53561747-53574175	12,429	4	Unknown	1	0	NA	NA	2	NA	51
637	Un	53628730-53641078	12,349	9	Unknown	1	1	NA	64.6	0	NA	NA
642	Un	53696395-53708682	12,288	10	Unknown	1	1	NA	61	0	NA	NA
664	Un	53988298-54001569	13,272	9	Unknown	1	0	NA	NA	5	NA	100.3
676	13	20071502-20083130	11,629	13	Unknown	1	1	NA	66.6	1	NA	79
680	Un	54210990-54222567	11,578	9	Unknown	1	0	NA	NA	3	NA	100
681	Un	54223568-54235116	11,549	13	Unknown	1	2	NA	64.3	4	NA	76.2
682	Un	54236117-54248584	12,468	21	Unknown	1	1	NA	8.5	0	NA	NA
683	Un	54249585-54261089	11,505	1	Unknown	1	0	NA	NA	1	NA	121.1
689	Un	54325891-54337328	11,438	5	Unknown	1	2	NA	0.2	4	NA	0.4
691	Un	54350689-54362032	11,344	2	Unknown	1	1	NA	72.4	3	NA	88.6
695	Un	54400542-54411831	11,290	15	Unknown	1	0	NA	NA	2	NA	81.7
697	Un	54425236-54436497	11,262	9	Unknown	1	3	NA	63.7	0	NA	NA
698	Un	54437498-54451049	13,552	13	Unknown	1	0	NA	NA	1	NA	20.2
707	Un	54551394-54562409	11,016	15	Unknown	1	3	NA	0.3	2	NA	5
713	Un	54623353-54634314	10,962	7	Unknown	1	0	NA	NA	2	NA	32.6
717	Un	54671166-54682090	10,925	13	Unknown	1	1	NA	64.8	0	NA	NA
718	Un	54683091-54694002	10,912	10	Unknown	1	13	NA	62.2	4	NA	84.1
719	Un	54695003-54705978	10,976	16	Unknown	1	0	NA	NA	2	NA	84
727	Un	54789801-54800560	10,760	1	Unknown	1	1	NA	10.5	6	NA	8.6
729	Un	54813406-54824116	10,711	5	Unknown	1	1	NA	13.5	1	NA	18.9
730	Un	54825117-54835821	10,705	15	Unknown	1	3	NA	67.4	0	NA	NA
732	Un	54848788-54859473	10,686	11	Unknown	1	1	NA	0	0	NA	NA
735	Un	54884944-54895928	10,985	15	Unknown	1	0	NA	NA	3	NA	80.4
739	Un	54935473-54946056	10,584	14	Unknown	1	0	NA	NA	1	NA	5.6
740	Un	54947057-54957634	10,578	9	Unknown	1	1	NA	74.6	2	NA	100
741	Un	54958635-54969306	10,672	1	Unknown	1	1	NA	5.2	4	NA	0.3
742	Un	54970307-54980780	10,474	10	Unknown	1	2	NA	0	3	NA	6.1
745	Un	55005167-55015604	10,438	15	Unknown	1	0	NA	NA	1	NA	6.2
748	Un	55039432-55049815	10,384	21	Unknown	1	1	NA	11.6	0	NA	NA
749	Un	55050816-55061334	10,519	21	Unknown	1	1	NA	0	0	NA	NA
751	Un	55073677-55084012	10,336	15	Unknown	1	2	NA	3.8	0	NA	NA
757	Un	55142438-55152645	10,208	9	Unknown	1	2	NA	74.6	3	NA	100
758	Un	55153646-55163846	10,201	16	Unknown	1	0	NA	NA	1	NA	0
761	Un	55187184-55197327	10,144	4	Unknown	1	0	NA	NA	1	NA	50.4
762	Un	55198328-55208455	10,128	14	Unknown	1	1	NA	0.4	0	NA	NA
764	Un	55220561-55230649	10,089	21	Unknown	1	1	NA	11.8	2	NA	11.5
768	Un	55264836-55274873	10,038	16	Unknown	1	3	NA	67.8	0	NA	NA
786	Un	55471340-55481338	9,999	13	Unknown	1	1	NA	64.8	0	NA	NA
793	Un	55550131-55559845	9,715	18	Unknown	1	1	NA	26.1	1	NA	NA
796	Un	55583639-55594378	10,740	6	Unknown	1	0	NA	NA	1	NA	81.1

806	Un	55692216-55701794	9,579	4	Unknown	1	1	NA	89.2	1	NA	NA
812	Un	55794638-55804183	9,546	8	Unknown	1	0	NA	NA	4	NA	77.2
814	Un	55816024-55825525	9,502	12	Unknown	1	2	NA	12.8	3	NA	10.4
822	Un	55903920-55913364	9,445	13	Unknown	1	5	NA	51.2	1	NA	65.9
829	Un	55977368-55986733	9,366	12	Unknown	1	1	NA	55.5	0	NA	NA
830	Un	55987734-55997092	9,359	15	Unknown	1	3	NA	4.2	1	NA	9
834	Un	56029278-56038557	9,280	13	Unknown	1	0	NA	NA	2	NA	79
837	Un	56060876-56070125	9,250	7	Unknown	1	2	NA	45.4	2	NA	63.7
842	Un	56113504-56122641	9,138	8	Unknown	1	1	NA	73.2	4	NA	89.2
843	Un	56123642-56132773	9,132	2	Unknown	1	0	NA	NA	6	NA	38.1
857	Un	56266790-56275772	8,983	16	Unknown	1	0	NA	NA	2	NA	40.7
861	Un	56306684-56315644	8,961	9	Unknown	1	0	NA	NA	3	NA	94.7
868	Un	56376232-56385129	8,898	1	Unknown	1	1	NA	94.3	1	NA	111.6
869	Un	56386130-56396944	10,815	1	Unknown	1	1	NA	6.6	1	NA	3.6
872	Un	56417956-56426818	8,863	20	Unknown	1	0	NA	NA	1	NA	5.7
877	Un	56470671-56479449	8,779	6	Unknown	1	1	NA	60.1	0	NA	NA
882	Un	56519794-56528503	8,710	9	Unknown	1	1	NA	5.6	9	NA	NA
889	Un	56590570-56599187	8,618	1	Unknown	1	1	NA	97.6	2	NA	121.1
891	Un	56609799-56618406	8,608	8	Unknown	1	2	NA	71.1	0	NA	NA
894	Un	56638581-56647142	8,562	21	Unknown	1	0	NA	NA	2	NA	17.4
906	Un	56755315-56763764	8,450	9	Unknown	1	0	NA	NA	1	NA	90
913	Un	56823823-56832224	8,402	21	Unknown	1	3	NA	7.1	1	NA	2.7
926	Un	56945457-56953670	8,214	3	Unknown	1	0	NA	NA	7	NA	46.2
930	Un	56982433-56990599	8,167	13	Unknown	1	0	NA	NA	4	NA	76.2
931	Un	56991600-56999761	8,162	4	Unknown	1	1	NA	0	0	NA	NA
935	Un	57028453-57036596	8,144	3	Unknown	1	3	NA	14.8	0	NA	NA
936	Un	57037597-57045738	8,142	1	Unknown	1	0	NA	NA	1	NA	54.7
937	Un	57046739-57054879	8,141	9	Unknown	1	0	NA	NA	1	NA	90
938	Un	57055880-57064019	8,140	8	Unknown	1	0	NA	NA	2	NA	89.7
944	Un	57110641-57118739	8,099	2	Unknown	1	1	NA	79.7	0	NA	NA
953	Un	57192712-57200726	8,015	7	Unknown	1	1	NA	95.1	1	NA	129.8
954	Un	57201727-57209734	8,008	7	Unknown	1	0	NA	NA	1	NA	32.6
957	Un	57228709-57236667	7,959	14	Unknown	1	0	NA	NA	2	NA	52.7
962	Un	57275801-57283719	7,919	13	Unknown	1	0	NA	NA	5	NA	60.9
968	15	16190873-16198764	7,892	15	Unknown	1	0	NA	NA	1	NA	80.4
977	Un	57406596-57414431	7,836	17	Unknown	1	0	NA	NA	1	NA	1.8
978	Un	57415432-57423259	7,828	4	Unknown	1	0	NA	NA	2	NA	62.8
986	Un	57486141-57493844	7,704	21	Unknown	1	2	NA	0	2	NA	11.2
994	Un	57560167-57567818	7,652	13	Unknown	1	2	NA	64.3	4	NA	76.2
996	Un	57577468-57585103	7,636	21	Unknown	1	1	NA	8.5	0	NA	NA
999	Un	57603360-57610962	7,603	10	Unknown	1	21	NA	62.2	14	NA	84.4
1001	Un	57620562-57628158	7,597	20	Unknown	1	1	NA	49.3	0	NA	NA
1008	Un	57684233-57691769	7,537	12	Unknown	1	1	NA	43.7	2	NA	50.6
1009	Un	57692770-57700288	7,519	13	Unknown	1	1	NA	64.3	5	NA	76.2
1010	Un	57701289-57710250	8,962	21	Unknown	1	0	NA	NA	1	NA	10.1
1014	Un	57737580-57745044	7,465	20	Unknown	1	2	NA	10.4	6	NA	5.8
1019	Un	57780348-57787785	7,438	9	Unknown	1	0	NA	NA	2	NA	40.4

1020	Un	57788786-57796210	7,425	4	Unknown	1	1	NA	107.3	0	NA	NA
1023	Un	57814059-57821464	7,406	3	Unknown	1	0	NA	NA	1	NA	0
1029	Un	57866552-57873922	7,371	15	Unknown	1	1	NA	2.8	0	NA	NA
1041	Un	57966706-57974006	7,301	21	Unknown	1	1	NA	NA	1	NA	1.9
1049	Un	58033452-58040717	7,266	4	Unknown	1	1	NA	106.7	2	NA	127.4
1050	Un	58041718-58048980	7,263	9	Unknown	1	0	NA	NA	3	NA	103.2
1058	Un	58107766-58114972	7,207	8	Unknown	1	2	NA	60.8	1	NA	77.2
1060	Un	58124278-58131480	7,203	11	Unknown	1	0	NA	NA	1	NA	27.8
1067	Un	58181913-58189096	7,184	19	Unknown	1	1	NA	10.3	0	NA	NA
1070	Un	58207785-58214942	7,158	1	Unknown	1	0	NA	NA	5	NA	8.6
1073	Un	58232223-58239350	7,128	4	Unknown	1	0	NA	NA	2	NA	3
1075	Un	58248464-58255667	7,204	8	Unknown	1	1	NA	NA	8	NA	67.5
1082	Un	58305532-58313200	7,669	16	Unknown	1	3	NA	30.6	0	NA	NA
1087	Un	58346812-58353833	7,022	8	Unknown	1	1	NA	37.1	0	NA	NA
1088	Un	58354834-58361851	7,018	1	Unknown	1	2	NA	1.4	0	NA	NA
1090	Un	58370965-58377974	7,010	13	Unknown	1	1	NA	39.2	0	NA	NA
1093	Un	58394962-58401921	6,960	10	Unknown	1	0	NA	NA	1	NA	83
1099	Un	58442928-58449842	6,915	9	Unknown	1	0	NA	NA	5	NA	92.7
1108	Un	58516038-58522861	6,824	21	Unknown	1	1	NA	67.7	1	NA	77.6
1112	Un	58547512-58554305	6,794	19	Unknown	1	0	NA	NA	1	NA	0
1124	Un	58642106-58648769	6,664	17	Unknown	1	2	NA	2	4	NA	0
1127	Un	58665171-58671783	6,613	16	Unknown	1	1	NA	26.1	0	NA	NA
1130	Un	58688105-58694710	6,606	15	Unknown	1	3	NA	2.8	7	NA	6.2
1132	Un	58703412-58709991	6,580	13	Unknown	1	0	NA	NA	4	NA	69.6
1141	Un	58775011-58781497	6,487	14	Unknown	1	2	NA	NA	4	NA	2
1142	Un	58782498-58788984	6,487	20	Unknown	1	1	NA	37.8	7	NA	37.6
1154	Un	58872130-58878547	6,418	13	Unknown	1	1	NA	68	1	NA	79
1156	Un	58886966-58893374	6,409	5	Unknown	1	3	NA	11.1	0	NA	NA
1160	Un	58916963-58923327	6,365	12	Unknown	1	1	NA	43.7	0	NA	NA
1170	Un	58991377-58997682	6,306	2	Unknown	1	1	NA	24.5	0	NA	NA
1173	Un	59013853-59020155	6,303	11	Unknown	1	1	NA	44.2	3	NA	63.5
1181	Un	59072976-59079224	6,249	20	Unknown	1	1	NA	7.8	1	NA	9.3
1194	Un	59172468-59178813	6,346	9	Unknown	1	0	NA	NA	1	NA	32.8
1199	Un	59208628-59214706	6,079	3	Unknown	1	1	NA	72.9	0	NA	NA
1204	Un	59244749-59250799	6,051	19	Unknown	1	2	NA	10.3	1	NA	3.3
1210	Un	59289613-59295617	6,005	21	Unknown	1	5	NA	7.1	2	NA	2.7
1212	Un	59303622-59309615	5,994	5	Unknown	1	0	NA	NA	2	NA	76.5
1217	Un	59338681-59344656	5,976	21	Unknown	1	1	NA	0	0	NA	NA
1226	Un	59401927-59407835	5,909	11	Unknown	1	0	NA	NA	1	NA	8.6
1238	Un	59485131-59490955	5,825	3	Unknown	1	3	NA	75	0	NA	NA
1249	Un	59564168-59569895	5,728	11	Unknown	1	1	NA	0	2	NA	0
1265	Un	59673974-59679712	5,739	17	Unknown	1	3	NA	0.3	0	NA	NA
1269	Un	59701145-59706743	5,599	20	Unknown	1	3	NA	21.6	4	NA	NA
1276	Un	59749913-59755470	5,558	13	Unknown	1	2	NA	64.3	0	NA	NA
1278	Un	59763027-59768578	5,552	4	Unknown	1	3	NA	0	0	NA	NA
1282	Un	59790253-59795762	5,510	15	Unknown	1	1	NA	0.3	2	NA	0
1285	Un	59810044-59815547	5,504	13	Unknown	1	1	NA	65.8	0	NA	NA

1286	Un	59816548-59822925	6,378	6	Unknown	1	0	NA	NA	2	NA	6.8
1287	Un	59823926-59829422	5,497	15	Unknown	1	1	NA	13.7	1	NA	NA
1290	Un	59843506-59848992	5,487	1	Unknown	1	2	NA	52.9	0	NA	NA
1292	Un	59858071-59863555	5,485	2	Unknown	1	3	NA	26.4	1	NA	37.8
1294	Un	59871040-59876620	5,581	6	Unknown	1	0	NA	NA	2	NA	14.7
1298	Un	59899551-59905001	5,451	17	Unknown	1	2	NA	2	2	NA	NA
1322	Un	60058027-60064074	6,048	12	Unknown	1	0	NA	NA	1	NA	40.1
1326	Un	60084738-60090014	5,277	11	Unknown	1	0	NA	NA	1	NA	5.3
1327	Un	60091015-60096434	5,420	4	Unknown	1	0	NA	NA	1	NA	50.4
1331	Un	60117280-60122507	5,228	7	Unknown	1	1	NA	8.2	1	NA	18.2
1332	Un	60123508-60128829	5,322	7	Unknown	1	1	NA	22.9	0	NA	NA
1334	Un	60136508-60141715	5,208	3	Unknown	1	0	NA	NA	1	NA	2.7
1336	Un	60148923-60154126	5,204	13	Unknown	1	1	NA	65.8	1	NA	80
1340	Un	60175586-60180783	5,198	18	Unknown	1	4	NA	31.7	0	NA	NA
1348	Un	60225181-60230339	5,159	7	Unknown	1	0	NA	NA	1	NA	95.4
1351	Un	60244643-60249779	5,137	13	Unknown	1	1	NA	16.1	0	NA	NA
1352	Un	60250780-60255915	5,136	12	Unknown	1	4	NA	NA	2	NA	19.9
1353	Un	60256916-60262050	5,135	6	Unknown	1	1	NA	60.1	0	NA	NA
1361	Un	60306588-60312291	5,704	21	Unknown	1	0	NA	NA	2	NA	10.1
1365	Un	60332199-60337369	5,171	15	Unknown	1	1	NA	7.4	1	NA	NA
1372	Un	60376086-60381181	5,096	13	Unknown	1	0	NA	NA	1	NA	76.2
1375	Un	60394914-60399882	4,969	12	Unknown	1	1	NA	29.6	0	NA	NA
1381	Un	60432449-60437367	4,919	8	Unknown	1	1	NA	60.8	0	NA	NA
1383	Un	60447261-60452371	5,111	6	Unknown	1	1	NA	60.5	0	NA	NA
1384	Un	60453372-60458274	4,903	1	Unknown	1	1	NA	71.6	4	NA	111.6
1395	Un	60522052-60526831	4,780	9	Unknown	1	0	NA	NA	2	NA	100
1405	Un	60583829-60589568	5,740	12	Unknown	1	0	NA	NA	2	NA	94.3
1430	Un	60736464-60740997	4,534	16	Unknown	1	1	NA	25.8	0	NA	NA
1532	Un	61339158-61344191	5,034	3	Unknown	1	1	NA	19.1	1	NA	27.1

Note (a): This scaffold mapped to two different chromosomes with the read correlation method (chromosome 16 in the FTC cross and chromosome 10 in the BEPA cross). This mapping is supported by 7 SNPs in the FTC cross, but the peak correlation is driven by a single SNP (chrUn:33,811,784). A BLAT search with a 100 base region surrounding this SNP maps to many regions of the genome, and the rest of the 6 FTC SNPs correlate with a region on chromosome 10. Therefore the likely location of this scaffold in both crosses is chromosome 10. Note (b): This scaffold also mapped to two different chromosomes with the read correlation method (chromosome 20 in the FTC cross and chromosome 12 in the BEPA cross). The BEPA marker is supported by 1 SNP (chrUn:46,516,721). A BLAT search with a 100 base region surrounding this SNP maps perfectly to many regions of the genome, including chromosome 12 and 20. Therefore the likely location of this scaffold in both crosses is chromosome 20.
