# Supplementary material for: Genome Assembly Improvement and Mapping Convergently Evolved Skeletal Traits in Sticklebacks with Genotyping-by-Sequencing
Source: G3 (Bethesda). 2015 Jun 3;5(7):1463–72. doi: 10.1534/g3.115.017905 (PMC4502380; doi:10.1534/g3.115.017905)
Supplement: Supporting Information [file supp_g3.115.017905_FigureS1.pdf]

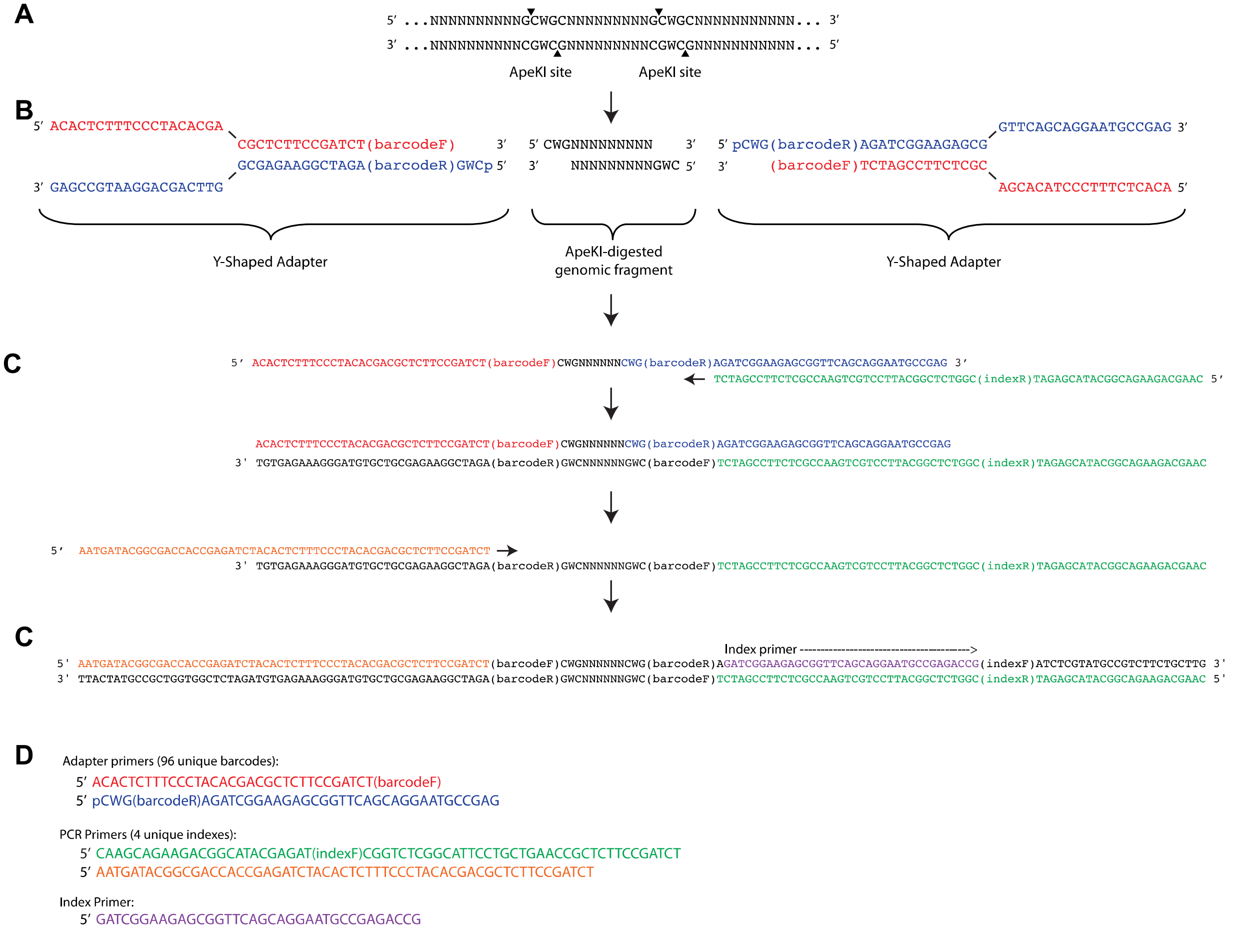

Figure S1 Diagram of library making method.
(A) Genomic DNA was digested with ApeKI, creating CWG overhangs. (B) Pairs of adapter primers were annealed together to create a “Y-shaped” primer, with partial base-pairing at one end. These adapters were ligated to both sides of the digested genomic DNA. 96 unique adapters were used, containing different internal barcode sequences (“barcodeF” and “barcodeR” together constitute the double-stranded barcode). (C) PCR primers with one of four index barcodes (“index”) are used to add sequence that will anneal to the Illumina flowcell. A custom index primer is used during sequencing to determine the index of each fragment, and the first bases of the R1 and R2 reads will contain the barcode. (D) Primers used in this study. See Table S1 for primer sequences.
