# Supplementary material for: Genome Assembly Improvement and Mapping Convergently Evolved Skeletal Traits in Sticklebacks with Genotyping-by-Sequencing
Source: G3 (Bethesda). 2015 Jun 3;5(7):1463–72. doi: 10.1534/g3.115.017905 (PMC4502380; doi:10.1534/g3.115.017905)
Supplement: Supporting Information [file supp_g3.115.017905_FigureS2.pdf]

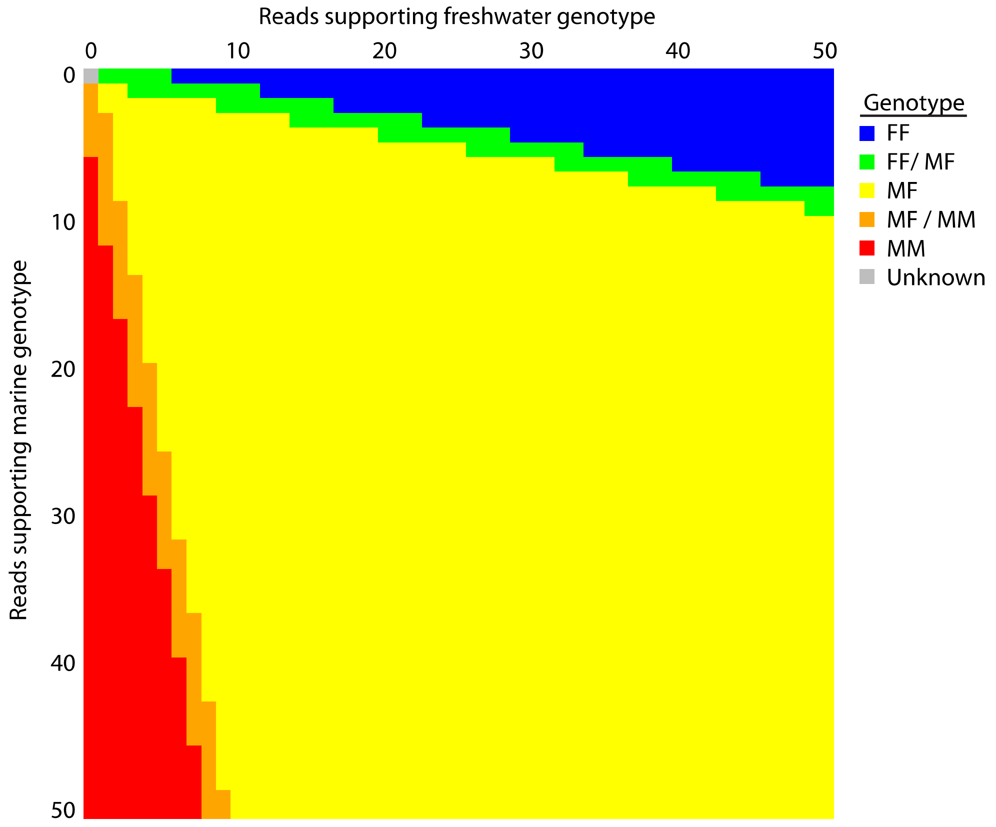

Figure S2 Genotype calling from marine and freshwater read counts
Reads were assumed to be independent and have a 1% error rate. M=marine allele, F=freshwater allele. The FF/MF and MF/MM genotypes indicate that there is a >95% probability that the genotype is not the third possible genotype, but that there is not a >95% certainty for any single genotype call. These genotypes were frequently called for bins that spanned recombination breakpoints.
