# Supplementary material for: Genome Assembly Improvement and Mapping Convergently Evolved Skeletal Traits in Sticklebacks with Genotyping-by-Sequencing
Source: G3 (Bethesda). 2015 Jun 3;5(7):1463–72. doi: 10.1534/g3.115.017905 (PMC4502380; doi:10.1534/g3.115.017905)
Supplement: Supporting Information [file supp_g3.115.017905_FigureS4.pdf]

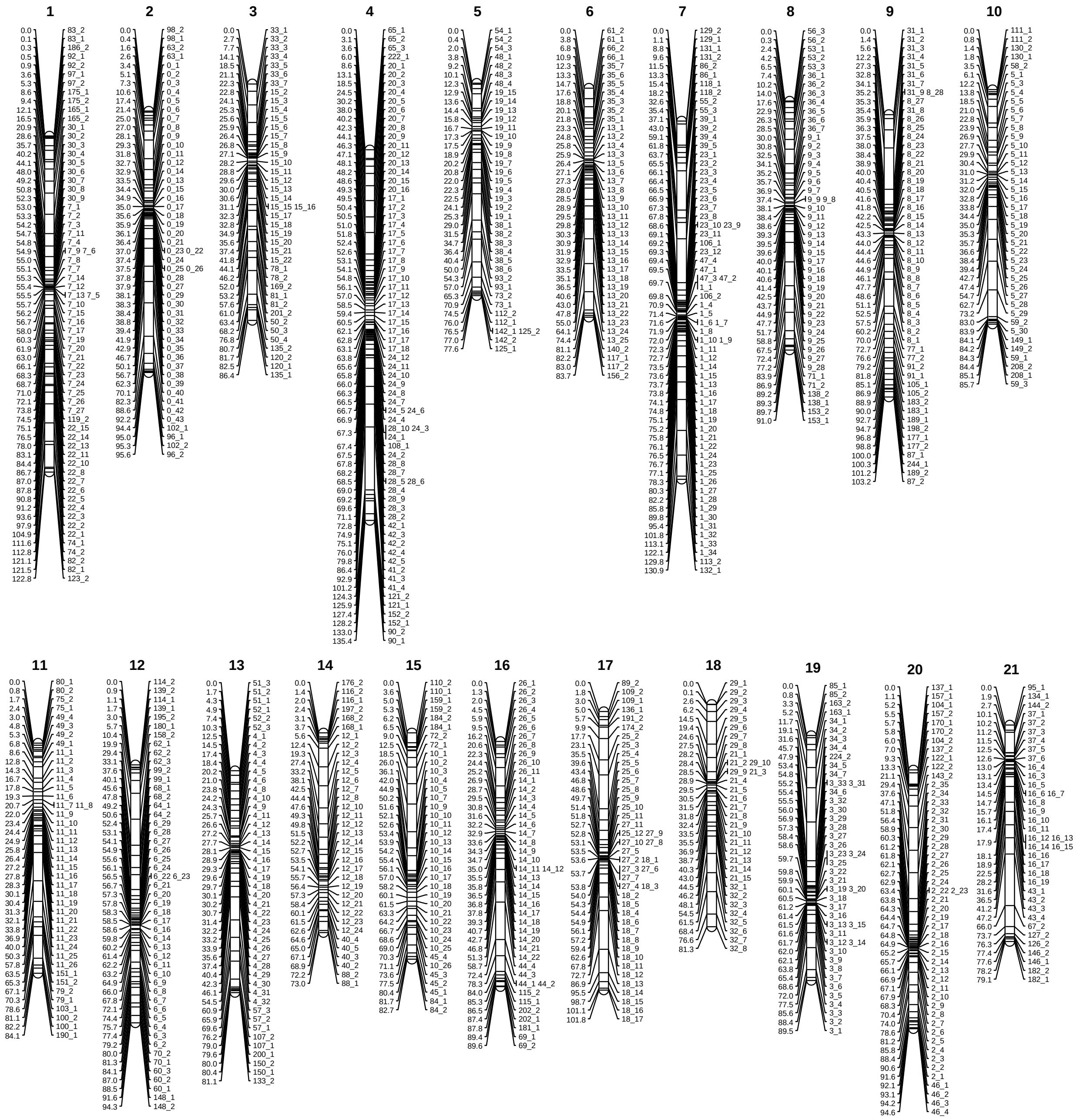

Figure S4 Linkage map from BEPA x LITC cross

Diagram of linkage map generated from the BEPA x LITC cross. For each chromosome, genetic position of each marker is shown on the left in centiMorgans, and marker name is shown on the right.
