# Supplementary material for: Genome Assembly Improvement and Mapping Convergently Evolved Skeletal Traits in Sticklebacks with Genotyping-by-Sequencing
Source: G3 (Bethesda). 2015 Jun 3;5(7):1463–72. doi: 10.1534/g3.115.017905 (PMC4502380; doi:10.1534/g3.115.017905)
Supplement: Supporting Information [file supp_g3.115.017905_FigureS5.pdf]

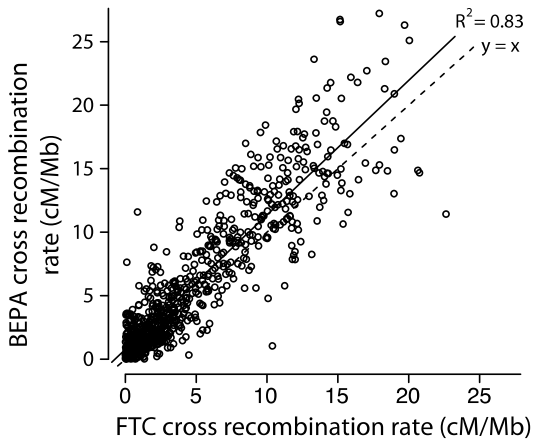

Figure S5 High correlation of recombination rates
Recombination rate in the FTC cross plotted against recombination rate in the BEPA cross. Each point is a marker. Recombination rates were calculated by taking the local slope of the loess-smoothed genetic vs. physical plots shown in Figure 3. Recombination rates are highly correlated (R^2 = 0.83). Solid line: best fit line from a linear regression; dotted line: y=x. Total genetic map size was smaller in the FTC cross (1569.9 cM) compared to the BEPA cross (1963.3 cM). Most points were displaced from the y=x line, indicating a higher recombination rate throughout the genome in the BEPA cross.
