# Supplementary material for: Genome Assembly Improvement and Mapping Convergently Evolved Skeletal Traits in Sticklebacks with Genotyping-by-Sequencing
Source: G3 (Bethesda). 2015 Jun 3;5(7):1463–72. doi: 10.1534/g3.115.017905 (PMC4502380; doi:10.1534/g3.115.017905)
Supplement: Supporting Information [file supp_g3.115.017905_FigureS6.pdf]

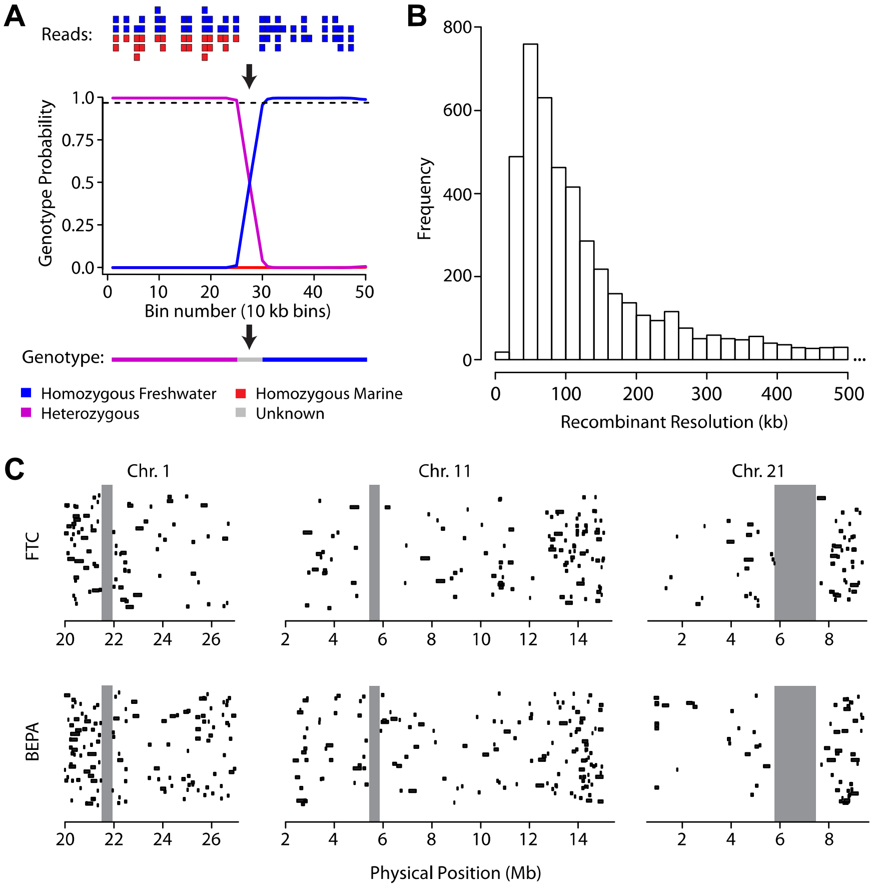

D

Start Probabilites:

M	0.25
H	0.50
F	0.25

Transition Probabilites:

		Hidden state (true genotype)		
		M	H	F
Hidden state (true genotype)	M	0.997	0.002	0.001
	H	0.001	0.998	0.001
	F	0.001	0.002	0.997

Emission Probabilities:

		Observed symbols (observed genotypes)			
		M	B	F	X
Hidden state (true genotype)	M	0.468	0.011	0.001	0.520
	H	0.082	0.317	0.085	0.516
	F	0.001	0.010	0.467	0.522

Figure S6 Fine-mapping recombinant breakpoints with a Hidden Markov Model

(A) Reads were binned into small 10 kilobase (kb) bins. For each bin, the probability of each genotype was determined, and boundaries of the recombination breakpoint were set at the 99% cutoff. (B) Histogram of recombination breakpoint resolution in the FTC x LITC cross. Median resolution was 89 kb. 539 additional recombinants (not shown) had a resolution of greater than 500 kb. (C) Diagram of high-resolution (<350 kb) recombination breakpoints near three marine/freshwater inversions. Each line represents the 99% confidence interval for a recombination breakpoint in a single fish and the grey rectangles represent the previously published marine/freshwater inversion boundaries (Jones et al. 2012). Data is presented for the entire scaffold containing each inversion (scaffold 22 on chromosome 1, scaffold 11 on chromosome 11, and scaffold 16 on chromosome 21). Physical position coordinates from the original assembly are used. No recombination events are observed within these three inversion intervals in each cross. (D) Trained Hidden Markov Model parameters used for fine-mapping recombination breakpoints. SNP genotypes were binned into 10 kb bins. For each fish, each bin was assigned a raw genotype of M (only marine reads), F (only freshwater reads), B (both marine and freshwater reads), or X (no data). These raw genotypes were used as the observed data in a Hidden Markov Model with hidden states M (marine), F (freshwater), or H (heterozygous). The model was trained with the Baum-Welch algorithm on 1 million data points.
