# Supplementary material for: Genome Assembly Improvement and Mapping Convergently Evolved Skeletal Traits in Sticklebacks with Genotyping-by-Sequencing
Source: G3 (Bethesda). 2015 Jun 3;5(7):1463–72. doi: 10.1534/g3.115.017905 (PMC4502380; doi:10.1534/g3.115.017905)
Supplement: Supporting Information [file supp_g3.115.017905_FigureS7.pdf]

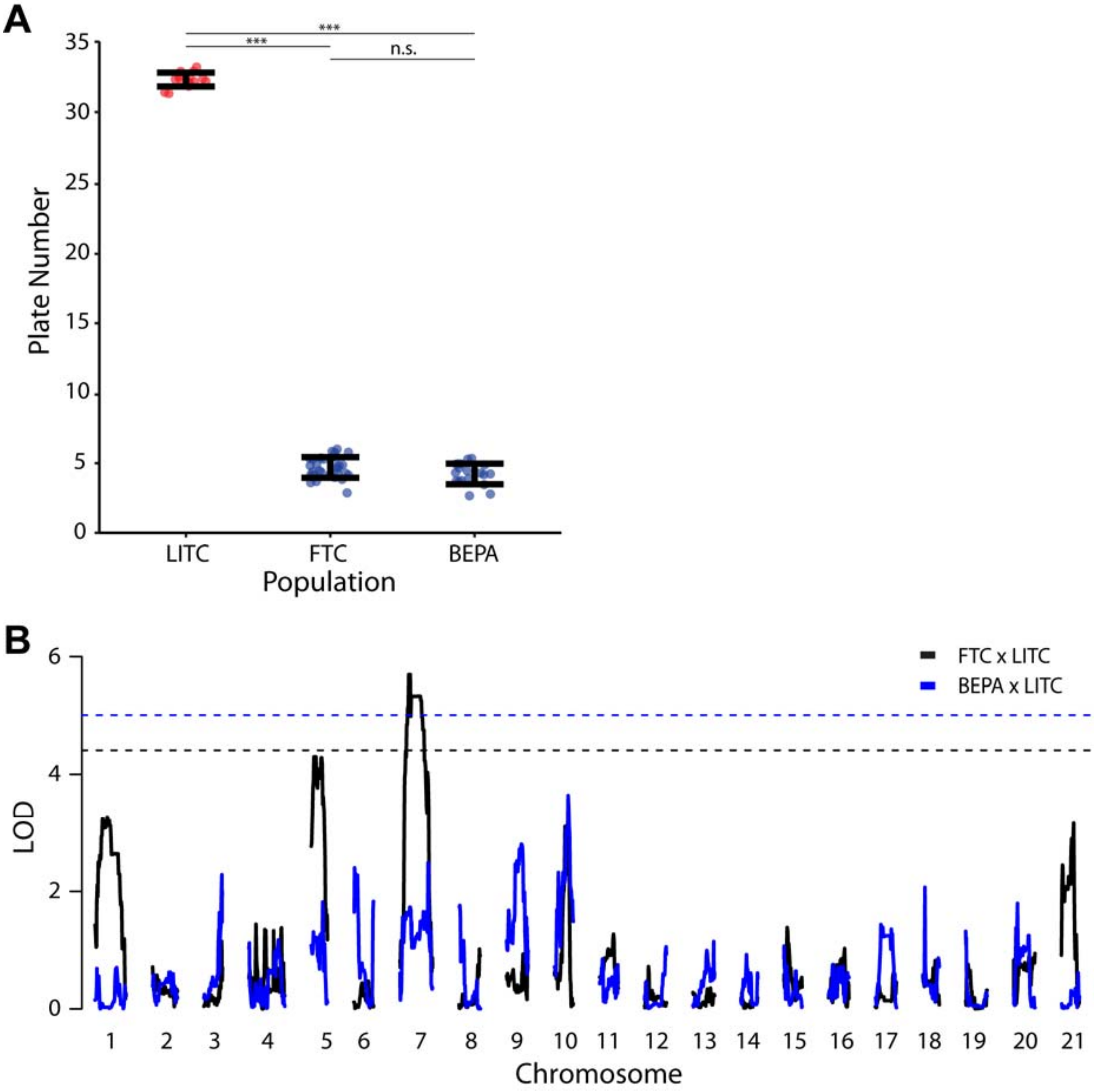

Figure S7 QTL mapping of lateral plate modifiers

(A) Lateral plate reduction in lab-reared FTC and BEPA freshwater fish (blue) compared to LITC marine fish (red). *** indicates $p<10^{-10}$, n.s. = not significant by Tukey's HSD test. Average of left and right side plates is shown. LITC, FTC, and BEPA fish had 32.3+/- 0.5, 4.9+/- 0.7, and 4.4+/- 0.7 plates and sample sizes of 15, 30, and 19, respectively. (B) Manhattan plot of QTL mapping of lateral plate modifiers in the FTC (black) and BEPA (blue) crosses. Mapping was performed in fish heterozygous for *Eda* (n=184/194 in the FTC/BEPA crosses). One QTL was detected in the FTC cross but no significant QTL were detected in the BEPA cross. α = 0.05 significance levels based on 1,000 permutations are shown as dotted lines. LOD is shown as a function of adjusted physical position.
