# Supplementary material for: Genome Assembly Improvement and Mapping Convergently Evolved Skeletal Traits in Sticklebacks with Genotyping-by-Sequencing
Source: G3 (Bethesda). 2015 Jun 3;5(7):1463–72. doi: 10.1534/g3.115.017905 (PMC4502380; doi:10.1534/g3.115.017905)
Supplement: Supporting Information [file supp_g3.115.017905_FigureS8.pdf]

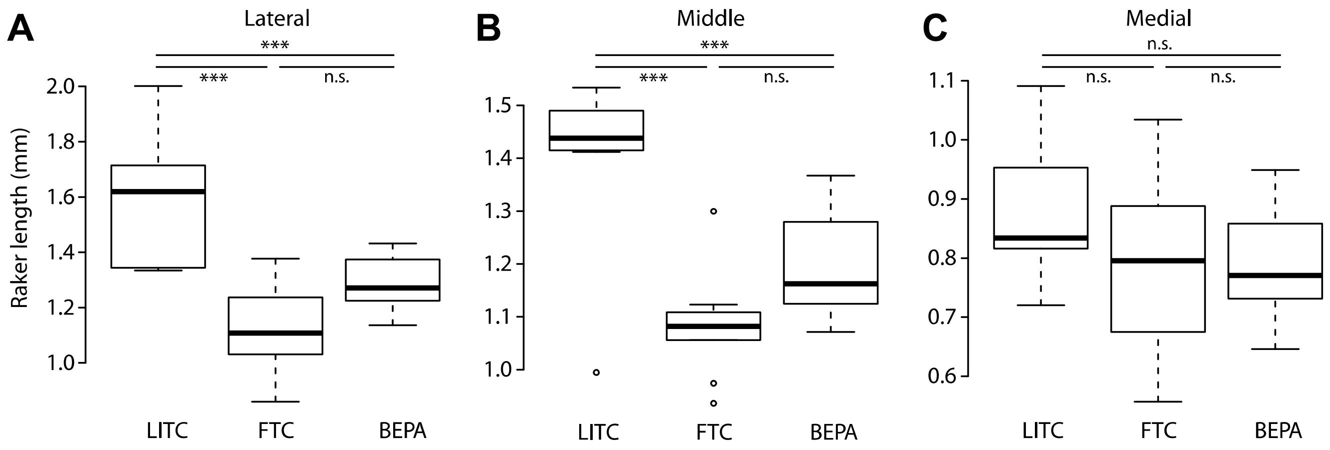

Figure S8 Convergent evolution of freshwater gill raker length reduction
Gill raker lengths were measured in three domains: lateral (A), middle (B), and medial (C) in marine (LITC) and freshwater (FTC and BEPA) lab-reared fish. See Figure 5A for a diagram of gill raker length measurements. Gill raker lengths were back transformed to values expected for a 50 mm standard length fish. *** indicates $p<0.001$, n.s. = not significant by Tukey's HSD test. n=10 per population.
