# Supplementary material for: Genome Assembly Improvement and Mapping Convergently Evolved Skeletal Traits in Sticklebacks with Genotyping-by-Sequencing
Source: G3 (Bethesda). 2015 Jun 3;5(7):1463–72. doi: 10.1534/g3.115.017905 (PMC4502380; doi:10.1534/g3.115.017905)
Supplement: Supporting Information [file supp_g3.115.017905_FileS5.pdf]

Files S5-S7

Data available from the Dryad Digital Repository: http://dx.doi.org/10.5061/dryad.q018v

File S5 Revised fasta file of genome assembly (.fa)

Fasta file containing revised genome assembly based on consensus scaffold order and orientation as described in File S4.

File S6 Revised masked fasta file of genome assembly (.fa)

Repeat masked fasta file containing revised genome assembly based on consensus scaffold order and orientation as described in File S4. Repeat masked fasta file is based off the repeat masked version of the original genome assembly, which was masked with RepeatMasker.

File S7 Revised locations of Ensembl-predicted genes (.gtf)

Revised .gtf file of Ensembl gene predictions. Coordinates of gene predictions were converted to the revised assembly coordinates. All Ensembl-predicted genes were included, except ENSGACT00000019430, which spans two scaffolds (11 and 79) that are not adjacent in the revised genome assembly.
