# Supplementary material for: Genome Assembly Improvement and Mapping Convergently Evolved Skeletal Traits in Sticklebacks with Genotyping-by-Sequencing
Source: G3 (Bethesda). 2015 Jun 3;5(7):1463–72. doi: 10.1534/g3.115.017905 (PMC4502380; doi:10.1534/g3.115.017905)
Supplement: Supporting Information [file supp_g3.115.017905_FileS8.pdf]

File S8

SUPPLEMENTAL METHODS

Cross raising

Lab-reared fish were raised at 18°C in 110 liter (29 gallon) aquaria in a common brackish salinity (3 ppt, 10% ocean water) and fed a common diet of live *Artemia* nauplii and frozen *Daphnia* as fry and juveniles, and bloodworms and *Mysis* shrimp as adults. Adult F2s were raised to a mean size (standard length) of 38.0 mm and 42.1 mm in the FTC and BEPA crosses, respectively. Fish from F1 crosses were intercrossed to generate F2 families ranging in size from 26-108 fish. Adult F2 fish (n= 360 / 363) were sequenced with GBS from 5 and 9 F2 families in the FTC and BEPA crosses, respectively.

Sequencing of cross grandparents and identification of homozygous SNPs

The two grandparents of the FTC cross and the two grandparents of the BEPA cross were resequenced with a Nextera DNA Sample Preparation kit (Illumina) to approximately 60X and 6X coverage, respectively, with 100 base paired-end sequencing on an Illumina HiSeq 2000 sequencer. Reads were mapped to the reference genome with BWA (www.bio-bwa.sourceforge.net). For each grandparent, SNPs relative to the reference genome were called with SAMtools (www.samtools.sourceforge.net). The SNPs in the resulting VCF files were then filtered with a custom Python script (www.python.org) to identify sites where one grandparent was homozygous for the alternate allele (a homozygous alternate SNP was present in the VCF file) and the other grandparent was homozygous for the reference allele (no SNP was present in the VCF file but there was sufficient sequencing coverage to support that the sample was homozygous reference). This analysis identified 1,116,087 and 715,279 sites which were homozygous different between the FTC and BEPA grandparents, respectively ("homozygous SNP positions").

Genotyping from read counts

A likelihood method was adapted from (Hohenlohe et al. 2010), but with only reads supporting the phased marine or freshwater allele considered. Three possible diploid genotypes were considered: G_i=G_{MM}, G_{MF}, or G_{FF} (M=marine allele, F=freshwater allele). Reads were considered to be independent, and have an equal chance of emanating from the two alleles. An error rate of 1% was assumed for each read, which increased the conservativeness of the genotyping calls. Bayes' theorem was used to calculate the probability of each genotype (G_i) given the observed pattern of reads. For example, for the MM genotype: $p(G_{MM}|reads)=p(reads|G_{MM})/[p(reads|G_{MM})+p(reads|G_{MF})+p(reads|G_{FF})]$. If one genotype G_i was most likely with $p(G_i|reads) > 0.95$, the genotype was assigned as G_i. Otherwise, the genotype was assigned as "MF/FF" or "MM/MF" if $p(G_{MM})<0.05$ or $p(G_{FF})<0.05$ (Figure S2). These genotypes were frequently called for bins spanning recombinant breakpoints. If no reads were present, a missing genotype ("NA") was assigned.

Removing markers with aberrant allele ratios

Each marker was tested for deviation from the predicted 1:2:1 allele ratio by a chi-square test. Multiple adjacent markers with allelic distortion likely indicate a true allelic distortion of that genomic region, whereas a single marker with allelic distortion likely indicates genotyping error. Therefore, markers showing significant allelic distortion ($p<0.05$) without any adjacent makers with allelic distortion were removed.

Sex determination

The sex of each F2 fish was determined by calculating the ratio of chromosome 19 (sex chromosome) coverage levels to non-chromosome 19 coverage levels. For females (XX) and males (XY), the theoretical ratios are 1 and 0.5, respectively, with a cutoff of 0.75. Indeed, in each cross the ratio cleanly formed two groups. However, there was slightly more sex chromosome coverage than expected in each cross. Therefore cutoff ratios in each cross were determined manually based on the empirical ratio distribution (0.85 in the FTC cross and 1.15 in the BEPA cross). Fish above and below the cutoff ratio were assigned as female and male, respectively.

Creating a consensus scaffold map

For each cross, the mean genetic positions of each scaffold in the linkage map were compared to create a scaffold map. In cases where multiple markers from the same scaffold were in the linkage map, scaffold orientation was determined by whether correlation between the physical and genetic positions of these markers was significantly positive or negative in a linear regression. A consensus scaffold map was created by merging the largely identical scaffold maps from the two crosses. In cases where the maps disagreed in marker order, the consensus map used the order from the map with the fewest discordant genotypes.

Recombinant breakpoint fine-mapping

Since the markers used for linkage mapping were based on binning multiple SNPs together, a complementary approach was used to fine-map recombinant breakpoints with a Hidden Markov Model (approach adapted from Andolfatto et al. 2011). SNP genotypes were binned into 10 kb bins, near the average density of SNPs used in the study. For each fish, each bin was assigned a raw genotype of M (only marine reads), F (only freshwater reads), B (both marine and freshwater reads), or X (no data). These raw genotypes were used as the observed data in a Hidden Markov Model with hidden states M (marine), F (freshwater), or H (heterozygous). The model was trained with the Baum-Welch algorithm on 1 million data points. For each fish and bin, the model was used to calculate the probability of each hidden state with the forward-backward algorithm. Details on the trained parameters of the model are presented in Figure S6D. Locations within a scaffold where the probability switched from over 99% probability of state A to over 99% probability of state B were considered to be the boundaries of a recombination event.

Calling sex chromosome genotypes

The stickleback sex chromosome (chromosome 19) consists of a small pseudo-autosomal region and a large region that behaves like a sex chromosome (Peichel et al. 2004; Roesti et al. 2013).

The cutoff for the boundary between these regions was chosen at 2.41 Mb, based on levels of sequencing coverage in male and female F2s. Genotypes for the pseudo-autosomal region were determined with the same method as the autosomal chromosomes, whereas a separate pipeline was used for the rest of the sex chromosome. First, reads that mapped to the X chromosome but not the Y chromosome were identified by sieving for SNPs that had an approximately 2:1 ratio of females to male coverage (SNPs that had female:male average RPMM ratios between 1.5 and 3.5). Female F2s should have a 3:1 ratio of X chromosomes originating from their grandmother:grandfather, whereas male F2s should have a 1:1 ratio. Therefore, a 3:1 ratio of alleles was filtered for in female F2s (average RPMM between 2 and 6) and a 1:1 ratio was filtered for in male F2s (average RPMM between 0.2 and 4). Finally, since all male F2s have a Y chromosome that originated from their grandfather, male F2 genotypes that were genotyped as homozygous for their grandmother's allele were converted to heterozygous.

Using read correlations to anchor scaffolds

In addition to the linkage maps made with JoinMap, a second more sensitive, but less precise, method was used to further determine the genomic location of scaffolds. This method enabled the mapping of even more scaffolds, but did not reveal the scaffolds' precise genomic location and orientation. Every pair of markers was considered ("marker 1" and "marker 2"). Fish genotyped as homozygous marine or homozygous freshwater for marker 1 were examined. For these fish, raw read counts for marker 2 were summed and were scored as concordant or discordant with the marker 1 genotype. If the total concordance percentage was above 95%, marker 2 was considered linked to marker 1 with ~<5 cM distance. In 300 out of 302 cases (99.3%) where a scaffold mapped to a chromosome by this method in both crosses, the chromosome was the same. The other two cases were likely due to reads mapping to repetitive genomic segments (Table S3).

Bone staining

To stain bones, fish were fixed for 1-2 days in 10% neutral buffered formalin or 5-7 days in 4% paraformaldehyde in 1xPBS, washed with water overnight, stained overnight with 0.008% Alizarin Red S in 1% potassium hydroxide, destained in water overnight, and cleared in a 0.25% potassium hydroxide, 50% glycerol solution.

Phenotype processing

For QTL mapping, plate number and gill raker length phenotypes were tested for an association with standard length and sex by linear regression in R (www.r-project.org) and corrected for size, sex, and/or log transformed, when appropriate. When association with standard length was significant ($p<0.05$), residuals were taken from a linear regression, then back-transformed to values expected for a 40 mm (marine x freshwater F2s) or 50 mm (lab-reared incross) standard length fish. When correlations with standard length and sex were both significant in a linear model, both were corrected for. When log transformation improved the normality of the residuals (i.e. by resulting in an Anderson-Darling test changing from $p<0.05$ to $p>0.05$), log transformation was performed.

REFERENCES

Andolfatto, P., D. Davison, D. Erezyilmaz, T.T. Hu, J. Mast *et al.*, 2011 Multiplexed shotgun genotyping for rapid and efficient genetic mapping. Genome Res. 21: 610-617.

Hohenlohe, P.A., S. Bassham, P.D. Etter, N. Stiffler, E.A. Johnson *et al.*, 2010 Population genomics of parallel adaptation in threespine stickleback using sequenced RAD tags. PLoS Genet. 6: e1000862.

Jones, F.C., M.G. Grabherr, Y.F. Chan, P. Russell, E. Mauceli *et al.*, 2012 The genomic basis of adaptive evolution in threespine sticklebacks. Nature 484: 55-61.

Peichel, C.L., J.A. Ross, C.K. Matson, M. Dickson, J. Grimwood *et al.*, 2004 The master sex-determination locus in threespine sticklebacks is on a nascent Y chromosome. Current Biology 14: 1416-1424.

Roesti, M., D. Moser, and D. Berner, 2013 Recombination in the threespine stickleback genome--patterns and consequences. Mol. Ecol. 22: 3014-3027.
