# Supplementary material for: Genome Assembly Improvement and Mapping Convergently Evolved Skeletal Traits in Sticklebacks with Genotyping-by-Sequencing
Source: G3 (Bethesda). 2015 Jun 3;5(7):1463–72. doi: 10.1534/g3.115.017905 (PMC4502380; doi:10.1534/g3.115.017905)
Supplement: Supporting Information [file supp_g3.115.017905_TableS1.pdf]

Table S1 Primers used in this study. Primers were used for PCR, index sequencing ("Index"), or making adapters.

Name	Primer	Type	Barcode
PCR1	CAAGCAGAAGACGGCATACGAGATCGGTCTCGGCATTCCTGCTGAAC CGCTCTTCCGATCT	PCR (no indexing)	NA
PCR1_index1	CAAGCAGAAGACGGCATACGAGATcgtgatCGGTCTCGGCATTCCTGC TGAACCGCTCTTCCGATCT	PCR (indexing)	NA
PCR1_index2	CAAGCAGAAGACGGCATACGAGATacatcgCGGTCTCGGCATTCCTGC TGAACCGCTCTTCCGATCT	PCR (indexing)	NA
PCR1_index3	CAAGCAGAAGACGGCATACGAGATgcctaaCGGTCTCGGCATTCCTGC TGAACCGCTCTTCCGATCT	PCR (indexing)	NA
PCR1_index4	CAAGCAGAAGACGGCATACGAGATtggtcaCGGTCTCGGCATTCCTGC TGAACCGCTCTTCCGATCT	PCR (indexing)	NA
PCR2	AATGATACGGCGACCACCGAGATCTACACTCTTTCCCTACACGACGCT CTTCCGATCT	PCR (indexing/no indexing)	NA
Seq_index	GATCGGAAGAGCGGTTCAGCAGGAATGCCGAGACCG	Index	NA
ApeK1Y_A1F	ACACTCTTTCCCTACACGACGCTCTTCCGATCTctcc	Adapter	ctcc
ApeK1Y_A2F	ACACTCTTTCCCTACACGACGCTCTTCCGATCTtgca	Adapter	tgca
ApeK1Y_A3F	ACACTCTTTCCCTACACGACGCTCTTCCGATCTacta	Adapter	acta
ApeK1Y_A4F	ACACTCTTTCCCTACACGACGCTCTTCCGATCTcaga	Adapter	caga
ApeK1Y_A5F	ACACTCTTTCCCTACACGACGCTCTTCCGATCTaact	Adapter	aact
ApeK1Y_A6F	ACACTCTTTCCCTACACGACGCTCTTCCGATCTgcgt	Adapter	gcgt
ApeK1Y_A7F	ACACTCTTTCCCTACACGACGCTCTTCCGATCTcgat	Adapter	cgat
ApeK1Y_A8F	ACACTCTTTCCCTACACGACGCTCTTCCGATCTgtaa	Adapter	gtaa
ApeK1Y_A9F	ACACTCTTTCCCTACACGACGCTCTTCCGATCTtgcga	Adapter	tgcga
ApeK1Y_A10F	ACACTCTTTCCCTACACGACGCTCTTCCGATCTcgctt	Adapter	cgctt
ApeK1Y_A11F	ACACTCTTTCCCTACACGACGCTCTTCCGATCTtcacc	Adapter	tcacc
ApeK1Y_A12F	ACACTCTTTCCCTACACGACGCTCTTCCGATCTctagc	Adapter	ctagc
ApeK1Y_B1F	ACACTCTTTCCCTACACGACGCTCTTCCGATCTacaaa	Adapter	acaaa
ApeK1Y_B2F	ACACTCTTTCCCTACACGACGCTCTTCCGATCTttctc	Adapter	ttctc
ApeK1Y_B3F	ACACTCTTTCCCTACACGACGCTCTTCCGATCTagccc	Adapter	agccc
ApeK1Y_B4F	ACACTCTTTCCCTACACGACGCTCTTCCGATCTgtatt	Adapter	gtatt
ApeK1Y_B5F	ACACTCTTTCCCTACACGACGCTCTTCCGATCTctgta	Adapter	ctgta
ApeK1Y_B6F	ACACTCTTTCCCTACACGACGCTCTTCCGATCTaccgt	Adapter	accgt
ApeK1Y_B7F	ACACTCTTTCCCTACACGACGCTCTTCCGATCTgctta	Adapter	gctta
ApeK1Y_B8F	ACACTCTTTCCCTACACGACGCTCTTCCGATCTggtgt	Adapter	ggtgt
ApeK1Y_B9F	ACACTCTTTCCCTACACGACGCTCTTCCGATCTggttgt	Adapter	ggttgt
ApeK1Y_B10F	ACACTCTTTCCCTACACGACGCTCTTCCGATCTccagct	Adapter	ccagct
ApeK1Y_B11F	ACACTCTTTCCCTACACGACGCTCTTCCGATCTttcaga	Adapter	ttcaga
ApeK1Y_B12F	ACACTCTTTCCCTACACGACGCTCTTCCGATCTtaggaa	Adapter	taggaa
ApeK1Y_C1F	ACACTCTTTCCCTACACGACGCTCTTCCGATCTgctcta	Adapter	gctcta
ApeK1Y_C2F	ACACTCTTTCCCTACACGACGCTCTTCCGATCTccacaa	Adapter	ccacaa
ApeK1Y_C3F	ACACTCTTTCCCTACACGACGCTCTTCCGATCTcttcca	Adapter	cttcca
ApeK1Y_C4F	ACACTCTTTCCCTACACGACGCTCTTCCGATCTgagata	Adapter	gagata
ApeK1Y_C5F	ACACTCTTTCCCTACACGACGCTCTTCCGATCTatgcct	Adapter	atgcct
ApeK1Y_C6F	ACACTCTTTCCCTACACGACGCTCTTCCGATCTagtgga	Adapter	agtgga
ApeK1Y_C7F	ACACTCTTTCCCTACACGACGCTCTTCCGATCTtattttt	Adapter	tattttt
ApeK1Y_C8F	ACACTCTTTCCCTACACGACGCTCTTCCGATCTcttgctt	Adapter	cttgctt
ApeK1Y_C9F	ACACTCTTTCCCTACACGACGCTCTTCCGATCTatgaaac	Adapter	atgaaac
ApeK1Y_C10F	ACACTCTTTCCCTACACGACGCTCTTCCGATCTaaaagtt	Adapter	aaaagtt
ApeK1Y_C11F	ACACTCTTTCCCTACACGACGCTCTTCCGATCTgaattca	Adapter	gaattca
ApeK1Y_C12F	ACACTCTTTCCCTACACGACGCTCTTCCGATCTgaacttc	Adapter	gaacttc
ApeK1Y_D1F	ACACTCTTTCCCTACACGACGCTCTTCCGATCTggaccta	Adapter	ggaccta
ApeK1Y_D2F	ACACTCTTTCCCTACACGACGCTCTTCCGATCTgtcgatt	Adapter	gtcgatt
ApeK1Y_D3F	ACACTCTTTCCCTACACGACGCTCTTCCGATCTaacgcct	Adapter	aacgcct
ApeK1Y_D4F	ACACTCTTTCCCTACACGACGCTCTTCCGATCTaatatgc	Adapter	aatatgc
ApeK1Y_D5F	ACACTCTTTCCCTACACGACGCTCTTCCGATCTacgactac	Adapter	acgactac

ApeK1Y_D6F	ACACTCTTTCCCTACACGACGCTCTTCCGATCTtagcatgc	Adapter	tagcatgc
ApeK1Y_D7F	ACACTCTTTCCCTACACGACGCTCTTCCGATCTtaggccat	Adapter	taggccat
ApeK1Y_D8F	ACACTCTTTCCCTACACGACGCTCTTCCGATCTtgcaagga	Adapter	tgcaagga
ApeK1Y_D9F	ACACTCTTTCCCTACACGACGCTCTTCCGATCTtggtacgt	Adapter	tggtacgt
ApeK1Y_D10F	ACACTCTTTCCCTACACGACGCTCTTCCGATCTtctcagtc	Adapter	tctcagtc
ApeK1Y_D11F	ACACTCTTTCCCTACACGACGCTCTTCCGATCTccggatat	Adapter	ccggatat
ApeK1Y_D12F	ACACTCTTTCCCTACACGACGCTCTTCCGATCTcgccttat	Adapter	cgccttat
ApeK1Y_E1F	ACACTCTTTCCCTACACGACGCTCTTCCGATCTaggc	Adapter	aggc
ApeK1Y_E2F	ACACTCTTTCCCTACACGACGCTCTTCCGATCTgatc	Adapter	gatc
ApeK1Y_E3F	ACACTCTTTCCCTACACGACGCTCTTCCGATCTtcac	Adapter	tcac
ApeK1Y_E4F	ACACTCTTTCCCTACACGACGCTCTTCCGATCTaggat	Adapter	aggat
ApeK1Y_E5F	ACACTCTTTCCCTACACGACGCTCTTCCGATCTattga	Adapter	attga
ApeK1Y_E6F	ACACTCTTTCCCTACACGACGCTCTTCCGATCTcatct	Adapter	catct
ApeK1Y_E7F	ACACTCTTTCCCTACACGACGCTCTTCCGATCTcctac	Adapter	cctac
ApeK1Y_E8F	ACACTCTTTCCCTACACGACGCTCTTCCGATCTgagga	Adapter	gagga
ApeK1Y_E9F	ACACTCTTTCCCTACACGACGCTCTTCCGATCTggaac	Adapter	ggaac
ApeK1Y_E10F	ACACTCTTTCCCTACACGACGCTCTTCCGATCTgtcaa	Adapter	gtcaa
ApeK1Y_E11F	ACACTCTTTCCCTACACGACGCTCTTCCGATCTtaata	Adapter	taata
ApeK1Y_E12F	ACACTCTTTCCCTACACGACGCTCTTCCGATCTtacat	Adapter	tacat
ApeK1Y_F1F	ACACTCTTTCCCTACACGACGCTCTTCCGATCTtcgtt	Adapter	tcgtt
ApeK1Y_F2F	ACACTCTTTCCCTACACGACGCTCTTCCGATCTacctaa	Adapter	acctaa
ApeK1Y_F3F	ACACTCTTTCCCTACACGACGCTCTTCCGATCTatatgt	Adapter	atatgt
ApeK1Y_F4F	ACACTCTTTCCCTACACGACGCTCTTCCGATCTatcgta	Adapter	atcgta
ApeK1Y_F5F	ACACTCTTTCCCTACACGACGCTCTTCCGATCTcatcgt	Adapter	catcgt
ApeK1Y_F6F	ACACTCTTTCCCTACACGACGCTCTTCCGATCTcgcggt	Adapter	cgcggt
ApeK1Y_F7F	ACACTCTTTCCCTACACGACGCTCTTCCGATCTctatta	Adapter	ctatta
ApeK1Y_F8F	ACACTCTTTCCCTACACGACGCTCTTCCGATCTgccagt	Adapter	gccagt
ApeK1Y_F9F	ACACTCTTTCCCTACACGACGCTCTTCCGATCTggaaga	Adapter	ggaaga
ApeK1Y_F10F	ACACTCTTTCCCTACACGACGCTCTTCCGATCTgtactt	Adapter	gtactt
ApeK1Y_F11F	ACACTCTTTCCCTACACGACGCTCTTCCGATCTgttgaa	Adapter	gttgaa
ApeK1Y_F12F	ACACTCTTTCCCTACACGACGCTCTTCCGATCTtaacga	Adapter	taacga
ApeK1Y_G1F	ACACTCTTTCCCTACACGACGCTCTTCCGATCTtggcta	Adapter	tggcta
ApeK1Y_G2F	ACACTCTTTCCCTACACGACGCTCTTCCGATCTacgtgtt	Adapter	acgtgtt
ApeK1Y_G3F	ACACTCTTTCCCTACACGACGCTCTTCCGATCTattaatt	Adapter	attaatt
ApeK1Y_G4F	ACACTCTTTCCCTACACGACGCTCTTCCGATCTattggat	Adapter	attggat
ApeK1Y_G5F	ACACTCTTTCCCTACACGACGCTCTTCCGATCTcataagt	Adapter	cataagt
ApeK1Y_G6F	ACACTCTTTCCCTACACGACGCTCTTCCGATCTcgctgat	Adapter	cgctgat
ApeK1Y_G7F	ACACTCTTTCCCTACACGACGCTCTTCCGATCTcggtaga	Adapter	cggtaga
ApeK1Y_G8F	ACACTCTTTCCCTACACGACGCTCTTCCGATCTctacgga	Adapter	ctacgga
ApeK1Y_G9F	ACACTCTTTCCCTACACGACGCTCTTCCGATCTgcggaat	Adapter	gcggaat
ApeK1Y_G10F	ACACTCTTTCCCTACACGACGCTCTTCCGATCTtagcgga	Adapter	tagcgga
ApeK1Y_G11F	ACACTCTTTCCCTACACGACGCTCTTCCGATCTtcgaaga	Adapter	tcgaaga
ApeK1Y_G12F	ACACTCTTTCCCTACACGACGCTCTTCCGATCTtctgtga	Adapter	tctgtga
ApeK1Y_H1F	ACACTCTTTCCCTACACGACGCTCTTCCGATCTtgctgga	Adapter	tgctgga
ApeK1Y_H2F	ACACTCTTTCCCTACACGACGCTCTTCCGATCTaaccgaga	Adapter	aaccgaga
ApeK1Y_H3F	ACACTCTTTCCCTACACGACGCTCTTCCGATCTacagggaa	Adapter	acagggaa
ApeK1Y_H4F	ACACTCTTTCCCTACACGACGCTCTTCCGATCTacgtggta	Adapter	acgtggta
ApeK1Y_H5F	ACACTCTTTCCCTACACGACGCTCTTCCGATCTccatgggt	Adapter	ccatgggt
ApeK1Y_H6F	ACACTCTTTCCCTACACGACGCTCTTCCGATCTcgcggaga	Adapter	cgcggaga
ApeK1Y_H7F	ACACTCTTTCCCTACACGACGCTCTTCCGATCTcgtgtggt	Adapter	cgtgtggt
ApeK1Y_H8F	ACACTCTTTCCCTACACGACGCTCTTCCGATCTgctgtgga	Adapter	gctgtgga
ApeK1Y_H9F	ACACTCTTTCCCTACACGACGCTCTTCCGATCTggattggt	Adapter	ggattggt
ApeK1Y_H10F	ACACTCTTTCCCTACACGACGCTCTTCCGATCTgtgagggt	Adapter	gtgagggt
ApeK1Y_H11F	ACACTCTTTCCCTACACGACGCTCTTCCGATCTtatcggga	Adapter	tatcggga
ApeK1Y_H12F	ACACTCTTTCCCTACACGACGCTCTTCCGATCTttcctgga	Adapter	ttcctgga
ApeK1Y_A1R	pCWGggagAGATCGGAAGAGCGGTTCAGCAGGAATGCCGAG	Adapter	NA
ApeK1Y_A2R	pCWGtgcaAGATCGGAAGAGCGGTTCAGCAGGAATGCCGAG	Adapter	NA
ApeK1Y_A3R	pCWGtagtAGATCGGAAGAGCGGTTCAGCAGGAATGCCGAG	Adapter	NA

ApeK1Y_A4R	pCWGtctgAGATCGGAAGAGCGGTTCAGCAGGAATGCCGAG	Adapter	NA
ApeK1Y_A5R	pCWGagttAGATCGGAAGAGCGGTTCAGCAGGAATGCCGAG	Adapter	NA
ApeK1Y_A6R	pCWGacgcAGATCGGAAGAGCGGTTCAGCAGGAATGCCGAG	Adapter	NA
ApeK1Y_A7R	pCWGatcgAGATCGGAAGAGCGGTTCAGCAGGAATGCCGAG	Adapter	NA
ApeK1Y_A8R	pCWGttacAGATCGGAAGAGCGGTTCAGCAGGAATGCCGAG	Adapter	NA
ApeK1Y_A9R	pCWGtcgcaAGATCGGAAGAGCGGTTCAGCAGGAATGCCGAG	Adapter	NA
ApeK1Y_A10R	pCWGaagcgAGATCGGAAGAGCGGTTCAGCAGGAATGCCGAG	Adapter	NA
ApeK1Y_A11R	pCWGggtgaAGATCGGAAGAGCGGTTCAGCAGGAATGCCGAG	Adapter	NA
ApeK1Y_A12R	pCWGgctagAGATCGGAAGAGCGGTTCAGCAGGAATGCCGAG	Adapter	NA
ApeK1Y_B1R	pCWGtttgtAGATCGGAAGAGCGGTTCAGCAGGAATGCCGAG	Adapter	NA
ApeK1Y_B2R	pCWGgagaaAGATCGGAAGAGCGGTTCAGCAGGAATGCCGAG	Adapter	NA
ApeK1Y_B3R	pCWGgggctAGATCGGAAGAGCGGTTCAGCAGGAATGCCGAG	Adapter	NA
ApeK1Y_B4R	pCWGaatacAGATCGGAAGAGCGGTTCAGCAGGAATGCCGAG	Adapter	NA
ApeK1Y_B5R	pCWGtacagAGATCGGAAGAGCGGTTCAGCAGGAATGCCGAG	Adapter	NA
ApeK1Y_B6R	pCWGacggtAGATCGGAAGAGCGGTTCAGCAGGAATGCCGAG	Adapter	NA
ApeK1Y_B7R	pCWGtaagcAGATCGGAAGAGCGGTTCAGCAGGAATGCCGAG	Adapter	NA
ApeK1Y_B8R	pCWGacaccAGATCGGAAGAGCGGTTCAGCAGGAATGCCGAG	Adapter	NA
ApeK1Y_B9R	pCWGacaaccAGATCGGAAGAGCGGTTCAGCAGGAATGCCGAG	Adapter	NA
ApeK1Y_B10R	pCWGagctggAGATCGGAAGAGCGGTTCAGCAGGAATGCCGAG	Adapter	NA
ApeK1Y_B11R	pCWGtctgaaAGATCGGAAGAGCGGTTCAGCAGGAATGCCGAG	Adapter	NA
ApeK1Y_B12R	pCWGttcctaAGATCGGAAGAGCGGTTCAGCAGGAATGCCGAG	Adapter	NA
ApeK1Y_C1R	pCWGtagagcAGATCGGAAGAGCGGTTCAGCAGGAATGCCGAG	Adapter	NA
ApeK1Y_C2R	pCWGttgtggAGATCGGAAGAGCGGTTCAGCAGGAATGCCGAG	Adapter	NA
ApeK1Y_C3R	pCWGtggaagAGATCGGAAGAGCGGTTCAGCAGGAATGCCGAG	Adapter	NA
ApeK1Y_C4R	pCWGtatctcAGATCGGAAGAGCGGTTCAGCAGGAATGCCGAG	Adapter	NA
ApeK1Y_C5R	pCWGaggcatAGATCGGAAGAGCGGTTCAGCAGGAATGCCGAG	Adapter	NA
ApeK1Y_C6R	pCWGtccactAGATCGGAAGAGCGGTTCAGCAGGAATGCCGAG	Adapter	NA
ApeK1Y_C7R	pCWGaaaaataAGATCGGAAGAGCGGTTCAGCAGGAATGCCGAG	Adapter	NA
ApeK1Y_C8R	pCWGaagcaagAGATCGGAAGAGCGGTTCAGCAGGAATGCCGAG	Adapter	NA
ApeK1Y_C9R	pCWGgtttcatAGATCGGAAGAGCGGTTCAGCAGGAATGCCGAG	Adapter	NA
ApeK1Y_C10R	pCWGaacttttAGATCGGAAGAGCGGTTCAGCAGGAATGCCGAG	Adapter	NA
ApeK1Y_C11R	pCWGtgaattcAGATCGGAAGAGCGGTTCAGCAGGAATGCCGAG	Adapter	NA
ApeK1Y_C12R	pCWGgaagttcAGATCGGAAGAGCGGTTCAGCAGGAATGCCGAG	Adapter	NA
ApeK1Y_D1R	pCWGtaggtccAGATCGGAAGAGCGGTTCAGCAGGAATGCCGAG	Adapter	NA
ApeK1Y_D2R	pCWGaatcgacAGATCGGAAGAGCGGTTCAGCAGGAATGCCGAG	Adapter	NA
ApeK1Y_D3R	pCWGaggcgttAGATCGGAAGAGCGGTTCAGCAGGAATGCCGAG	Adapter	NA
ApeK1Y_D4R	pCWGgcatattAGATCGGAAGAGCGGTTCAGCAGGAATGCCGAG	Adapter	NA
ApeK1Y_D5R	pCWGgtagtcgtAGATCGGAAGAGCGGTTCAGCAGGAATGCCGAG	Adapter	NA
ApeK1Y_D6R	pCWGgcatgctaAGATCGGAAGAGCGGTTCAGCAGGAATGCCGAG	Adapter	NA
ApeK1Y_D7R	pCWGatggcctaAGATCGGAAGAGCGGTTCAGCAGGAATGCCGAG	Adapter	NA
ApeK1Y_D8R	pCWGtccttgcaAGATCGGAAGAGCGGTTCAGCAGGAATGCCGAG	Adapter	NA
ApeK1Y_D9R	pCWGacgtaccaAGATCGGAAGAGCGGTTCAGCAGGAATGCCGAG	Adapter	NA
ApeK1Y_D10R	pCWGgactgagaAGATCGGAAGAGCGGTTCAGCAGGAATGCCGAG	Adapter	NA
ApeK1Y_D11R	pCWGatatccggAGATCGGAAGAGCGGTTCAGCAGGAATGCCGAG	Adapter	NA
ApeK1Y_D12R	pCWGataaggcgAGATCGGAAGAGCGGTTCAGCAGGAATGCCGAG	Adapter	NA
ApeK1Y_E1R	pCWGgcctAGATCGGAAGAGCGGTTCAGCAGGAATGCCGAG	Adapter	NA
ApeK1Y_E2R	pCWGgatcAGATCGGAAGAGCGGTTCAGCAGGAATGCCGAG	Adapter	NA
ApeK1Y_E3R	pCWGgtgaAGATCGGAAGAGCGGTTCAGCAGGAATGCCGAG	Adapter	NA
ApeK1Y_E4R	pCWGatcctAGATCGGAAGAGCGGTTCAGCAGGAATGCCGAG	Adapter	NA
ApeK1Y_E5R	pCWGtcaatAGATCGGAAGAGCGGTTCAGCAGGAATGCCGAG	Adapter	NA
ApeK1Y_E6R	pCWGagatgAGATCGGAAGAGCGGTTCAGCAGGAATGCCGAG	Adapter	NA
ApeK1Y_E7R	pCWGgtaggAGATCGGAAGAGCGGTTCAGCAGGAATGCCGAG	Adapter	NA
ApeK1Y_E8R	pCWGtcctcAGATCGGAAGAGCGGTTCAGCAGGAATGCCGAG	Adapter	NA
ApeK1Y_E9R	pCWGgttccAGATCGGAAGAGCGGTTCAGCAGGAATGCCGAG	Adapter	NA
ApeK1Y_E10R	pCWGttgacAGATCGGAAGAGCGGTTCAGCAGGAATGCCGAG	Adapter	NA
ApeK1Y_E11R	pCWGtattaAGATCGGAAGAGCGGTTCAGCAGGAATGCCGAG	Adapter	NA
ApeK1Y_E12R	pCWGatgtaAGATCGGAAGAGCGGTTCAGCAGGAATGCCGAG	Adapter	NA
ApeK1Y_F1R	pCWGaacgaAGATCGGAAGAGCGGTTCAGCAGGAATGCCGAG	Adapter	NA

ApeK1Y_F2R	pCWGttaggtAGATCGGAAGAGCGGTTCAGCAGGAATGCCGAG	Adapter	NA
ApeK1Y_F3R	pCWGacatatAGATCGGAAGAGCGGTTCAGCAGGAATGCCGAG	Adapter	NA
ApeK1Y_F4R	pCWGtacgatAGATCGGAAGAGCGGTTCAGCAGGAATGCCGAG	Adapter	NA
ApeK1Y_F5R	pCWGacgatgAGATCGGAAGAGCGGTTCAGCAGGAATGCCGAG	Adapter	NA
ApeK1Y_F6R	pCWGaccgcgAGATCGGAAGAGCGGTTCAGCAGGAATGCCGAG	Adapter	NA
ApeK1Y_F7R	pCWGtaatagAGATCGGAAGAGCGGTTCAGCAGGAATGCCGAG	Adapter	NA
ApeK1Y_F8R	pCWGactggcAGATCGGAAGAGCGGTTCAGCAGGAATGCCGAG	Adapter	NA
ApeK1Y_F9R	pCWGtcttccAGATCGGAAGAGCGGTTCAGCAGGAATGCCGAG	Adapter	NA
ApeK1Y_F10R	pCWGaagtacAGATCGGAAGAGCGGTTCAGCAGGAATGCCGAG	Adapter	NA
ApeK1Y_F11R	pCWGttcaacAGATCGGAAGAGCGGTTCAGCAGGAATGCCGAG	Adapter	NA
ApeK1Y_F12R	pCWGtcgttaAGATCGGAAGAGCGGTTCAGCAGGAATGCCGAG	Adapter	NA
ApeK1Y_G1R	pCWGtagccaAGATCGGAAGAGCGGTTCAGCAGGAATGCCGAG	Adapter	NA
ApeK1Y_G2R	pCWGaacacgtAGATCGGAAGAGCGGTTCAGCAGGAATGCCGAG	Adapter	NA
ApeK1Y_G3R	pCWGaattaatAGATCGGAAGAGCGGTTCAGCAGGAATGCCGAG	Adapter	NA
ApeK1Y_G4R	pCWGatccaatAGATCGGAAGAGCGGTTCAGCAGGAATGCCGAG	Adapter	NA
ApeK1Y_G5R	pCWGacttatgAGATCGGAAGAGCGGTTCAGCAGGAATGCCGAG	Adapter	NA
ApeK1Y_G6R	pCWGatcagcgAGATCGGAAGAGCGGTTCAGCAGGAATGCCGAG	Adapter	NA
ApeK1Y_G7R	pCWGtctaccgAGATCGGAAGAGCGGTTCAGCAGGAATGCCGAG	Adapter	NA
ApeK1Y_G8R	pCWGtccgtagAGATCGGAAGAGCGGTTCAGCAGGAATGCCGAG	Adapter	NA
ApeK1Y_G9R	pCWGattccgcAGATCGGAAGAGCGGTTCAGCAGGAATGCCGAG	Adapter	NA
ApeK1Y_G10R	pCWGtccgctaAGATCGGAAGAGCGGTTCAGCAGGAATGCCGAG	Adapter	NA
ApeK1Y_G11R	pCWGtcttcgaAGATCGGAAGAGCGGTTCAGCAGGAATGCCGAG	Adapter	NA
ApeK1Y_G12R	pCWGtcacagaAGATCGGAAGAGCGGTTCAGCAGGAATGCCGAG	Adapter	NA
ApeK1Y_H1R	pCWGtccagcaAGATCGGAAGAGCGGTTCAGCAGGAATGCCGAG	Adapter	NA
ApeK1Y_H2R	pCWGtctcggttAGATCGGAAGAGCGGTTCAGCAGGAATGCCGAG	Adapter	NA
ApeK1Y_H3R	pCWGttccctgtAGATCGGAAGAGCGGTTCAGCAGGAATGCCGAG	Adapter	NA
ApeK1Y_H4R	pCWGtaccacgtAGATCGGAAGAGCGGTTCAGCAGGAATGCCGAG	Adapter	NA
ApeK1Y_H5R	pCWGacccatggAGATCGGAAGAGCGGTTCAGCAGGAATGCCGAG	Adapter	NA
ApeK1Y_H6R	pCWGtctccgcgAGATCGGAAGAGCGGTTCAGCAGGAATGCCGAG	Adapter	NA
ApeK1Y_H7R	pCWGaccacacgAGATCGGAAGAGCGGTTCAGCAGGAATGCCGAG	Adapter	NA
ApeK1Y_H8R	pCWGtccacagcAGATCGGAAGAGCGGTTCAGCAGGAATGCCGAG	Adapter	NA
ApeK1Y_H9R	pCWGaccaatccAGATCGGAAGAGCGGTTCAGCAGGAATGCCGAG	Adapter	NA
ApeK1Y_H10R	pCWGaccctcacAGATCGGAAGAGCGGTTCAGCAGGAATGCCGAG	Adapter	NA
ApeK1Y_H11R	pCWGtcccgataAGATCGGAAGAGCGGTTCAGCAGGAATGCCGAG	Adapter	NA
ApeK1Y_H12R	pCWGtccaggaaAGATCGGAAGAGCGGTTCAGCAGGAATGCCGAG	Adapter	NA
