# Supplementary material for: Genome Assembly Improvement and Mapping Convergently Evolved Skeletal Traits in Sticklebacks with Genotyping-by-Sequencing
Source: G3 (Bethesda). 2015 Jun 3;5(7):1463–72. doi: 10.1534/g3.115.017905 (PMC4502380; doi:10.1534/g3.115.017905)
Supplement: Supporting Information [file supp_g3.115.017905_TableS2.pdf]

Table S2 Cross summary statistics. Numbers of initial samples, dropped samples due to low coverage, and final samples are presented. Numbers of initial markers, dropped markers due to low coverage and skewed allele ratios, and final markers are presented. GP=grandparent. Genotype fail % = percentage of missing genotypes.

			Samples			Markers						
Cross	GP SNPs	F2 SNPs used	Initial	Low coverage	Final	Initial	Low coverage	Skewed allele ratio	Final	SNP Coverage	Marker Coverage	Genotype Fail %
FTC	1,116,087	131,091	358	2	356	1,085	59	25	1,001	1.63X	197X	1.9
BEPA	715,279	87,419	361	2	359	1,077	39	60	978	1.73X	140X	1.8
