# Supplementary material for: Genome Assembly Improvement and Mapping Convergently Evolved Skeletal Traits in Sticklebacks with Genotyping-by-Sequencing
Source: G3 (Bethesda). 2015 Jun 3;5(7):1463–72. doi: 10.1534/g3.115.017905 (PMC4502380; doi:10.1534/g3.115.017905)
Supplement: Corrigendum [file supp_5_7_1463_v2_index.html]

Corrigendum 

# Genome Assembly Improvement and Mapping Convergently Evolved Skeletal Traits in Sticklebacks with Genotyping-by-Sequencing

## Corrigendum for Glazer *et al*., *G3* 5 (7): 1463-1472

**Files in this Data Supplement:**

- Corrigendum - Corrigendum for Glazer *et al*., *G3* 5 (7): 1463-1472
