# Supplementary material for: Genome Assembly Improvement and Mapping Convergently Evolved Skeletal Traits in Sticklebacks with Genotyping-by-Sequencing
Source: G3 (Bethesda). 2015 Jun 3;5(7):1463–72. doi: 10.1534/g3.115.017905 (PMC4502380; doi:10.1534/g3.115.017905)
Supplement: Corrigendum [file supp_g3.115.017905_Corrigendum_for_Glazer_et_al_FINAL.pdf]

Corrigendum for Glazer *et al*., *G3* 5 (7): 1463-1472.

G3: Genes | Genomes | Genetics, Vol 5, 1463-1472, July 2015, Copyright © Glazer et al.

CORRIGENDUM:
In the article by A. M. Glazer, E. E. Killingbeck, T. Mitros, D. S. Rokhsar, and C. T. Miller (*G3: Genes|Genomes|Genetics* 5: 1463–1472) entitled "Genome Assembly Improvement and Mapping Convergently Evolved Skeletal Traits in Sticklebacks with Genotyping-by-Sequencing," a citation was inadvertently omitted from the subsection "Preparation of GBS libraries" in the Materials and Methods.

Thus, the sentence

"Libraries 4–6 used 96 ApeKI Y-shaped adapters with internal barcodes, and library 7 used these 96 Y-shaped adapters and 4 different PCR primers with different index barcodes (384 total samples) (adapted from Peterson et al. 2012) (Table S1, Figure S1)."

has been modified to read

"Libraries 4–6 used 96 ApeKI Y-shaped adapters with internal barcodes (ICGMC 2015), and library 7 used these 96 Y-shaped adapters and 4 different PCR primers with different index barcodes (384 total samples) (adapted from Peterson et al. 2012) (Table S1, Figure S1)."

and the below citation was added to the Literature Cited.

International Cassava Genetic Map Consortium (ICGMC), 2015 High-Resolution Linkage Map and Chromosome-Scale Genome Assembly for Cassava (*Manihot esculenta* Crantz) from 10 Populations. G3 (Bethesda) 5: 133-144.
